# FARS2 Deficiency Causes Cardiomyopathy by Disrupting Mitochondrial Homeostasis and the Mitochondrial Quality Control System

**DOI:** 10.1161/CIRCULATIONAHA.123.064489

**Published:** 2024-02-16

**Authors:** Bowen Li, Fangfang Liu, Xihui Chen, Tangdong Chen, Juan Zhang, Yifeng Liu, Yan Yao, Weihong Hu, Mengjie Zhang, Bo Wang, Liwen Liu, Kun Chen, Yuanming Wu

**Affiliations:** 1 Department of Biochemistry and Molecular Biology, Shaanxi Provincial Key Laboratory of Clinical Genetics (B.L., X.C., T.C., J.Z., Y.L., Y.Y., W.H., M.Z., Y.W.), Air Force Medical University, Xi’an, China.; 2 Department of Neurobiology (F.L.), Air Force Medical University, Xi’an, China.; 3 Department of Anatomy, Histology and Embryology and K.K. Leung Brain Research Center (K.C.), Air Force Medical University, Xi’an, China.; 4 School of Basic Medicine, Department of Ultrasound, Xijing Hypertrophic Cardiomyopathy Center, Xijing Hospital (B.W., L.L.), Air Force Medical University, Xi’an, China.; 5 Department of Clinical Laboratory, Tangdu Hospital (Y.W.), Air Force Medical University, Xi’an, China.

**Keywords:** autophagy, cardiomyopathies, heart failure, mitochondrial dynamics, mitochondrial dysfunction, phenylalanyl-tRNA synthetase, tRNA aminoacylation

## Abstract

**BACKGROUND:**

Hypertrophic cardiomyopathy (HCM) is a common heritable heart disease. Although HCM has been reported to be associated with many variants of genes involved in sarcomeric protein biomechanics, pathogenic genes have not been identified in patients with partial HCM. FARS2 (the mitochondrial phenylalanyl-tRNA synthetase), a type of mitochondrial aminoacyl-tRNA synthetase, plays a role in the mitochondrial translation machinery. Several variants of *FARS2* have been suggested to cause neurological disorders; however, FARS2-associated diseases involving other organs have not been reported. We identified *FARS2* as a potential novel pathogenic gene in cardiomyopathy and investigated its effects on mitochondrial homeostasis and the cardiomyopathy phenotype.

**METHODS:**

*FARS2* variants in patients with HCM were identified using whole-exome sequencing, Sanger sequencing, molecular docking analyses, and cell model investigation. *Fars2* conditional mutant (p.R415L) or knockout mice, *fars2*-knockdown zebrafish, and *Fars2*-knockdown neonatal rat ventricular myocytes were engineered to construct FARS2 deficiency models both in vivo and in vitro. The effects of FARS2 and its role in mitochondrial homeostasis were subsequently evaluated using RNA sequencing and mitochondrial functional analyses. Myocardial tissues from patients were used for further verification.

**RESULTS:**

We identified 7 unreported *FARS2* variants in patients with HCM. Heart-specific *Fars2*-deficient mice presented cardiac hypertrophy, left ventricular dilation, progressive heart failure accompanied by myocardial and mitochondrial dysfunction, and a short life span. Heterozygous cardiac-specific *Fars2*^*R415L*^ mice displayed a tendency to cardiac hypertrophy at age 4 weeks, accompanied by myocardial dysfunction. In addition, *fars2*-knockdown zebrafish presented pericardial edema and heart failure. FARS2 deficiency impaired mitochondrial homeostasis by directly blocking the aminoacylation of mt-tRNA^Phe^ and inhibiting the synthesis of mitochondrial proteins, ultimately contributing to an imbalanced mitochondrial quality control system by accelerating mitochondrial hyperfragmentation and disrupting mitochondrion-related autophagy. Interfering with the mitochondrial quality control system using adeno-associated virus 9 or specific inhibitors mitigated the cardiac and mitochondrial dysfunction triggered by FARS2 deficiency by restoring mitochondrial homeostasis.

**CONCLUSIONS:**

Our findings unveil the previously unrecognized role of *FARS2* in heart and mitochondrial homeostasis. This study may provide new insights into the molecular diagnosis and prevention of heritable cardiomyopathy as well as therapeutic options for FARS2-associated cardiomyopathy.

**Clinical Perspective**

**What Is New?**

- This study identified *FARS2* as a potential pathogenic gene of heritable cardiomyopathy.
- *FARS2* ablation resulted in mitochondrial dysfunction, induced myocardial dysfunction, and heart failure, and ultimately led to sudden death.
- FARS2 (mitochondrial phenylalanyl-tRNA synthetase) deficiency promoted cardiac dysfunction by disrupting the mitochondrial quality control (MQC) system, whereas MQC system intervention attenuated cardiomyopathy triggered by FARS2 deficiency.

**What Are the Clinical Implications?**

- *FARS2* may represent a novel genetic candidate gene for preventing heritable cardiomyopathy.
- FARS2 is critical for maintaining MQC system homeostasis and normal cardiac function.
- Normalizing the MQC system, either through gene replacement therapy or small molecules, may be a novel therapeutic approach for FARS2-associated cardiomyopathy.

Hypertrophic cardiomyopathy (HCM), affecting ≈1 in 500 individuals, is the most common heritable cardiomyopathy.^1^ Characterized by asymmetric thickening of the left ventricle and impaired diastolic function, HCM is a leading cause of heart failure (HF) and sudden cardiac death.^2^ Advances in molecular genetics over the past 20 years have unveiled >1000 genetic variants associated with HCM, most of which are related to sarcomeric genes, such as *MYH7* and *MYBPC3*.^3^ However, a substantial proportion of patients with HCM do not present any evidence of a known genetic variation.^4^ Therefore, other novel pathogenic variants might be responsible for or contribute to the HCM phenotype in these patients.^4,5^

Early studies demonstrated that most known HCM genetic variants lead to higher myocardial energy demand.^6^ The imbalance between inefficient energy supply and increased ATP demand leads to adverse remodeling and contributes to the progression of HCM.^7^ Mitochondria play a key role in normal cardiac energy metabolism through oxidative phosphorylation (OXPHOS).^8^ Dysfunction of mitochondrial OXPHOS has been described in models and patients with HCM.^9,10^ Despite evidence linking mitochondrial functional alterations and pathological progression in HCM, the genetic variants in mitochondrial translation machinery have not received substantial attention.

Independent of the cytoplasm, the mitochondrion possesses translation machinery regulated by mitochondrial DNA (mtDNA)–encoded tRNA, ribosomal RNA, and nuclear DNA–encoded mitochondrial aminoacyl-tRNA synthetases (mtARS).^11^ Only 13 of >1500 mitochondrial proteins are synthesized in the mitochondria. These 13 proteins constitute the key subunits in complexes of OXPHOS.^12^ mtARS are responsible for the mitochondrial protein synthesis that involves covalently pairing a tRNA with its cognate amino acids.^13^ Widespread application of whole-genome sequencing revealed multiple variants in mtARS-encoding genes associated with diseases, which are mostly observed in organs requiring high energy.^11,13^ Despite the common biochemical role of enzymes, wide-ranging clinical manifestations involving different physiological systems have been reported in patients with mtARS variants.^11^ Nevertheless, variants of mtARS-encoding genes are largely linked to the central nervous system.^11,13^ Only some variants, including *AARS2*, *VARS2*, *PARS2*, and *YARS2*, have been identified to be responsible for cardiomyopathy in humans with an unclear pathological mechanism.^14–17^ All the known variants of *FARS2* have been implicated only in central nervous system–related disorders, such as hereditary spastic paraplegia.^18^ The correlation of *FARS2* variants with other systemic diseases has not been reported.^13^

Maintenance of mitochondrial function, morphology, and mass, also known as mitochondrial homeostasis, is essential for cardiac function and aging.^19^ The mitochondrial quality control (MQC) system is a robust coordinator that maintains mitochondrial homeostasis under basal conditions and various stress conditions, such as cardiac remodeling.^20,21^ Complex signaling pathways constitute the MQC system, including mitochondrial fission, fusion, and mitochondrion-related autophagy.^20,22^ Studies have revealed the role of key functional molecules of the MQC system in cardiac function and stress-induced remodeling.^23^ However, the molecular mechanism underlying mtARS-related HCM remains unknown, as is the role of the changes in mitochondrial homeostasis and the MQC system caused by mtARS deficiency.

We aimed to unveil the previously unrecognized role of FARS2 in the heart. These findings provide novel insights into the prevention and screening of pathogenic genes in heritable cardiomyopathy and suggest that targeting the MQC system might confer new therapeutic benefits on FARS2 (mitochondrial phenylalanyl-tRNA synthetase)–associated cardiomyopathy.

## METHODS

All study samples were obtained from patients at XiJing Hospital who provided written informed consent. The study received research ethics board approval from Xijing Hospital. All animal studies followed the guidelines of the animal care committee of the Fourth Military Medical University. The related materials are available from the corresponding author upon reasonable request. Detailed Methods are provided in the Supplemental Material.

### Statistical Analysis

Data were analyzed with GraphPad Prism (version 9.0.0) and expressed as mean±SEM. Statistical analyses were performed using ANOVA followed by the Tukey test for multiple-group comparisons and the Student *t* test for 2-group comparisons. Statistical significance was set at *P*<0.05.

## RESULTS

### Identification and Functional Consequences of Monoallelic *FARS2* Variants in HCM

**Figure 1.**
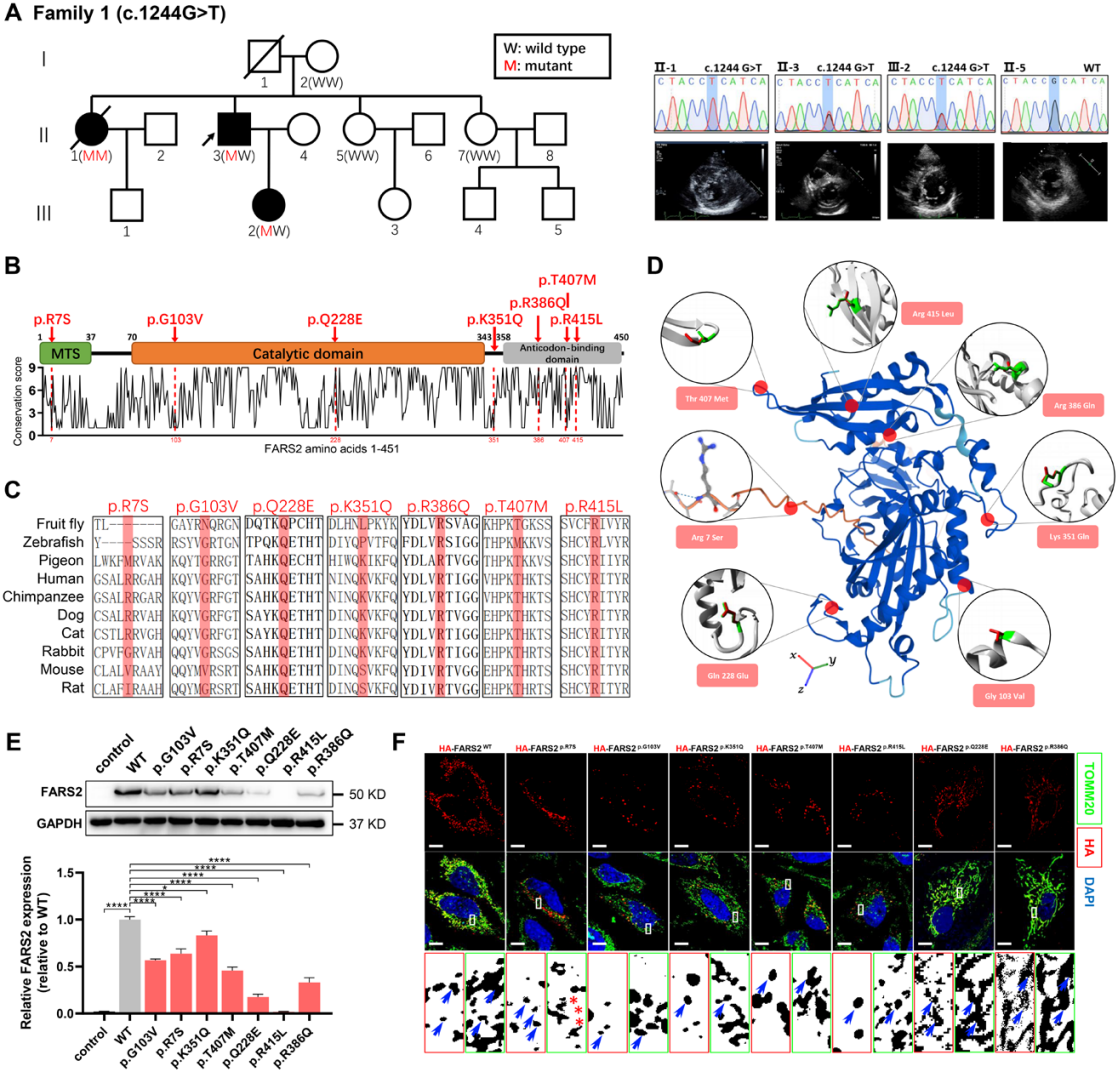
**Functional characterization of *FARS2* variants identified in hypertrophic cardiomyopathy. A**, Pedigrees. Sanger validation of *FARS2* variant and echocardiography of the patients in family 1. **B**, A graphical illustration of *FARS2* sequence conservation (**bottom**) based on ConSurf conservation score. The domain structure and positions of identified patient variants (red symbols) are indicated. **C**, Species conservation of FARS2 (mitochondrial phenylalanyl-tRNA synthetase) amino acids p.R7, p.G103, p.Q228, p.K351, p.R386, p.T407, and p.R415. **D**, A structural model of wild-type (WT) human FARS2 (AlphaFold Protein Structure Database O95363 [SYFM_HUMAN]), highlighting the sites of FARS2 variants (p.Arg7Ser, p.Gly103Val, p.Gln228Glu, p.Lys351Gln, p.Arg386Gln, p.Thr407Met, and p.Arg415Leu). **E**, The expression level of FARS2 from HeLa cells transfected with empty vector control, WT, or 7 variant vectors. The relative statistical analysis is shown in the **bottom** panel (n=3 per group). **F**, Confocal images of HeLa cells transfected with HA-FARS2 constructs and immunolabeled with TOMM20 (mitochondrial marker). The regions were magnified with split channels beneath each group and boxed with red (HA) or green (TOMM20). The representative colocalization parts are emphasized by blue arrows; the noncolocalization parts are highlighted by red asterisks. Scale bar=10 μm. *P<0.05; ****P<0.0001.

A family with HCM was recruited (Figure 1A). Based on whole exome sequencing analysis of the family (I-2, II-3, II-7, and III-2), a monoallelic variant of *FARS2* (c.1244G>T, NM_001318872.2; p.Arg415Leu) was identified as a potential pathogenic variant on the basis of the cosegregation analysis and Sanger sequencing validation (Figure 1A; Figure S1A through S1C; Table S1). However, the reason for the homozygosity of FARS2^R415L^ in II-1 was not available because of the lack of a sufficient genome sample from individuals I-1 and II-1 (who are deceased).

To determine the correlation between *FARS2* and HCM, we screened for additional *FARS2* variants from a library comprising 1141 patients with HCM and found 6 other variants of *FARS2* (c.21G>T [p.Arg7Ser], c.308G>T [p.Gly103Val], c.682C>G [p.Gln228Glu], c.1051A>C [p.Lys351Gln], c.1157G>A [p.Arg386Gln], and c.1220C>T [p.Thr407Met]) from 7 patients with HCM without a family history (Figure 1B through 1D; Figure S2; Table). We first summarized the in silico predictions, minor allele frequency (MAF), and the results of case–control association studies (Tables S2 and S3). We further pooled all available evidence for pathogenic classification of these variants according to the American College of Medical Genetics and Genomics and Association for Molecular Pathology guidelines (Table S4).^24^ Based on these results, all 7 variants were categorized as variants of uncertain significance (Table S4).

**Table.**
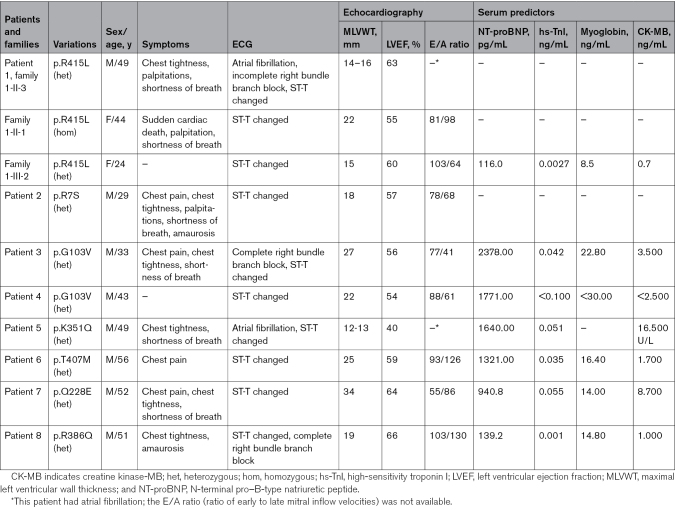
Clinical, Genetic, ECG, and Echocardiographic Characteristics

To determine whether there is a common feature of the variants, HA-tagged variants and wild-type (WT) *FARS2* were individually expressed in human cell lines (HeLa and A549). Compared with WT, these variants showed lower expression (Figure 1E and 1F; Figure S3A and S3B), partly because of decreased protein stability (Figure S3C through S3E; Table S5). Lower FARS2 expression was also detected in the myocardial tissues of 2 patients (c.308G>T, c.1244G>T; Figure S8A and S8B). Furthermore, we engineered a heterozygous cardiac-specific FARS2^R415L^ mouse model (*cMut/*−; Figure S4A through S4D). Compared with controls, the *cMut/*− mice had lower FARS2 expression and undetected FARS2^R415L^ abundance in the heart (Figure S4E). In addition, whereas Arg.7 was located at the mitochondrial target sequence, the other 6 residues were located at or close to the pivotal functional domain of FARS2 (Figure 1B through 1D). We performed molecular docking analyses to assess enzyme activity by predicting the binding energy between the variants and ATP (or mt-tRNA^Phe^); we found that the ATP-binding and tRNA-aminoacylation activities of each variant decreased in varying degrees (Figure S5A through S5C; Table S6). Only the partial FARS2^p.R7S^ variant showed no colocalization with the mitochondria (Figure 1F; Figure S3B). Overall, these results indicate that all patient-identified *FARS2* variants had lower FARS2 expression, damaged enzyme activity, or impaired mitochondrial localization, consistent with FARS2 deficiency.

### FARS2 Is Essential for Normal Cardiac Function

To elucidate the role of FARS2 in the heart, we first analyzed the RNA sequencing data of multiple types of cardiomyopathies and HF in human and mouse models. FARS2 expression was substantially downregulated in HF and cardiomyopathies, including HCM (Figure S6), demonstrating that FARS2 deficiency may be the pathogenic mechanism of cardiomyopathies and HF.

**Figure 2.**
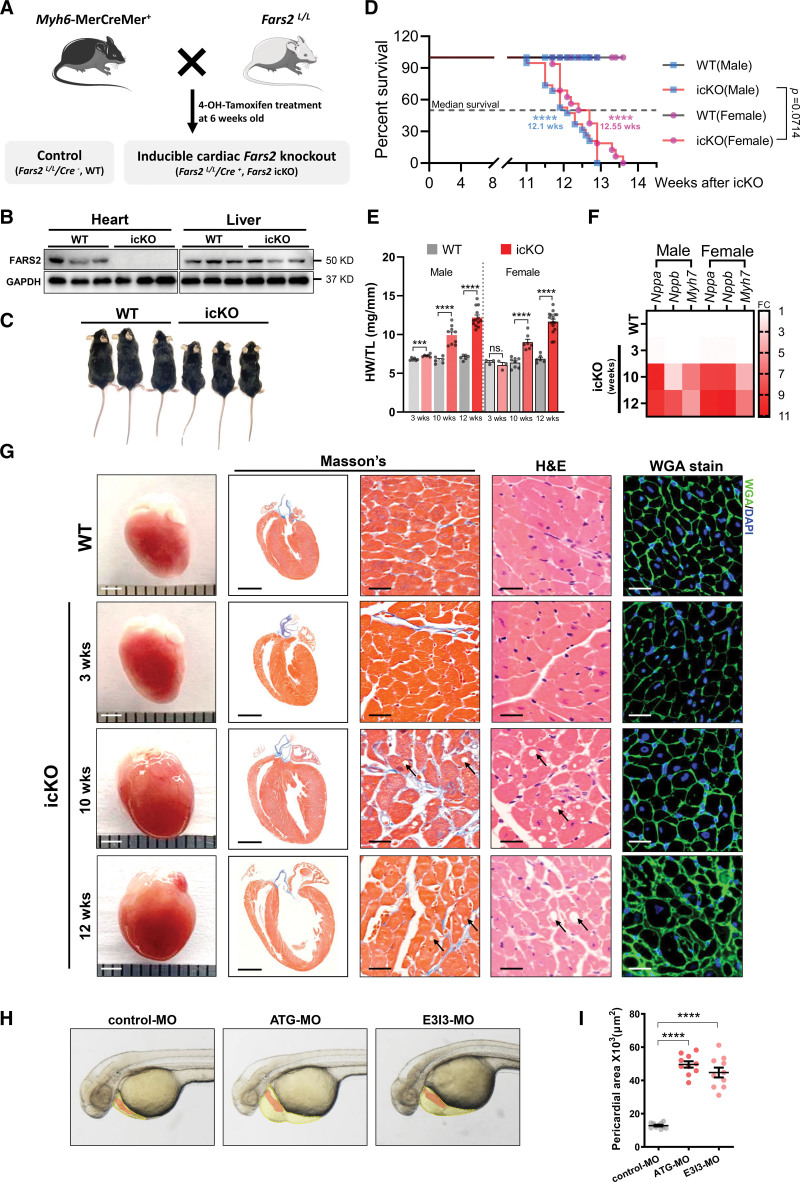
**FARS2 deficiency causes cardiac hypertrophy leading to heart failure and sudden death in vivo. A**, Schematic diagram to generate 4-OH tamoxifen inducible cardiac-specific *Fars2* knockout (icKO) mice by crossing *Fars2*^*LoxP/LoxP*^ mice with *Myh6*-MerCreMer^+^ mice. **B**, Western blot analysis of FARS2 (mitochondrial phenylalanyl-tRNA synthetase) protein levels in the heart and liver of wild-type (WT) control and FARS2 icKO mice, with GAPDH as loading control. **C**, Photograph of WT and icKO male mice at 12 weeks after icKO. **D**, Kaplan-Meier survival curves for WT and icKO mice (n=16–19). The median survival times for the icKO group are marked. **E**, Ratios of heart weight to tibia length (HW/TL; n=3–16). **F**, Relative mRNA levels of cardiac hypertrophy markers (*Nppa*, *Nppb*, and *Myh7*) in mice 3, 10, and 12 weeks after icKO (n=3). **G**, Representative heart (scale bar=2 mm), longitudinal sections (scale bar=2 mm), Masson trichrome staining (scale bar=25 μm), hematoxylin & eosin staining (scale bar=25 μm), and wheat germ agglutinin staining (scale bar=25 μm) from male mice at 3, 10, and 12 weeks after icKO and WT (10 weeks after icKO). Lipid droplets are indicated by black arrows. **H**, Gross morphological images of zebrafish at 50-hpf. The zebrafish pericardium (yellow zone) and heart (red zone) are indicated. **I**, Quantification of the pericardial area of embryos in **H** (n=10 embryos per group). *P<0.05; ***P<0.001; ****P<0.0001.

In addition, FARS2 is widely expressed and particularly enriched in the heart.^25^ Global *Fars2* knockout is lethal, indicating its indispensability for developmental viability.^25^ Therefore, we engineered a heart-specific *Fars2* knockout mouse model (inducible conditional knockout [icKO]; Figure 2A; Figure S7A and S7B). We confirmed a complete loss of FARS2 in the hearts of mice 2 weeks after icKO (Figure 2B). The mice had a reduced body weight from 8 to 9 weeks after icKO (Figure 2C; Figure S7C), largely attributable to lower food intake and physical inactivity (Video 1). In addition, the life span of icKO mice was remarkably shortened, with sudden death occurring from ≈11 to ≈13 weeks for male mice and ≈11.5 to ≈13.5 weeks for female mice after icKO (Figure 2D). Ventricular rupture was not detected in the hearts of mice that experienced sudden death (Figure S7D).

We next explored the effects of FARS2 ablation on cardiac morphology at different times. Compared with WT, the ratio of heart weight to tibia length in icKO male mice was increased by 7.1% (no change in female mice) after 3 weeks, 47.6% (41.3% in female mice) after 10 weeks, and 72.8% (69.6% in female mice) after 12 weeks (Figure 2E); the ratio of heart weight to body weight showed a similar tendency as ratio of heart weight to tibia length (Figure S7E). With the extension of knockout time, the icKO hearts presented progressive enlargement, eccentric hypertrophy, HF, and sudden death (Figure 2G). Clear signs of heart degeneration were demonstrated by the notably increased expression of hypertrophy markers, including *Nppa*, *Nppb*, and *Myh7*, at different times after icKO (Figure 2F). The increased ratio of lung weight to body weight also indicated HF (Figure S7F). Moreover, the myocardium of 10-week-old and surviving 12-week-old icKO mice exhibited cardiomyocyte enlargement, slight fibrosis, and lipid droplets accumulation (Figure 2G). The hearts of *cMut/−* mice also showed slight fibrosis, lipid droplet accumulation, and tendency of left ventricular wall thickening (Figure S4F and S4G). All these findings were consistent with the pathological results of the myocardium samples from patients (Figure S8C and S8D).

Furthermore, we generated a global *fars2*-knockdown zebrafish with 2 *fars2*-targeted morpholinos (Figure S7G). The loss of *fars2* led to delayed growth, pericardial edema, reduced contractile force, and precardial blood congestion, manifesting HF (Figure 2H and 2I; Videos 2 through 4).^26^ These findings suggest that ablation of FARS2 caused cardiomyocyte hypertrophy, fibrosis, and HF, eventually leading to sudden death, regardless of sex.

### FARS2 Deficiency Causes Myocardial and Mitochondrial Dysfunction in Vivo and in Vitro

**Figure 3.**
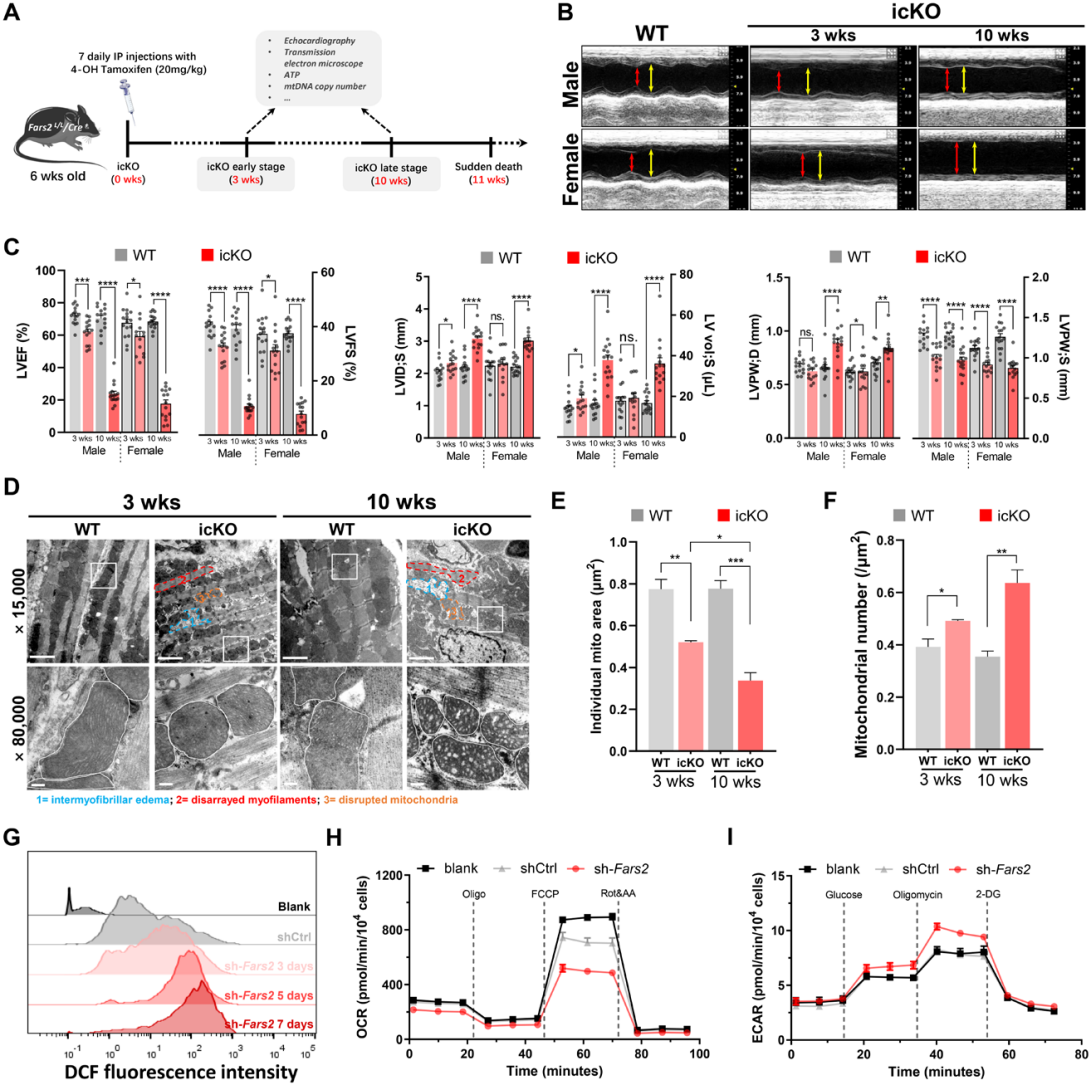
**Defective myocardial and mitochondrial functions in Fars2 deficiency models. A**, Schematic diagram of experimental protocol in mice. **B**, Representative M-mode echocardiographic images from the mice 3 and 10 weeks after inducible cardiac-specific *Fars2* knockout (icKO). End-systole stages are indicated by red lines and end-diastole stages by yellow lines. **C**, Echocardiographic quantifications of wild-type (WT) and icKO mice. Shown in the statistical graph are left ventricular ejection fraction (LVEF) and left ventricular fractional shortening (LVFS; left panel), left ventricular systolic internal diameters (LVID;S) and left ventricular volume at end-systole (LV vol;S; **middle** panel), and left ventricular posterior wall thickness at end-diastole (LVPW;D) and left ventricular posterior wall thickness at end-systole (LVPW;S; **right** panel). **D**, Mitochondrial and myocardial dysmorphometry evoked by FARS2 (mitochondrial phenylalanyl-tRNA synthetase) deficiency. Transmission electron microscopy images (×15 000 and ×80 000); representative cardiomyocyte mitochondria are emphasized by white lines. A representative intermyofibrillar edema area is indicated by blue lines, a representative disarrayed myofilaments area by red lines, and a representative disrupted mitochondria area by orange lines. **Top** panel scale bar=2 μm; **bottom** panel scale bar=200 nm. **E**, Quantification of the average individual mitochondrial area (μm^2^) from **D** (n=3). **F**, Quantification of the average mitochondrial number (μm^2^) from **D** (n=3). **G**, Increase of reactive oxygen species production in *Fars2* knockdown neonatal rat ventricular myocytes at 3, 5, and 7 days by DCFH-DA staining. **H**, Oxygen consumption rate in neonatal rat ventricular myocytes infected with shCtrl or sh-*Fars2* at 3 days. **I**, Extracellular acidification rate in neonatal rat ventricular myocytes infected with shCtrl or sh-*Fars2* at 3 days.

To investigate the mechanisms underlying the cardiac hypertrophy induced by FARS2 ablation, we next examined the myocardial and mitochondrial functions in the early and late stages (3 and 10 weeks after icKO, respectively) in icKO and WT control littermates (Figure 3A). Unlike the changes in cardiac morphology, echocardiography showed that the left ventricular ejection fraction and left ventricular fractional shortening were diminished after 3 weeks and worse after 10 weeks, indicating that the myocardial function had already been impaired at the early stage (Figure 3B). The left ventricular end-systolic internal diameters, volume, and end-diastole posterior wall were increased, whereas the thickness of the left ventricular end-systolic posterior wall was decreased both 3 and 10 weeks after the establishment of the icKO (Figure 3C; Figure S9A and S9B). *cMut/−* mice also showed impaired left ventricular ejection fraction and left ventricular fractional shortening (Figure S4H). Compared with controls, *fars2* morphants showed a slower heartbeat and abnormal circulation in the common cardinal vein (Videos 2 through 4).

Transmission electron microscopy studies showed microstructural damage in patient myocardium samples as intermyofibrillar edema, disarrayed myofilaments, and smaller mitochondria with honeycomb cristae (Figure S8E). Similar microstructural damage was observed in icKO mice (Figure 3D). The cross-sectional area of individual mitochondria decreased and mitochondrial number increased in both stages in icKO mice and patients (Figure 3E and 3F; Figure S8E). Moreover, the decreased ATP level and increased reactive oxygen species (ROS) production further demonstrated that FARS2 deficiency impaired mitochondrial bioenergetics and ROS homeostasis (Figure S9B through S9D).

In addition, a *Fars2* knockdown cell model was established using adenovirus-mediated short hairpin RNA (Ad-sh-*Fars2*) in neonatal rat primary myocardial cells (neonatal rat ventricular cardiomyocytes [NRVMs]; Figure S10A through S10C). Consistent with the observations in icKO mice, FARS2 deficiency caused notable mitochondrial dysfunction, including decreased ATP level, decreased mitochondrial membrane potential (ΔΨm), and increased ROS production in NRVMs (Figure 3G; Figure S10D through S10G). The decreased ΔΨm and increased ROS production were confirmed by staining with tetramethylrhodamine ethyl ester and Mito-SOX (Figure S11). The homeostasis of the nicotinamide adenine dinucleotide (NAD) pool is essential for mitochondrial redox capacity. The deficiency of FARS2 resulted in a significant decrease in NAD^+^ and an increase in NADH. Although total NAD level had no changes, FARS2 deficiency resulted in a significant reduction in the NAD^+^/NADH ratio (Figure S10H). Moreover, to directly assess mitochondrial respiration, we measured the oxygen consumption rate (OCR) in *Fars2*-deficient NRVMs. Under basal conditions, the OCR decreased, indicating impaired basal respiration in sh-*Fars2*. Upon addition of oligomycin (an ATP synthase inhibitor), the OCR dropped in sh-*Fars2*, indicating that the basal ATP production rate and proton leak decreased. To determine maximal respiration, FCCP, a potent uncoupler of OXPHOS, was added and the stimulated increase in OCR was blunted in sh-*Fars2*. Antimycin A and rotenone, electron transport chain inhibitors, were finally added to assess spare respiratory capacity, and the OCR was also decreased in sh-*Fars2* (Figure 3H; Figure S10I). We further assessed glycolysis capacity in NRVMs by measuring the extracellular acidification rate. The deficiency of FARS2 slightly enhanced the glycolytic capacity and glycolysis level in NRVMs (Figure 3I; Figure S10J). These results demonstrate that FARS2 was essential for myocardial and mitochondrial functions, especially in maintaining mitochondrial bioenergetics, redox capacity, and ROS homeostasis.

### FARS2 Deficiency Impairs Myocardial Metabolism and Mitochondrial Homeostasis

**Figure 4.**
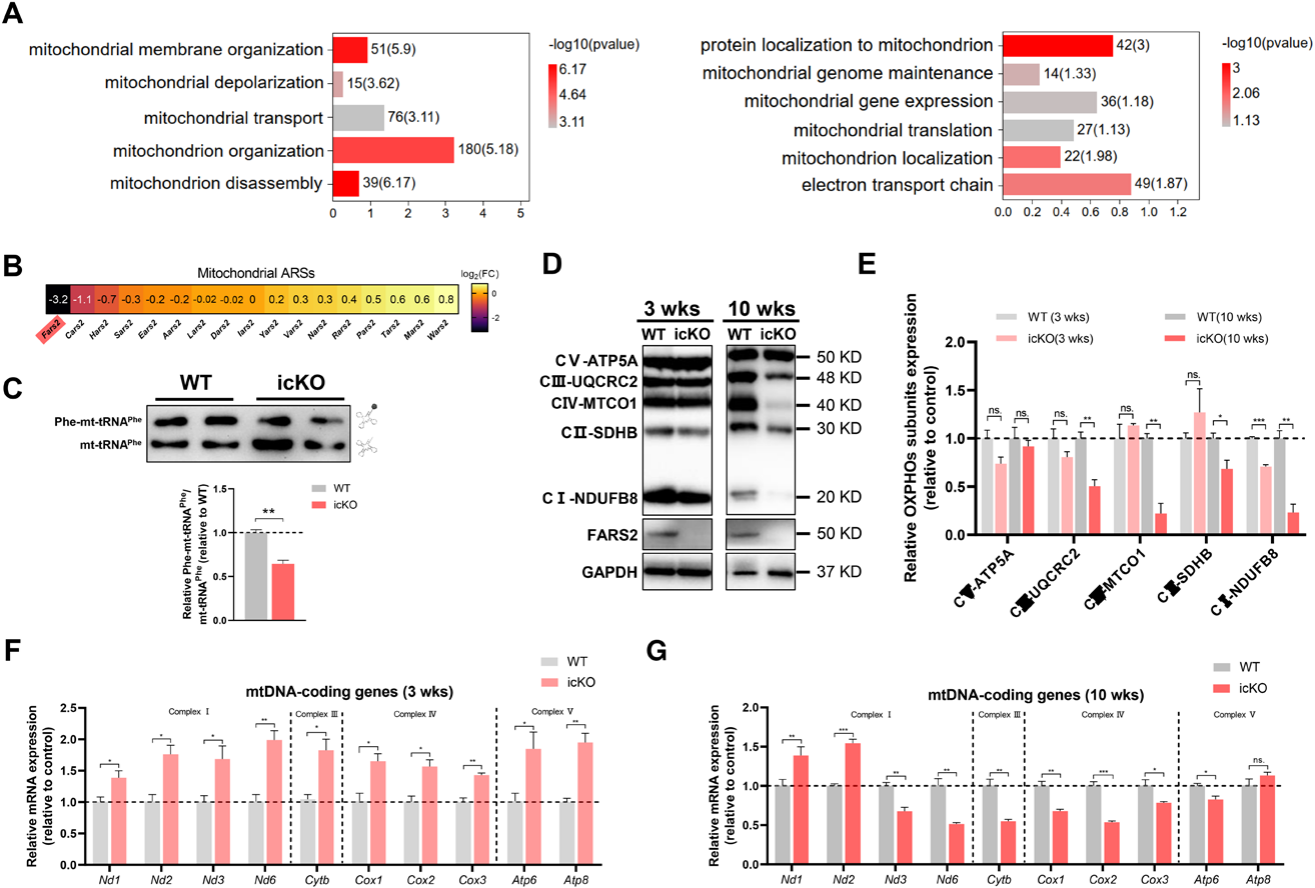
**Defective mitochondrial homeostasis and protein synthesis in icKO hearts. A**, Gene Ontology analysis of mitochondrial homeostasis–related biology process changes. **B**, Heatmap of mitochondrial aminoacyl-tRNA synthetase (mtARS) genes of inducible cardiac-specific *Fars2* knockout (icKO) compared with wild-type (WT) mice from RNA sequencing analysis. **C**, **Top**, aminoacylation assay for mitochondrial mt-tRNA^Phe^ in mouse heart. Charged (Phe-mt-tRNA^Phe^) and uncharged tRNA (mt-tRNA^Phe^) are indicated. **Bottom**, quantification of Phe-mt-tRNA^Phe^/mt-tRNA^Phe^ from the **top** panel (n=3). **D**, Western blots of oxidative phosphorylation (OXPHOS) complexes and *Fars2* in WT and icKO mice. **E**, Quantification of the OXPHOS complexes from **D** (n=3). **F** and **G**, Expression levels of mitochondrial DNA (mtDNA) coding genes in the heart of icKO mice at 3 weeks (**F**) and icKO mice at 10 weeks (**G**; n=3 mice per group). *P<0.05; **P<0.01; ***P<0.001.

To further explore the potential mechanisms underlying mitochondrial dysfunction caused by FARS2 deficiency, we analyzed the transcriptome of the heart in icKO and WT mice at the late stage using bulk RNA sequencing. Among 16 453 genes analyzed, 2918 (17.74%) were upregulated and 2886 (17.54%) were downregulated (Figure S12A and S12B). Kyoto Encyclopedia of Genes and Genomes enrichment analysis revealed that cellular metabolic pathways including amino acid biosynthesis, glutathione, carbon, pyruvate, and fatty acid metabolism were significantly affected, thus explaining the bioenergetic defects in icKO hearts and patients with HCM (Figure S12C). The differentially expressed genes were also enriched in cardiac muscle contraction and hypertrophic cardiomyopathy, in good agreement with the cardiac morphology of FARS2-deficient models. Gene Ontology enrichment analysis of biological processes demonstrated that processes related to mitochondrial homeostasis, such as mitochondrion organization, disassembly, and electron transport chain, were severely impaired in icKO mice (Figure 4A). Because of the crucial role of ARS in mt-tRNA metabolism and mtDNA-encoded protein synthesis, we investigated whether there are compensatory changes in other aminoacyl-tRNA synthetases caused by FARS2 deficiency. Except for FARS2, the transcript levels of other mtARS showed no significant changes (Figure 4B). FARS2 deficiency decreased the aminoacylation of mt-tRNA^Phe^ (Figure 4C).

Given the essential role of OXPHOS in bioenergetics and ROS production, we next investigated whether FARS2 deficiency damaged the assembly and function of OXPHOS complexes. mtDNA-encoded protein expression and the levels of OXPHOS complexes were greatly diminished in the late stage of icKO (Figure 4D and 4E; Figure S13A and S13B). FARS2 deficiency also decreased the OXPHOS complex levels in NRVMs (Figure S13C and S13D). The activities of OXPHOS complexes were significantly damaged in the late stage of icKO (Figure S13E). Inconsistent with the changes in cardiac function, there were no obvious changes in OXPHOS complex levels in the early stage of icKO (Figure 4D and 4E; Figure S13A and S13B). We further determined whether this consequence was attributable to the compensation of the transcripts in mitochondrion-encoded genes. As we expected, the transcript levels of the mitochondrion-encoded genes increased in the early stage of icKO, and decreased in the late stage of icKO (Figure 4F and 4G). In addition, mtDNA copy number (mtDNA-CN), an essential measure for mitochondrial homeostasis, was reduced in the early stage of icKO but significantly increased in the late stage of icKO (Figure S13F). However, in NRVMs, FARS2 deficiency caused a continuous decline in mtDNA-CN (Figure S13G).

These results indicate that mitochondrial dysfunction caused by FARS2 ablation in the heart was mainly caused by impaired mitochondrial homeostasis triggered by blocking the aminoacylation of mt-tRNA^Phe^, reflected in the disruption of the mtDNA-CN, mtDNA-encoded protein expression, and further disrupted the assembly of OXPHOS complexes.

### Mitochondrial Dyshomeostasis Triggered by FARS2 Deficiency Results in MQC System Disruption

**Figure 5.**
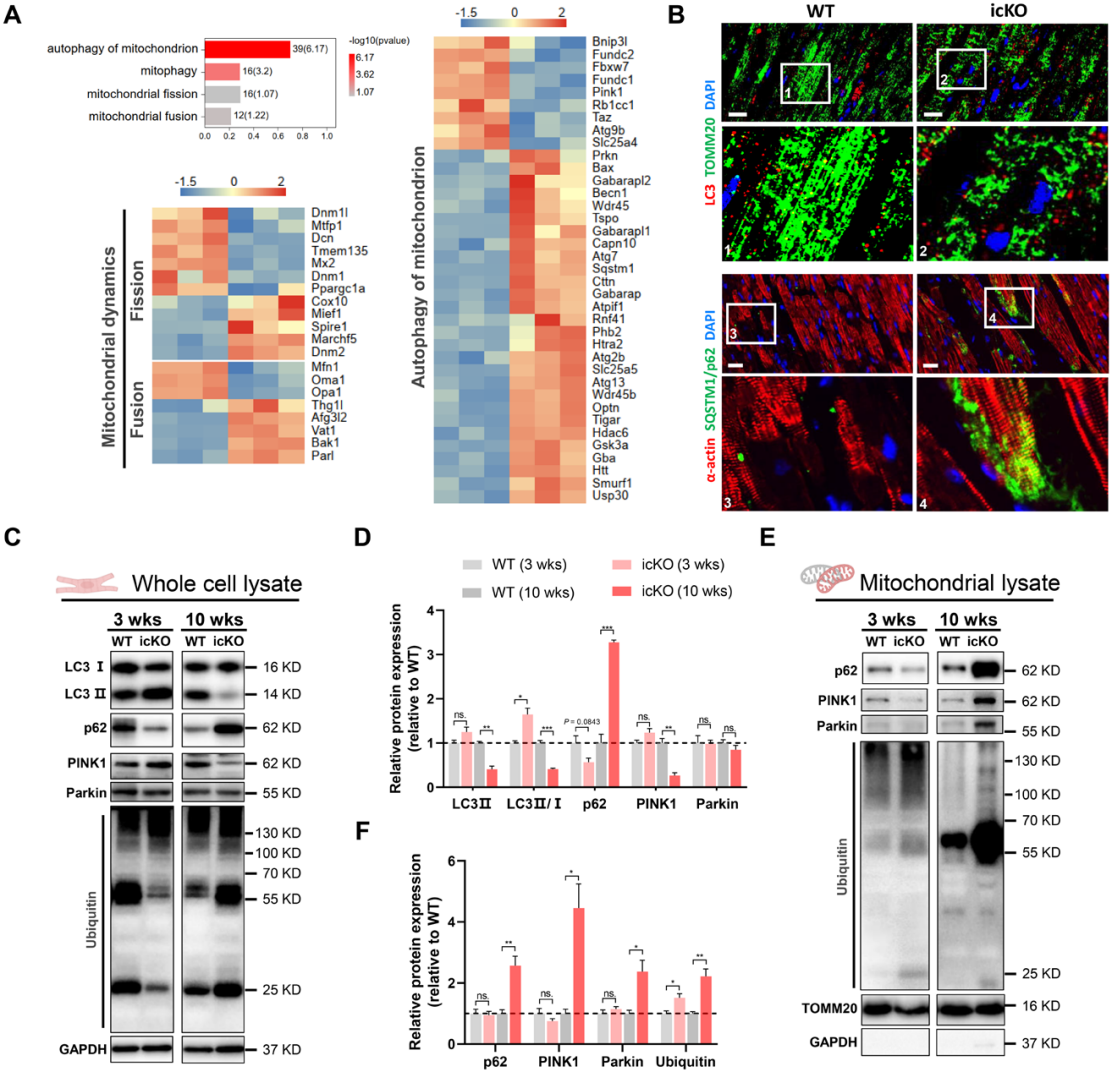
**FARS2 deficiency causes MQC system disruption. A**, Gene Ontology analysis of mitochondrial quality control (MQC) system–related biology process changes and heatmap of key genes in the autophagy of mitochondrion and mitochondrial dynamics pathways from RNA sequencing analysis. **B**, **Top**, Representative immunofluorescence images of LC3 (red) and TOMM20 (green) from mouse heart 10 weeks after inducible cardiac-specific *Fars2* knockout (icKO) or in wild-type (WT) mice. **Bottom**, Representative immunofluorescence images of α-actin (red) and SQSTM1/p62 (green) from mouse heart 10 weeks after icKO or in WT mice. Scale bar=20 μm. **C**, Western blots of proteins in autophagy of mitochondria in whole-cell lysate of WT and icKO mice. **D**, Quantification of relative protein expression in **C** (n=3). **E**, Western blots of proteins in autophagy of mitochondria in mitochondrial lysate of WT and icKO mice. **F**, Quantification of relative protein expression in **E** (n=3). *P<0.05; **P<0.01; ***P<0.001.

Given the pivotal role of the MQC system, particularly mitochondrion-related autophagy and dynamics, in the maintenance of mitochondrial homeostasis,^27,28^ we next sought to investigate the changes in these pathways at both the whole cardiomyocyte and isolated mitochondrion levels. Gene Ontology analysis of biological processes demonstrated that mitochondrial dynamics and mitochondrion-associated autophagy were altered after icKO. The key molecules of these pathways were widely affected in the late stage of icKO, which was further verified using quantitative reverse transcription polymerase chain reaction (Figure 5A; Figure S14A). Then, we analyzed the changes in mitochondrial dynamics in the 2 stages of icKO. At the whole cardiomyocyte level, the expression of DRP1 (dynamin-related protein 1) and DRP^S616^ increased, and that of DRP1^S637^, MFN1 (mitofusin 1), and MFN2 (mitofusin 2) decreased (Figure S14B and S14C). The abundant recruitment of DRP1 and the decrease in MFN1 and OPA1 (optic atrophy protein 1) expression in isolated mitochondria suggested that the mitochondria were fragmented, which was consistent with the transmission electron microscopy results (Figure 3D through 3F; Figure S14D and S14E). In addition, immunofluorescence demonstrated the hyperfragmentation of mitochondria in icKO (Figure 5B). Similar changes were also observed in patient tissues (Figure S8E) and FARS2-deficient NRVMs (Figure S11; Figure S15H).

We further detected the autophagy flow changes in the mitochondria of icKO mice.^29^ In the early stage of icKO, the increased LC3-II/LC3-I (microtubule-associated protein 1 light chain 3) ratio, the decreased p62 expression and ubiquitin-labeled protein levels in whole cardiomyocytes, and the increased ubiquitinated mitochondrial proteins indicated the activation of mitochondrial autophagy (Figure 5C through 5F). However, we observed the suppression of LC3-II/LC3-I conversion, inhibition of PINK1 (PTEN-induced putative kinase 1) expression, and accumulation of SQSTM1/p62, suggesting inhibition of mitophagy in the late stage of icKO (Figure 5B through 5D). This process had no effects on the recruitment of PINK1 and Parkin to the mitochondria (Figure 5E and 5F). Recruitment of Parkin to the mitochondria and accumulation of SQSTM1/p62 were detected in tissues from patients (Figure S8F and S8G). Furthermore, to ascertain whether NRVMs showed similar changes in autophagy flow with FARS2 deficiency, we examined the LC3-II/LC3-I ratio at the time of *Fars2* knockdown. However, FARS2 deficiency only led to a continuous increase in autophagy flow in NRVMs, as verified by treatment with bafilomycin A1 (Figure S15A through S15E).^30^ To confirm this observation and investigate the fate of autophagolysosomes in the NRVMs, mGFP-RFP-LC3 adenovirus transfection was performed. As shown in Figure S15F and S15G, autophagosomes and autolysosomes were abundant in *Fars2*-knockdown NRVMs and there were almost no autophagosomes in the control group. These results suggested that NRVMs merely reproduced the early-stage icKO heart phenotype because of the limited period of primary myocardial culture, which could be used to investigate the causes of mitochondrial dysfunction in the early stage of icKO.

We further investigated the functional significance of the continuous increase in autophagy flow. Flow cytometry analysis showed that the mitochondrial mass stained using Mito-Tracker green decreased with the extension of *Fars2* knockdown (Figure S15I and S15J), which was supported by the decrease in mtDNA-CN both in vitro and vivo at the early stage (Figure S13F and S13G). As a consequence, the continuous increase in autophagy caused by FARS2 deficiency placed excessive stress on the MQC system and resulted in an unbalanced mitochondrial mass. The excessive decrease in mitochondrial mass may coordinate mitochondrial biogenesis through mitochondrial–nuclear communication. Therefore, we further examined mitochondrial biogenesis in both stages. Consistent with the quantitative reverse transcription polymerase chain reaction results, PGC-1α expression did not change in the early stage of icKO, whereas it decreased in the late stage, indicating that mitochondrial biogenesis did not contribute to the recovery of mitochondrial dyshomeostasis in FARS2-deficient hearts (Figure S14F and S14G). Taken together, these results demonstrate that mitochondrial dyshomeostasis triggered by FARS2 deficiency resulted in MQC system disruption.

### Mitochondrial Fission and Autophagy Inhibition Attenuate Mitochondrial Dyshomeostasis Induced by FARS2 Deficiency in Vitro

**Figure 6.**
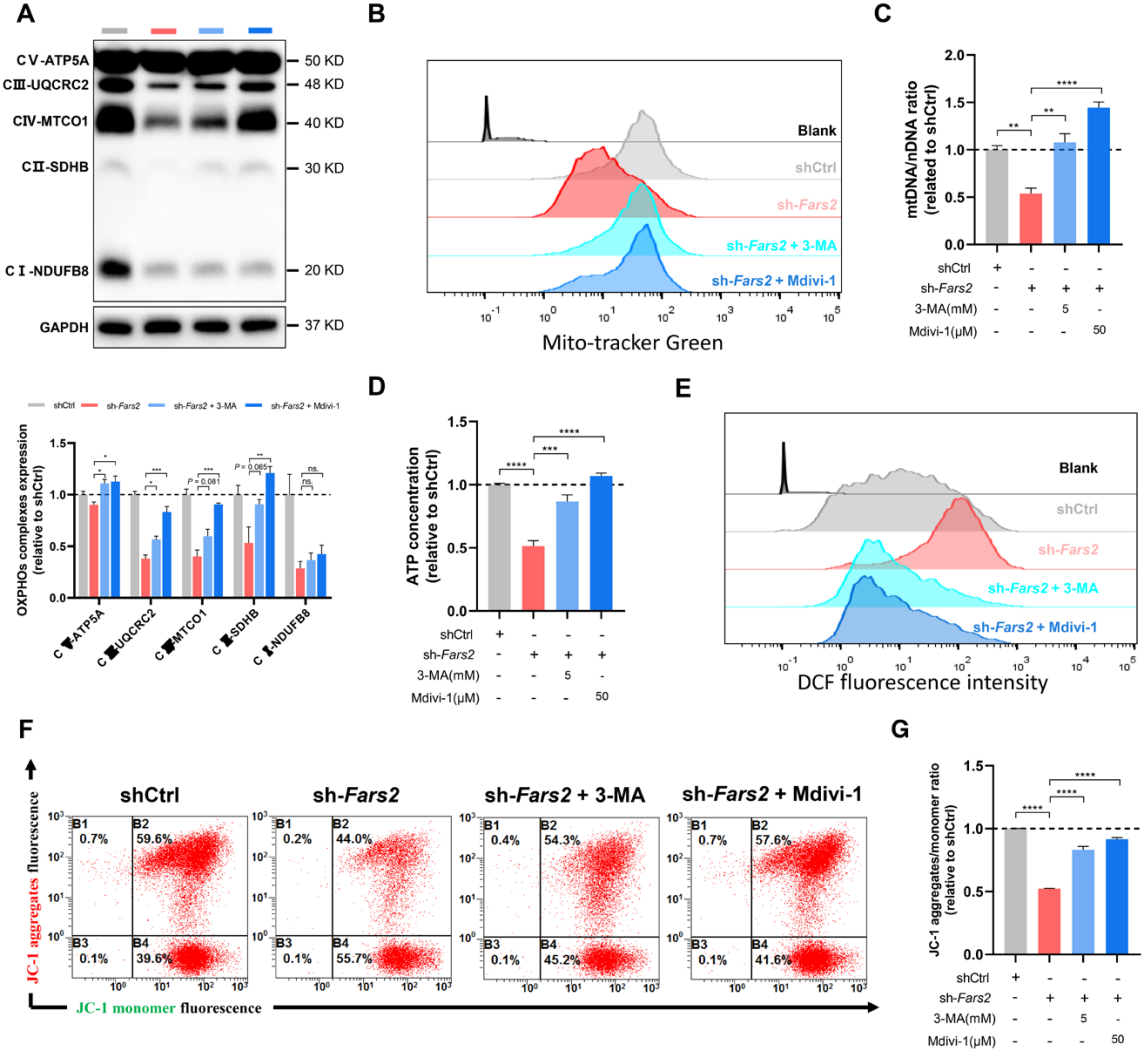
**3-MA and Mdivi-1 attenuate mitochondrial dyshomeostasis induced by FARS2 deficiency in neonatal rat ventricular myocytes. A**, **Top**, Western blots of oxidative phosphorylation (OXPHOS) complexes of control and *Fars2* knockdown neonatal rat ventricular myocytes (NRVMs) after 3-methyladenine (3-MA) or Mdivi-1 treatment. **Bottom**, Quantification of the OXPHOS complexes from the **top** panel (n=3). **B**, Increased mitochondrial mass in *Fars2* knockdown NRVMs after 3-MA or Mdivi-1 treatment by mitoTracker Green staining. **C**, Mitochondrial DNA copy number (mtDNA-CN; mtDNA/nuclear DNA) was detected by quantitative reverse transcription polymerase chain reaction of NRVMs after 3-MA or Mdivi-1 treatment (n=3). **D**, Quantification of the relative ATP contents of *Fars2* knockdown NRVMs after 3-MA or Mdivi-1 treatment (n=3). **E**, Reactive oxygen species (ROS) production in *Fars2* knockdown NRVMs at 3, 5, and 7 days after 3-MA or Mdivi-1 treatment by DCFH-DA staining. **F**, Representative FARS2 (mitochondrial phenylalanyl-tRNA synthetase) images in *Fars2* knockdown NRVMs after 3-MA or Mdivi-1 treatment by JC-1 staining. **G**, Quantification of relative JC-1 aggregates/monomer ratio from **F** (n=3). *P<0.05; **P<0.01; ***P<0.001; ****P<0.0001.

To further investigate whether mitochondrial dyshomeostasis caused by FARS2 deficiency can be alleviated by intervening in the MQC system, Mdivi-1 or 3-methyladenine was used to inhibit mitochondrial fission or autophagy in vitro, respectively (Figure S16A). 3-Methyladenine treatment significantly suppressed the LC3-II/LC3-I conversion and the localization of LC3 to the mitochondria, with no effect on the recruitment of DRP1. Different from 3-methyladenine, Mdivi-1 both reduced the recruitment of DRP1 and LC3 to the mitochondria and suppressed the LC3-II/LC3-I conversion. Between the 2 inhibitors, Mdivi-1 more notably reversed mitochondrial fragmentation caused by FARS2 defects (Figure S16B through S16G). Moreover, the inhibitors substantially restored the relative content of complexes II, III, and IV, especially complex III, which was recognized as the major source of ROS production in addition to complex I (Figure 6A). Flow cytometry revealed that both inhibitor treatments significantly recovered the decrease in mitochondrial mass caused by FARS2 deficiency, which was further confirmed by mtDNA-CN (Figure 6B and 6C; Figure S16H). Compared with sh-*Fars2* treatment, both inhibitor treatments increased ATP and decreased the production of ROS in NRVMs (Figure 6D and 6E; Figure S16I). Compared with the sh-*Fars2* treatment, these inhibitors partially rescued the diminished ΔΨm caused by FARS2 deficiency (Figure 6F and 6G). All these results demonstrate that mitochondrial fission and autophagy inhibition attenuated mitochondrial dyshomeostasis induced by FARS2 deficiency in vitro.

### AAV9-Mediated Mitochondrial Dynamics Intervention Attenuates Myocardial Dysfunction Induced by FARS2 Deficiency and Prolongs the Life Span in Mice

**Figure 7.**
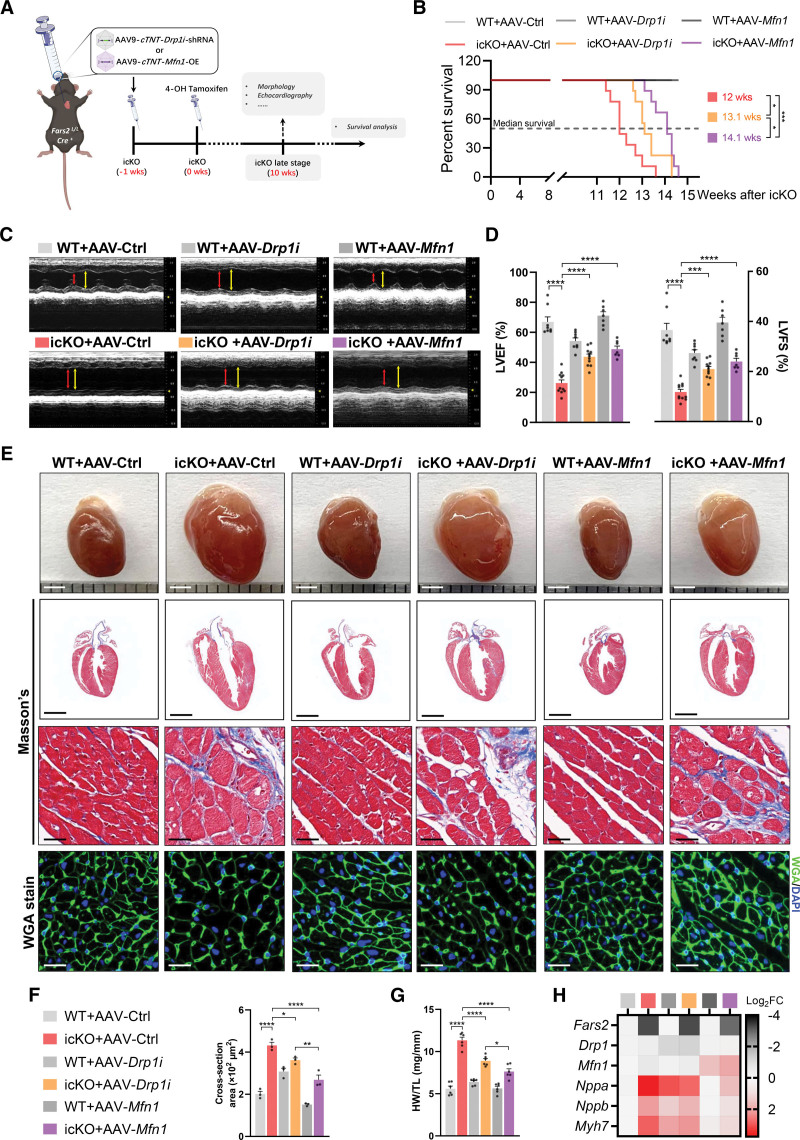
**AAV9-mediated *Drp1* knockdown or *Mfn1* overexpression attenuates myocardial dysfunction induced by *Fars2* deficiency and prolongs life span in mice. A**, Schematic diagram of experimental protocol of adeno-associated virus 9 (AAV9)–mediated rescue tactics for inducible cardiac-specific *Fars2* knockout (icKO) mice. **B**, Kaplan-Meier survival curves for wild-type (WT) and icKO mice after AAV9 treatment (n =9 per group). The median survival times for each group are marked. **C**, M-mode echocardiographic images from each group of mice at 10 weeks after icKO. End-systole stages are indicated by red lines and end-diastole stages by yellow lines. **D**, Left ventricular ejection fraction (LVEF; **left** panel) and left ventricular fractional shortening (LVFS; **right** panel; n=7–11 mice per group). **E**, Representative hearts (scale bar=2 mm): longitudinal sections (scale bar=2 mm), Masson trichrome staining (scale bar=25 μm), hematoxylin & eosin staining (scale bar=25 μm), and wheat germ agglutinin (WGA) staining (scale bar=25 μm) from each group 10 weeks after icKO. **F**, Quantification of average cardiomyocyte sectional size from **E** (at least 100 cells from 3 mice per group). **G**, Ratios of heart weight to tibia length (HW/TL; mg/mm) in different groups (n=6). **H**, Relative mRNA levels of *Fars2*, *Drp1*, *Mfn1*, and cardiac hypertrophy markers (*Nppa*, *Nppb*, and *Myh7*) in mice 10 weeks after icKO (n=3). *P<0.05; **P<0.01; ***P<0.001; ****P<0.0001.

Having found that FARS2 deficiency could disrupt the MQC system by increasing mitochondrial fission and decreasing fusion, we investigated whether altering mitochondrial dynamics using adeno-associated virus 9 (AAV9) could exert protective effects on cardiac hypertrophy and provide potential clinical benefits (Figure 7A; Figure S17A). First, we assessed the effects of AAV9-mediated *Drp1* knockdown (AAV-*Drp1i*) and *Mfn1* overexpression (AAV-*Mfn1*) on FARS2 deficiency–induced life span shortening in icKO mice. Both AAV-*Drp1i* and AAV-*Mfn1* treatment significantly prolonged the life span of icKO mice, with median survival times of ≈13.1 weeks (≈1 week longer than AAV-Ctrl) for AAV-*Drp1i* and ≈14.1 weeks for AAV-*Mfn1* (≈2 weeks longer than AAV-Ctrl) after icKO (Figure 7B).

To explore the effects of interfering with mitochondrial dynamics on icKO-induced cardiac hypertrophy, the morphology and function of hearts treated with AAV-*Drp1i* and AAV-*Mfn1* or the controls were further investigated (10 weeks after icKO; Figure 7A and 7B). Echocardiographic analysis revealed that AAV-*Drp1i* and AAV-*Mfn1* treatment attenuated the left ventricular ejection fraction and left ventricular fractional shortening impairment after FARS2 deficiency (Figure 7C and 7D). FARS2 deficiency increased the cardiomyocyte cross-sectional area and cardiac hypertrophy, whereas AAV-*Drp1i* and AAV-*Mfn1* mitigated these changes (Figure 7E and 7F). However, there were no notable changes in collagen deposition upon treatment with AAV-*Drp1i* and AAV-*Mfn1* (Figure 7E). In line with attenuated cardiac hypertrophy, AAV-*Drp1i* and AAV-*Mfn1* treatment reduced the ratio of heart weight to tibia length, heart weight to body weight, and hypertrophy markers expression 10 weeks after icKO (Figure 7G and 7H; Figure S17B).

Overall, these findings suggest that AAV9-mediated mitochondrial dynamics intervention could elicit functional and morphological benefits in FARS2 deficiency–related cardiac hypertrophy, highlighting the pathological function of disrupted mitochondrial dynamics and potential therapeutic tactics in cardiac hypertrophy caused by FARS2 deficiency.

## DISCUSSION

**Figure 8.**
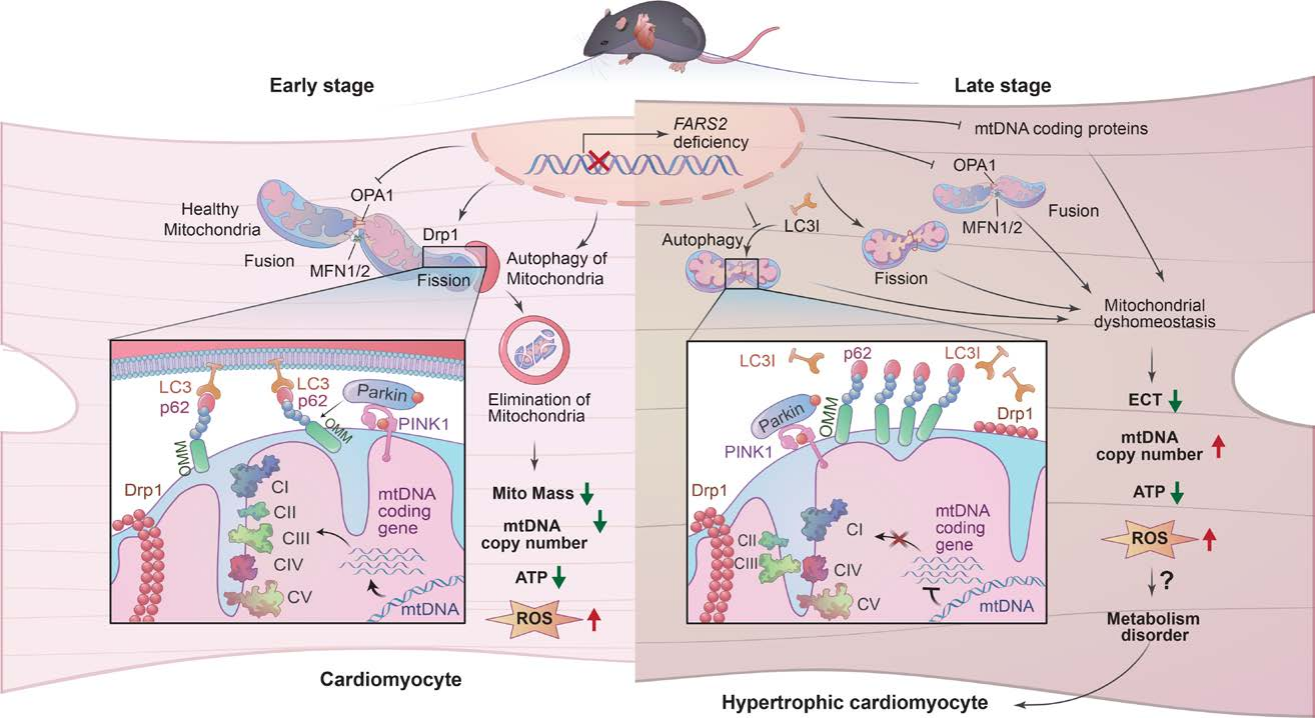
**FARS2 deficiency causes cardiomyopathy by disrupting mitochondrial homeostasis and the mitochondrial quality control system.** ETC indicates electron transport chain; FARS2, mitochondrial phenylalanyl-tRNA synthetase. mtDNA, mitochondrial DNA; and ROS, reactive oxygen species.

We report that *FARS2* is a potential pathogenic gene related to cardiomyopathy. We identified 7 novel variants of *FARS2* in patients with HCM and determined that these variants caused FARS2 deficiency. FARS2 deficiency triggered mitochondrial dyshomeostasis in murine hearts by impairing the expression of mtDNA-encoded proteins, driving dysregulation of mitochondrial dynamics and autophagy, ultimately resulting in impaired mitochondrial and myocardial function. In addition, we confirmed that inhibition of mitochondrial-associated autophagy or mitochondrial dynamics could provide functional and morphological benefits in FARS2-related cardiac hypertrophy by restoring mitochondrial dyshomeostasis. As a consequence, our findings provide new insights into the molecular diagnosis and prevention of heritable cardiomyopathy, as well as therapeutic options for FARS2-associated cardiomyopathy (summarized in Figure 8).

Some variants involved in mitochondrial translation machinery genes recently have been reported to cause myocardial hypertrophy.^31^ For instance, deficiency of *GTPBP3* or *MTO1* causes myocardial hypertrophy by disrupting mitochondrial biogenesis and RNA maturation in zebrafish.^32,33^ The variant of the mitochondrial 16S ribosomal RNA gene (*MT-RNR2*) results in mitochondrial dysfunctions and ultrastructure defects, causing HCM.^34^ Several variants of mtARS (eg, *AARS2*, *VARS2*, and *YARS2*) have been identified to cause HCM in humans by mitochondrial deficiency. In murine studies, heart-specific loss of *Dars2* causes cardiac hypertrophy.^35^ Chemical random mutagenesis in *WARS2*^*V117L*^ causes sensorineural hearing loss and HCM in mice.^36^ However, studies of *DARS2* and *WARS2* showed no direct clinical evidence for humans.^11,13^ Here, we demonstrated that another member of mtARS, *FARS2*, is a novel pathogenic gene in HCM. We confirmed that FARS2 deficiency directly drives cardiac hypertrophy, HF, and sudden death on the basis of mouse and zebrafish models. Further studies are required to determine the specific cause of sudden death triggered by FARS2 deficiency. In addition, all the known *FARS2* variants are reportedly responsible for various neurological disorders, but their involvement in other physiological systems, including the heart, has not been reported.^11,13^ In this study, none of the patients or their kin presented or were diagnosed with neurological diseases. To our knowledge, this study is the first to report that *FARS2* variants cause cardiomyopathy, and these variants are unrelated to the central nervous system.

Although reported mtARS variant–related cases have increased steadily, the characteristics of these pathogenic variants are unclear. Systematic characterization of pathogenic mtARS variants in vitro is a more recent development; it is hypothesized that these variants cause a reduction in tRNA aminoacylation and ATP-binding activity. Variable results have revealed that some pathogenic variants result in drastic (≈4000-fold) reductions in aminoacylation rates and ATP-binding activity, whereas others have no effect.^37^ Furthermore, aberrant mitochondrial tRNA metabolism and impaired mitochondrial localization and protein stability could be other potential pathogenic characteristics.^36,38,39^ Here, we showed that these patient-identified *FARS2* variants triggered FARS2 deficiency partially through impaired mitochondrial localization, reduced protein stability, and predictively decreased ATP-binding (or tRNA aminoacylation) activity. Heart-specific heterozygous *FARS2*^*R415L*^ or *FARS2* icKO mice were established for further morphological and functional studies. However, the pathogenicity of the patient-derived variants should be further evaluated by measuring the tRNA aminoacylation, ATP-binding activity in vitro and mitochondrial tRNA metabolism in vivo.

We have shown that mitochondrial functional homeostasis was severely impaired in *FARS2*-knockout and knockdown models, indicating that FARS2 is essential for mitochondrial homeostasis. Perturbed mitochondrial metabolic signaling has been confirmed as a partial pathogenic mechanism in HCM.^10^ Mitochondrial ultrastructural and morphological alterations seen in HCM are correlated with reduced mitochondrial respiration capacity and decreased mitochondrial bioenergetics.^9,27^ In patients and animal models of HCM, there were signs of fragmented mitochondria with disrupted cristae, mtDNA depletion, and reduced levels and activity of OXPHOS complexes.^9,10,40^ Consistent with previous studies, we found fragmented mitochondria, reduced levels of OXPHOS complexes, and decreased mitochondrial bioenergetics in icKO hearts. However, some specific changes were observed in the FARS2-deficient hearts. Increased mtDNA levels were indicated in late-stage icKO hearts, which has been described in flies depleted of *fars2* or *sars2*.^41,42^ In addition, mitochondrial honeycomb cristae were observed in patients’ myocardial tissues and late-stage icKO hearts, which was consistent with the ultrastructural feature in *fars2*-knockout larvae but not consistent with *sars2*-defective larvae of *Drosophila*.^41,42^ In 4the future, it might be interesting to examine whether honeycomb cristae and increased mtDNA levels are general or tissue-specific mitochondrial features associated with mtARS variants; this could provide novel insights into the pathogenesis of different diseases caused by mtARS deficiency.

An important finding of this study is that dysregulation of the MQC system plays a crucial role in the pathological remodeling of cardiac hypertrophy and HF triggered by FARS2 deficiency. The clearance of damaged mitochondria through mitochondrial fission and autophagy is considered an adaptation of cardiomyocytes to mitochondrial dysfunction.^19–21^ In this study, RNA sequencing and further analysis of whole-cell and subcellular lysates identified various states of mitochondrial dynamics and mitochondrion-related autophagy pathways at different icKO stages. In the early stage, FARS2 deficiency activated mitochondrial fission, inhibited mitochondrial fusion, and increased autophagy flow. These changes caused a decrease in mitochondrial mass and mtDNA-CN, triggering impaired mitochondrial bioenergetics and cardiac dysfunction. The expression of OXPHOS subunits had no significant changes. Upon prolongation of FARS2 deficiency, although mitochondrial fission and autophagy were continuously activated, autophagic flow was blocked upon LC3 inhibition. Blockade of autophagy directly led to mtDNA accumulation. Moreover, the significant decrease in the number of mtDNA-coding gene transcripts was likely compatible with reduced mitochondrial translation capacity after FARS2 deficiency. These findings indicate a complex remodeling mechanism underlying the response of the myocardium to sustained mitochondrial functional stress caused by FARS2 deficiency. This remodeling is evidenced by the removal of damaged mitochondria in the early stage and retainment of as many mitochondria as possible in the late stage (Figure 8). Furthermore, mitochondrial fission or autophagy intervention through inhibitors or AAV9 could attenuate FARS2 deficiency–induced mitochondrial dyshomeostasis and myocardial dysfunction. Our results highlight the importance of the MQC system dysregulation in the pathological remodeling of cardiac hypertrophy caused by FARS2 deficiency and the potential therapeutic approaches in FARS2 or mtARS-associated cardiomyopathy.

There are several limitations that should be considered when interpreting the results. First, we identified FARS2^R415L^ as the pathogenic variant in family 1 on the basis of the cosegregation analysis and Sanger sequencing. Nonetheless, the homozygosity of FARS2^R415L^ in II-1 needs to be explained. Probe-based single nucleotide polymorphism array analysis (Methods in the Supplemental Material) excluded the possibility of loss of heterozygosity, copy number variants, or uniparental disomy of genomic large fragments. The single nucleotide polymorphism results indicate that the genomic region surrounding the variant (c.1244G>T) in II-1 is a potential homozygous region (ch6:5623153-5939520), as evidenced by its distinct single nucleotide polymorphism distribution compared with I-2, II-5, and II-7 (Figure S1C; Table S1). Because a genome sample was not available for individuals I-1 and II-1 (who are both deceased), the specific reason for this potential homozygous region cannot be definitively determined. This aspect might introduce complexity to the interpretation of our results. Nonetheless, complex structural variations or genetic miscopies (beyond the scope of Sanger sequencing and single nucleotide polymorphism array analysis) might explain this homozygosity in II-1.^43^ Second, although we demonstrated that heart-specific FARS2 deficiency and FARS2^R415L^ lead to cardiac hypertrophy and HF, additional investigations are needed to ascertain whether the other variants can manifest the HCM phenotypes. Third, our results showed that AAV9-mediated intervention in mitochondrial dynamics significantly mitigates myocardial dysfunction induced by FARS2 deficiency. Nonetheless, we cannot exclude that regulation of the upstream signals of the MQC system could represent a potent strategy for treating cardiomyopathy induced by FARS2 deficiency. We acknowledge the necessity for future research to expand upon our findings. This should involve studies using alternative models to comprehensively validate the effectiveness of targeting the MQC system in the treatment of FARS2-associated cardiomyopathy and mitochondrial translation deficiency–related HCM.

This study identified *FARS2* as a potential pathogenic gene in heritable cardiomyopathy. FARS2 deficiency results in cardiac hypertrophy by impairing mitochondrial homeostasis and disrupting the MQC system. Given the profound importance of HCM and mtARS-associated diseases, our findings not only reinforce the biological function of FARS2 in heart diseases but also provide novel insights into the potential prevention of mitochondrial translation deficiency–related HCM and therapeutic targets for FARS2-associated cardiomyopathy.

## ARTICLE INFORMATION

### Acknowledgments

The authors thank the staff of the Shaanxi Provincial Key Laboratory of Clinical Genetics for study coordination and Jielai Xia (Air Force Medical University), Wenjun Yan (Air Force Medical University, Xijing Hospital), Ying Yang (Shaanxi Institute of Pediatric Diseases, Xi’an Children’s Hospital), Ling Wang (Air Force Medical University), and Kaixiang Zhou (Air Force Medical University) for assistance and discussion.

### Sources of Funding

This study was supported by the National Natural Science Foundation of China (grants 82271893, 81671476, 82302090, 81901755, and 82071932); the Key Innovative Project in Shaanxi (grant 2021ZDLSF02-02); the Natural Science Foundation of Shaanxi Province (grant 2023-JC-YB-819); the Key Research and Development Plan in Shaanxi (grants 2019SF-059 and 2020SF-204); and the Clinical Research Funding Project of Fourth Military Medical University (grant 2021XD010).

### Disclosures

None.

### Supplemental Material

Methods

Tables S1–S7

Figures S1–S17

Videos S1–S4

References 44–49

## Supplementary Material
