# Supplementary material for: FARS2 Deficiency Causes Cardiomyopathy by Disrupting Mitochondrial Homeostasis and the Mitochondrial Quality Control System
Source: Circulation. 2024 Feb 16;149(16):1268–84. doi: 10.1161/CIRCULATIONAHA.123.064489 (PMC11017836; doi:10.1161/CIRCULATIONAHA.123.064489)
Supplement: Supplementary file 1 [file cir-149-1268-s001.pdf]

Supplemental materials

FARS2 Deficiency Causes Cardiomyopathy by Disrupting Mitochondrial Homeostasis and the Mitochondrial Quality Control System

Running Title: FARS2 deficiency causes cardiomyopathy

Expanded Methods

Human Subjects

Participants experiments were carried out with Research Ethics Board approval from the XiJing Hospital of the Fourth Military Medical University. The myocardium samples used in this study were obtained from two patients (family 1-II-3 and patient 4) and five healthy donors. Informed written consent to participate in research was obtained from patients and donors.

Animal Experiments

All animal experiments were followed the rules of the American Association for the Accreditation of Laboratory Animal Care International. All procedures were approved by the Animal Care Committee of the Fourth Military Medical University accredited by AAALAC International. All animal experiments and human studies were performed according to the international guidelines.

Echocardiography and electrocardiogram in patients

For echocardiography, transthoracic echocardiography was performed using the EPIQ 7C Ultrasound System (Philips Medical Systems) with a S5-1 and X5-1 transducer (1.0 to 5.0 MHz). Measurements of key parameters were obtained, according to the recommendations of the American Society of Echocardiography.[42] For electrocardiogram (ECG), a standard 12-lead electrocardiogram (ECG-1250P; Nihon Kohden) and 24-hour Holter (DMS300-4A; DMSoftware) were used.

Genetic analysis pipeline and co-segregation analysis for family 1

Whole-exome sequencing for family 1 was accomplished by ANNGEEN Technology Co.Ltd.

High quality variants were filtered through sequencing depth (>30), Alter_ratio (>20%). Rare variants were filtered using Minor Allele Frequency (MAF) < 0.1% in 1000 Genomes and gnomAD. Then, the potential pathogenic variants were predicted by SIFT, PolyPhen_2, REVEL_score (⩾0.644), and CADD score (>25).[44] The disease-unrelated variants were excluded after comparison with healthy relatives. So far, two variants were screened as candidate pathogenic variants. However, *ADA*: c.872C>T was not eligible for co-segregation analysis (Figure S1B). Therefore, the *FARS2* variant (c.1244G>T) was preliminarily considered as the pathogenic variant for family 1.

Co-segregation analysis was performed by Sanger sequencing from patients and healthy relatives in family 1. Genomic DNA was purified from peripheral blood samples according to the QIAamp DNA Mini Kit (Qiagen, Cat# 51306). DNA were amplified by PCR (40 cycles) using high fidelity Taq polymerase (Sangon Biotech). The specific primer sequences are summarized in Table S7. Sanger sequencing was accomplished by Tsingke Biotechnology and the results were analyzed by SnapGene®4.1.9. To detect the presence of underlying genomic structural variants in family 1-Ⅱ-1 to explain the homozygosity of FARS2^{R415L}, the Single Nucleotide Polymorphism array (SNP-array) was conducted using CytoScan™ 750K (ThermoFisher, Cat# 901859) following the manufacturer's instructions. The results of the SNP-array analysis were processed using ChAS (ThermoFisher, version 4.3) following the specific limit of detection (LOD): loss of heterozygosity (LOD: probe number≥50, length≥200 Kb), copy number variation (LOD: probe number≥50, length≥400 Kb), or uniparental disomy (LOD: length≥10 Mb). The graphical representation was generated using GraphPad Prism software (version 9.0.0) (Figure S1C, Table S1).

Analysis of *FARS2* variants through ACMG/AMP guidelines [24]

For each screened variant, we summarized the MAF in population databases including 1000 Genomes (1000G_2015aug), the GnomAD, and ESP6500I-V2. Then, we calculated OR for case-control associations studies based on GnomAD (East Asian) and ChinaMAP. The variant pathogenicity was evaluated by the site conservation, splice consensus and deleterious effects on encoded amino acids.

(1) MAF of *FARS2* variants (Table S2)

The MAF for each identified variant in total or East Asian-matched population was extracted by gnomAD, ChinaMAP,[45] TOPMed Bravo, GME Variome, 1000 Genomes, ESP 6500, 4.7KJPN, and GenomeAsia.

(2) Algorithms predicting pathogenicity of *FARS2* variants (Table S2)

To summary the pathogenicity predictions of variants, the following databases were used (Table S2): PROVEAN (https://www.jcvi.org/research/provean), CADD (https://cadd.gs.washington.edu/snv), VarSite (https://www.ebi.ac.uk/thornton-srv/databases/cgi-bin/VarSite), and Franklin (https://franklin.genoox.com/) (Table S2). The conservation of FARS2 across 150 species was analyzed using the ConSurfServer (https://consurf.tau.ac.il/). The multiple sequence alignments were analyzed by the EMBL-EBI search and sequence analysis tools APIs in 2019. The variant having impact on site conservation/splicing/encoded amino acids function was predicted be harmful according to "PP3" evidence of ACMG-AMP guidelines.

(3) Case-Control Association Studies (Table S3)

The frequency of *FARS2* variants between the population with HCM and the controls

from East Asian groups of GnomAD database and the ChinaMAP [45] was compared using two-sided Fisher's exact test. OR= 1.0 indicated that the variants had no effect on the risk of the disease. OR> 1.0 indicated the variants increased the risk of the disease.

Structural model and molecular mimicking analysis of wild-type and variants of *FARS2*

The wild-type molecular structure of human *FARS2* (Figure 1D) was predicted using the AlphaFold Protein Structure Database (https://alphafold.com/). The variants of *FARS2* (Figure 1D) were predicted using Missense 3D (http://missense3d.bc.ic.ac.uk/~missense3d/) based on PDB ID: 3CMQ.

Molecular simulation studies and protein stability analysis

For molecular simulation studies, iGEMDOCK (http://gemdock.life.nctu.edu.tw/dock/igemdock.php) was used for FARS2 to perform ATP-docking analysis. Information on the key residues interacting with FARS2 and ATP was referenced from Elo JM et al.[46] In addition, Haddock (version 2.4, Computational Structural Biology Group, Utrecht University) was used for FARS2 to perform mt-tRNA^{Phe} docking analysis. Key binding residues between FARS2 and mt-tRNA^{Phe} were referenced from Klipcan L.[47] For protein stability analysis, DUET was performed (http://biosig.unimelb.edu.au/duet/stability).[48] Variant analysis was performed using PDB:3TUP as the structural basis of the wild type.

Constructs

The pcDNA3.1-HA-C plasmid was used for the expression of human FARS2 variants and purchased from Hunan Fenghui Biotechnology and validated by Sanger sequencing (Tsingke Biotechnology).

Cell culture and transfection

HeLa cells and A549 cells (obtained originally from ATCC) were cultured at 37°C in DMEM (Sigma) supplemented with 10% fetal bovine serum (FBS, Gibco), streptomycin (100 μg/ml) and penicillin (100 IU/ml) (Gibco). There two cells were seeded in 6-well plates (western blot assay) or 20 mm confocal dishes (immunofluorescence) and cultured for 12h before transfection using X-tremeGENE HP (Roche) and equal amounts of plasmid DNA.

Neonatal rat ventricular myocytes (NRVMs) were isolated from the ventricle of neonatal Sprague Dawley rats (1-3 days postnatal). Sterilize the neonatal rat by 75% ethyl alcohol, dissect the chest and excise their hearts. After removing the atria, fat, and aorta, the remaining hearts were washed with cold DPBS (Sigma), minced into ~1 mm^3 pieces, and digested using 1 mg/ml collagenase I (BioFROXX) at 37°C in water bath for 5-6 min. After digestion, the supernatant was collected and mixed with CMM (ScienCell). The digestion steps should be repeated 5-6 times. Cells were collected by centrifuging at 800 rpm for 5 min and further resuspended in CMM. After that, all the cells were seeded into T75 culture flask and cultured at 37°C with 95% air and 5% CO_2 for 90 min. Then, the supernatant containing NRVMs was transferred to a new plate for further assays. For *Fars2* knock down, NRVMs were infected with adenovirus carrying negative control (shCtrl) or knockdown of rat *Fars2* gene (sh-*Fars2*) at an MOI of 100 from Hanbio Tech (shanghai, China). The corresponding sequences are listed as follows (shCtrl target sequence: 5′-TTCTCCGAACGTGTCACGTAA-3′; sh-*Fars2* target sequence: 5′- CAGGACACCTCTGTTCTCCGTCTAT-3′). Mdivi-1 (Sigma, Cat#M0199, 50 μM) and 3-MA (MCE, Cat#HY-19312, 5 mM) were used for inhibition of mitochondrial fission and autophagy.

For assessment of autophagy flow, NRVMs were uniformly seeded in confocal dishes at an appropriate density. After different treatment, they were transiently transfected with fluorescent mCherry-GFP-LC3 adenovirus (1.58×10^{10} PFU/ml) according to manufacturer's instructions (Hanbio Tech, Shanghai, China). BafA1 (Sigma, Cat. #19-148) was added as a pharmacological agent used for blocking autolysosomes formation. Images were obtained using a confocal microscope (A1 PLUS, Nikon, Japan).

Western blot analysis

Cells, tissue and isolated mitochondrial samples were washed by ice-cold PBS and lysed with RIPA lysis buffer (Beyotime, Cat. #P0013B) supplemented with phosphatase and protease inhibitors (Roche). Lysates were separated on 10%-12.5% SDS-PAGE gels (Beyotime, Cat. # P0012AC), and then transferred to 0.22-0.45 μm polyvinylidene difluoride membranes (Merck Millipore) by Trans-Blot System (Bio-Rad Laboratories). Membranes were then blocked with 5% skimmed milk diluted in TBST for 1h at 25 ℃ and then were incubated in diluted primary antibodies overnight at 4℃. Subsequently, the membranes were incubated with secondary antibodies conjugated with horseradish peroxidase (HRP) for 1h at 25℃. Western HRP substrate (Millipore) and imaging system were employed to blot detection. The antibodies used in this study are as followed: HA (CST, Cat# 2367, ,1:1000), FARS2 (Invitrogen, Cat# PA5-100518, 1:1000), β-actin (Sigma, Cat# A1978, 1:10000), GAPDH (Proteintech, Cat# 60004-1-lg, 1:20000), HIS (Abways, Cat#AB0002, 1:1000), OXPHOS (Abcam, Cat# ab110413, 1:1000), ND3 (Novus, Cat# NBP2-93832), ND6 (Novus, Cat# NBP2-94464), CYB (LifeSpan BioSciences, Cat# 197737, 1:500), CO2 (Abcam, Cat# ab198286, 1:1000), LC3 (CST, Cat# 12741, 1:1000), P62 (Abcam, Cat# ab56416, 1:1000),

PINK1 (NOVUS, Cat# BC100-494, 1:1000), Parkin (CST, Cat# 4211, 1:1000), Ubiquitin (CST, Cat# 3936, 1:1000), TOMM20 (Abcam, Cat# ab186735, 1:1000), p-DRP1 (S616) (ABclonal, Cat# AP0849, 1:1000), p-DRP1 (S637) (CST, Cat# 4867, 1:1000), DRP1 (CST, Cat# 8570, 1:1000), MFN1 (Abcam, Cat# ab221661, 1:1000), MFN2 (Abcam, Cat# ab124773, 1:1000), OPA1 (Abcam, Cat# ab42364, 1:1000), PGC-1α (Abclonal, Cat# A11971, 1:1000).

Immunofluorescence

For patient samples, freshly isolated ventricular septal myocardial tissues were fixed in 4% formaldehyde buffer (PFA) at 4 °C for 24 h and embedded in paraffin. For mice samples, after euthanasia, mice were transcardially perfused with PBS followed by 4 % PFA. The hearts were isolated and embedded in paraffin. Successive 8 μm sections were cut for further experiments. Then, paraffin was removed by using xylene and samples were dehydrated using ethanol of graded concentrations. Antigen retrieval was completed using high pressure cooking in 1 mM EDTA buffer (pH=9.0). Tissue sections were blocked with 5% BSA and 15% goat serum in 1× PBS for 1 h at 25 ℃. Then, the primary antibodies were incubated at 4°C overnight and Alexa Fluor–conjugated secondary antibodies were incubated at 25 ℃ for 2 h. The following primary antibodies were used: VDAC1 (Abways, Cat#CY5416, 1:50); TOMM20 (Abcam, Cat# ab186735, 1:250); LC3 (CST, Cat# 12741, 1:100); SQSTM1/p62 (Abcam, Cat# ab56416, 1:100); α-actin (CST, Cat# 19245, 1:200); Parkin (CST, Cat# 4211, 1: 50). Alexa Fluor® AffiniPure IgG (H+L) were used as secondary antibodies. Nuclei were stained using DAPI (Sigma, Cat. # D9542).

For cells, cell lines and NRVMs were seeded on confocal dishes at an appropriate density.

After treatments, the cells were washed with DPBS twice and then fixed with 4 % formaldehyde buffer (PFA) at 37 °C for 20 min. After washing 2-3 times with PBS, the cells were permeabilized and blocked with 5% BSA and 15% goat serum in 1× PBS for 1 h at 25 °C. Then, the primary antibodies were incubated at 4°C overnight and Alexa Fluor–conjugated secondary antibodies were incubated at 25 °C for 2 h. DAPI was used to stain nuclei. All the graphs were viewed and taken by Nikon A1 PLUS. All the used antibodies were summarized as following: HA (CST, Cat# 2367, 1:100); TOMM20 (Abcam, Cat# ab186735, 1:250); LC3 (CST, Cat# 12741, 1:100); DRP1 (CST, Cat# 8570, 1:50).

Immunohistochemistry

After deparaffinization, serial sections (4 μm) were soaked in citric acid (pH=6.0) antigen retrieval buffer (Servicebio, Cat# G1203) and heated twice for 10 minutes for antigen retrieval in a microwave oven. Then, the sections were placed in 3% hydrogen peroxide solution and incubated at 25 °C in darkness for 25 minutes to block endogenous peroxidase activity. The sections were then incubated with primary antibodies against FARS2 (Invitrogen, Cat# PA5-100518, 1:20). HRP-labeled antibodies (Servicebio, Cat# GB23303, 1:200) were used as secondary antibodies. Nuclei were counterstained using hematoxylin (Servicebio), and diaminobenzidine (Servicebio, Cat# G1211) was served as the chromogen.

Generation of heterozygous cardiac-specific *Fars2* p.R415L mutant mice

Mouse *Fars2* transcripts were identified using the ENSEMBL database (reference transcript ID: NM_024274.3; ENSEMBL number: ENSMUST00000021857.12). *Fars2* conditional p.R415L mutant (*Fars2* ^{cMut}) mice were generated using the CRISPR/Cas9 system, according to the standard protocol (Cyagen Biosciences, China) (Figure S3A). The donor DNA with

LoxP pairs targeting the exon 7 of the *Fars2* transcript were constructed and confirmed via sequencing. The Cas9 protein, donor DNA, and two guide RNAs (gRNA-1: 5′-CTCTCCTAGTCAGGATTGACAGG-3′, gRNA-2: 5′-CTTCCCGGACAGGCTCGGGCTGG-3′) were microinjected into one-cell oocytes and transferred into pseudo-pregnant female mice. After crossing the *Fars2*$^{ML/-}$ with *Myh6*-Cre^{+} mice, the *cMut/-* line (*Fars2*$^{ML/-}$, *Myh6*-Cre^{+}) and the control littermates (*Fars2*$^{ML/-}$, *Myh6*-Cre^{-}) were identified using PCR (Figure S3B). The genotypes of mice were identified using the specific primers (Table S7).

Generation of *Fars2* inducible cardiac-specific knockout mice

Mouse *Fars2* transcripts were identified using the ENSEMBL database (reference transcript ID: NM_024274.3; ENSEMBL number: ENSMUST00000021857.12). FARS2 inducible knockout (icKO) mice were generated using CRISPR/Cas9 with standard procedures (Shanghai Model Organisms Center, Inc) (Figure S5A). The donor DNA with LoxP pairs targeting exon 3 of the *Fars2* transcript were constructed and confirmed by sequencing. The Cas9 protein, donor DNA and 2 guide RNA (gRNA1: 5′-AGCAAGCTCTGAGCTACCCAGGG-3′, gRNA2: 5′-GGACAAGATGCTTCACAATATGG-3′) were microinjected into one-cell oocytes and then transferred into pseudo-pregnant female mice. After crossing *Fars2*$^{LoxP/LoxP}$ with *Myh6*-MerCreMer^{+} mice, the experimental line (*Fars2*$^{LoxP/LoxP}$, *Myh6*-MerCreMer^{+}) and littermates (*Fars2*$^{LoxP/LoxP}$, *Myh6*-MerCreMer^{-}) were identified by DNA sequencing. The genotypes of mice were identified by specific primers (Table S7). All the 6-8 weeks old experimental mice were intraperitoneally injected with 4-OH Tamoxifen (Sigma, Cat. #579002, dissolved using

corn oil) for seven consecutive days in 20 mg/kg/day to induce *Fars2* knockout.

Generation of *fars2* knock down zebrafish

Adult wild-type zebrafish (AB strain) were maintained on a 14 h light/10 h dark cycle at 28.5℃. Five to six pairs of zebrafish were set up for natural mating every time. On average, 200–300 embryos were generated. The embryos were maintained at 28.5℃ in fish water (0.2% Instant Ocean Salt in deionized water). Morpholinos (MOs) were designed by Gene Tools, LLC (http://www.gene-tools.com/). MOs were microinjected into fertilized one-cell-stage embryos following standard protocols (Shanghai Model Organisms Center). The sequences of the *fars2* translation-blocking and splice-blocking morpholinos were summarized as follows (*fars2*-ATG-MO: 5′-CATAGTAGCTGGTCCATAAGCCTCT-3′; *fars2*-E3I3-MO: 5′-GAACATGGCAGGATTCCTACCTTCC-3′; *fars2*-Control-MO: 5′-CCTCTTACCTCAGTTACAATTTATA-3′).

PCR analysis for *fars2* exon 2 were used to confirm the efficacy of E3I3-MO and the primers sequences were listed in supplementary Table S7. The *ef1α* was used as the internal control.

Echocardiography in mice

Vevo 2100 High-Resolution Digital-Imaging System (Visual Sonics), equipped with an MS400 transducer, was used to analyze the echocardiography in mice. Briefly, mice were anaesthetized using isoflurane (1.5 % mixed with oxygen) in a closed box. Then mice were gently transferred to a 37 °C platform. Left ventricular function was assessed by ventricular M mode ultrasound at the papillary muscle level. All the mice were sacrificed with pentobarbital sodium (100mg/kg) by intraperitoneally injecting for further researches.

Morphological analysis

H&E, Masson's trichrome and wheat germ agglutinin (WGA) staining were employed for further morphological analysis. For patient samples, freshly isolated ventricular septal myocardial tissues were fixed in 4% formaldehyde buffer (PFA) at 4 °C for 24 h and embedded in paraffin. After euthanasia, mice were transcardially perfused with PBS followed by 4 % PFA. The hearts were isolated and embedded in paraffin. Successive 8 μm sections were cut for further experiments. These sections were stained with H&E and Masson's trichrome to evaluate morphology and the ventricular fibrosis. In addition, cardiomyocyte cross-sectional size was evaluated by WGA staining (Sigma). All the graphs were taken by Olympus VS200 and viewed by CaseViewer V.2.4.

Transmission electron microscopy

After washing with PBS, tissues (patients and mice) and cell samples were fixed in 2.5 % glutaraldehyde and 0.1 M sodium cacodylate buffer at 4℃ overnight. All the samples were embedded and subjected to ultramicrotomy. Transmission electron microscope images were captured by JEM-1400 (Japan Electron Optics Laboratory) and iTEM 5.2 (EMSIS). Quantification of the mitochondrial area (μm^2) and the mitochondrial number ($/\mu m^2$) were analyzed by Fiji Image J (NIH, Bethesda, MD, United States).

Myocardial gene delivery

Recombinant adeno-associated virus serotype-9 (AAV9) harboring full length mouse *Drp1* gene -targeted shRNA (*Drp1i*) or *Mfn1* gene (*Mfn1i*) with the cardiac troponin T (cTNT) promoter (AAV9-cTNT-*Drp1i* or AAV9-cTNT-*Mfn1*) and control vectors (AAV9-cTNT) were prepared and delivered as previously described.[49] All these AAV9-related vectors were

constructed by Hanbio Co, Ltd (Shanghai, China). pHBAAV-cTNT-Target vectors were constructed by cloning the target sequences into the pHBAAV-cTNT. After confirming the sequence, pHBAAV-cTNT-Target and control vectors were cloned into the recombinant AAV9 frame vector. All the AAV9 vectors were amplified in HEK293 cells.

Intra-myocardial injection was used to deliver the gene as previously described with minor modifications.[49] Briefly, mice were anesthetized by 2% isoflurane and a 1.5 cm skin cut was made on the left chest. The pectoral muscle was dissected and the ribs were exposed. The heart was smoothly and gently "popped out" through a small hole at the 4th intercostal space. AAV9 vectors were diluted to 2.5×10^{11} particles/ml in DPBS and 25 μl was directly injected into the left ventricle free wall using a 30.5 G Hamilton syringe (Hamilton Co, Reno, NV, USA). After the injection, the heart was immediately placed back into the chest, followed by manual evacuation of pneumothoraxes, closure of muscle, and the skin suture. One weeks after the injection, mice were intraperitoneally injected with 4-OH Tamoxifen to induce *Fars2* knockout.

Seahorse analyzer

Seahorse XF24 extracellular flux analyzer (Agilent Technologies) was employed to evaluate the mitochondrial respiratory capacity by measuring the oxygen consumption rate (OCR) and glycolytic capacity by measuring the extracellular acidification rate (ECAR). Briefly, NRVMs were seeded in an assay plate at 10^4 cells/well with CMM. For OCR, after specific stimulation, the medium was replaced with Seahorse basal medium (containing 1 mM pyruvate, 2 mM glutamine and 10 mM glucose) and was cultured at 37°C in a CO_2-free hub for 1h. The plate was injected with the following compounds sequentially: oligomycin (oligo, 1.5 mM), FCCP

(3mM), Rotenone/Antimycin A (Rot/AA, 0.5 mM) (XF Cell Mito Stress Text Kit, Agilent Technologies). For ECAR, cells were initially plated in XF Seahorse media with 2 mM glutamine in ECAR tests using the following concentrations of injected compounds, as indicated in the text: oligomycin, 2 μM; 2-DG, 100 mM; and glucose, 30 mM. The OCR or ECAR in each plate well was normalized with the protein content in each well (BCA Protein Assay Kit, BioVision).

ATP measurement

ATP levels in NRVMs and tissue were measured by enhanced ATP assay kits (Beyotime cat. #S0027, China) following by the recommendations of the manufacturer. The ATP concentration and standard curve were established by enzyme reader (TECAN cat. #30086376, Switzerland). The ATP was normalized with the protein content of each sample (BCA Protein Assay Kit, BioVision).

Reactive oxygen species (ROS) detection for tissue

After euthanasia, mice were transcardially perfused with PBS. Then, the hearts were isolated and embedded in optimal cutting temperature compound (OCT, Leica). Successive 8 μm sections were cut for further experiments by using freezing microtome (Leica, Cat#CM1950). These sections were stained with 1μM Dihydroethidium (Beyotime, Cat# S0063) to evaluate ROS contents. All the graphs were taken by Olympus VS200 and viewed by CaseViewer V.2.4.

Measurement of NAD level

The levels of NAD^+/NADH and total NAD were determined using NAD^+/NADH assay kit (Beyotime, Cat# S0175) following the manufacturer’s instructions. Briefly, NRVMs

(transfected with shCtrl or sh-*Fars2*) were lysed with lysis buffer (400 µl), and centrifuged at 12,000 ×g for 10 min. 90 µl of alcohol dehydrogenase was added to a 96-well plate. NAD total levels were obtained by adding 20 µl of the supernatant or standard substance. And NADH levels were obtained by adding 20 µl of the suspension or standard substance after incubating at 60 °C for 30 min and was added to a 96-well plate. Subsequently, 10 µl of chromogenic solution was added to the plate and the mixture was incubated at 37 °C for 30 min. The absorbance values were measured at 450 nm and analyzed on a multimode microplate reader (Tecan Spark). Standard curve was generated and the protein concentration of each sample was measured by BCA Protein Assay Kit (BioVision). The amount of NAD^+ was derived by subtracting NADH from total NAD.

Confocal microscopy

After specific treatment, NRVMs in confocal dished were washed by pre-warmed DPBS twice and followed by incubation with different dyes according to the manufacturer's protocol. Then, supernatants were discarded, and cells were washed twice using pre-warmed DPBS. Finally, the images of stained cells were taken on Nikon A1 PLUS. The dyes information was listed as following: TMRE (mitochondrial membrane potential detection, 0.2 µM, ThermoFisher, Cat. # T669); MitoSOX (mitochondria-derived ROS detection, 5 µM, ThermoFisher MitoSOX™ Red); Mito-Tracker Green (mitochondria detection, 0.2 µM, ThermoFisher, Cat. #M7514).

Bulk RNA-seq analysis

Total RNA was extracted using Axypre™ Multisource Total RNA Miniprep kit (Axygen) according to the manufacturer's protocol. RNA quality was evaluated by Agilent 2100

Bioanalyzer (Agilent Technologies, Palo Alto, CA, USA) and verified using RNase-free agarose gel electrophoresis. After treatment with Oligo(dT) beads, the enriched mRNA was then fragmented and reverse transcribed into cDNA using NEBNext Ultra RNA Library Prep Kit for Illumina (NEB #7530, New England Biolabs, Ipswich, MA, USA). The double-stranded cDNA products were then purified by QiaQuick PCR extraction kit (Qiagen, Venlo, The Netherlands), end repaired, added poly(A), and ligated with Illumina sequencing adapters. The adapter-ligated products were size selected by agarose gel electrophoresis. Finally, PCR amplification and sequencing were completed using Illumina Novaseq6000 by Gene Denovo Biotechnology Co. (Guangzhou, China). Raw reads were further filtered by fastp (V.18.0) to get high quality clean reads. Short reads alignment tool Bowtie2 (V.2.2.8) was used for mapping reads to ribosome RNA (rRNA) database. The rRNA mapped reads then will be removed. The remaining clean reads were further used in assembly and gene abundance calculation. An index of the reference genome was built, and paired-end clean reads were mapped to the reference genome (Ensembl_release100) using HISAT V.2.2.4 and other parameters set as a default. The mapped reads of each sample were assembled by using StringTie V.1.3.1 in areference-based approach. For each transcription region, a FPKM (fragment per kilobase of transcript per million mapped reads) value was calculated to quantify its expression abundance and variations, using RSEM software. RNAs differential expression analysis was performed by DESeq2 software between two different groups. The genes with a false-discovery rate (FDR) < 0.05 and fold-change $\geqslant 1.5$ or $\leqslant 1/1.5$ were considered differentially expressed genes. Pathway enrichment analysis and GO enrichment analysis was performed using the KEGG (https://www.kegg.jp/) and Gene Ontology database

(http://www.geneontology.org/), Bioinformatic analysis was performed by Gene Denovo Biotechnology Co., Ltd (Guangzhou, China). The RNA-seq data generated in this study have been deposited in the SRA database (ID: PRJNA973718; https://dataview.ncbi.nlm.nih.gov/object/PRJNA973718?reviewer=ob2n9oh1dl462ghi5j5ablv2dv).

Mitochondrial tRNA aminoacylation assay

Total mitochondrial RNAs were obtained using the Multisource Total RNA Miniprep Kit (Axygen) according to the manufacturer's instructions. Northern blot was carried out to detect the aminoacylation state of $tRNA^{Phe}$. In brief, 1 μg of total mitochondrial RNA were electrophoresed through a urea-denaturing 10% PAGE with 8 M urea gel in Tris borate-EDTA buffer to distinguish the charged and uncharged tRNA. The gels were electroblotted onto a positively charged nylon membrane (Merk emillipore) for hybridization analysis with DIG-labeled oligodeoxynucleotide probes for $tRNA^{Phe}$. DIG-labeled oligodeoxynucleotides (sequence: DIG-5′-GTTAATGTAGCTTAATAACAAAGCAAAGCACTGAAAATGCTTAGATGGATAATTGTATCCCATAAACA-3′) were generated using a DIG oligonucleotide tailing kit (Axl-Bio, axl-NBTZ001). The bands were detected using an automatic chemiluminescence imaging analysis system (Tanon5200).

RNA extraction and quantitative real-time polymerized chain reaction PCR (qRT-PCR)

Total RNA was extracted from cells and tissues following the standard protocols using Axypre™ Multisource Total RNA Miniprep kit (Axygen). The isolated RNA was then reverse-transcribed with PrimeScript™ RT Mix with gDNA eraser (Takara). SYBR®Premix

Ex Taq™ II (Takara) was employed for real-time fluorescent quantitative PCR by using 7500 system (Applied Biosystems). All the primers are listed in Table S7. The relative mRNA expression was analysis using the comparative threshold cycle method ($2^{-\Delta\Delta Ct}$) using GAPDH as the endogenous control gene.

Isolation of mitochondria

Mitochondria in heart tissue were isolated using Tissue Mitochondria Isolation Kit (Beyotime, Cat. #C3606, China) according to the manufacturer's protocol. Briefly, after heart isolation and washing by ice-cold PBS, the tissue was homogenized using a High-throughput tissue grinder (Servicebio) in isolation buffer A. Tissue lysis and mitochondrial isolation were completed by differential centrifugation. Purity of mitochondria was identified by flow cytometry analysis after staining with the mito-tracker Green (ThermoFisher, Cat. #M7514). For further western blot analysis, the mitochondrial pellets were washed twice with isolation buffer and then lysed by RIPA (Beyotime, Cat. #P0013B). Mitochondrial protein concentration was measured using a BCA Protein Assay Kit (BioVision) in duplicate.

Measurement of OXPHOs complexes activity

The activity of OXPHOs complexes were detected by enzymes activity assay kits according to the manufacturer's protocol. Briefly, mitochondria were extracted from heart tissues by lysis buffer. Then, the activity of five individual OXPHOs complexes were measured via detecting five specific enzymes. Specifically, the activity of mitochondrial complex Iwas reflected by the activity of NADH- coenzyme Q reductase (Solarbio, Cat# BC0515) ; the activity of mitochondrial complex II was reflected by the activity of succinate-coenzyme Q reductase (Solarbio, Cat# BC3235) ; the activity of mitochondrial complex III was reflected

by the activity of coenzyme Q-cytochrome C reductase (Solarbio, Cat# BC3245) ; the activity of mitochondrial complex IV was reflected by the activity of cytochrome C oxidase (Solarbio, Cat# BC0945) ; the activity of mitochondrial complex V was reflected by the activity of ATP synthase (Solarbio, Cat# BC1445) .

Mitochondrial DNA copy number (mtDNA-CN) quantification

Total DNA was isolated from tissues using QIAamp®DNA mini Kit (QIAGEN,Cat #51304, Germany). mtDNA-CN was assessed with qRT-PCR using SYBR®Premix Ex Taq™ II (Takara). The ratio of the mtDNA-encoded NADH dehydrogenase-1 (ND1) to the nuclear-encoded glyceraldehyde-3-phosphate dehydrogenase (GAPDH) were evaluated using the $2^{-\Delta\Delta Ct}$. All the primers are listed in Table S7. The relative fold change was further normalized to control group.

Flow cytometry analysis

After specific treatment, 1 million NRVMs were collected and stained with different dyes according to the manufacturer's protocol, followed by three washes with specific buffer and centrifugation at 800 rpm for 5 min at 25 ℃. Then, supernatants were discarded, and cells were resuspended in 300-500 μl buffer. Finally, the labeled cells were analyzed using a Coulter-XL and EXPO32 ADC Analysis software. The dyes information was listed as following: JC-1 (mitochondrial membrane potential detection, Sigma-Aldrich, Cat. #CS0390); DCFH-DA (ROS detection, Beyotime. Cat. #S0033S); Mito-Tracker Green (mitochondrial mass evaluation, 0.2 μM, ThermoFisher, Cat. #M7514).

Statistical Analysis

Data were expressed as mean ± s.e.m. Statistical analysis and graphical representation were

performed in the GraphPad Prism software (version 9.0.0) or Rstudio (version 4.1). Normality of the data was evaluated using the Shapiro-Wilk test. Two-tailed unpaired Student *t* tests were used to assess the differences between two groups, when normal distribution was satisfied; otherwise, the Mann–Whitney test was used. The differences between multiple groups were compared using one-way ANOVA or two-way ANOVA, followed by the Dunnett's (one-way) or Tukey’s (two-way) multiple comparisons test, when normal distribution was satisfied; otherwise, the nonparametric Kruskal–Wallis test was performed for further analysis. Two-way mixed-effects ANOVA followed by Sidak's multiple comparisons test was used to analyze differences in body weight at multiple time-points in order to account for correlation of repeated measurements of the same mouse (Figure S7C). The log-rank test was used to compare Kaplan-Meier survival estimates. Statistical significance was set at $P < 0.05$ (*); two asterisks (**) for $P < 0.01$; three asterisks (***) for $P < 0.001$; and four asterisks (****) for $P < 0.0001$.

Supplemental Tables

Table S1: Summaries for SNP-array results in Figure S1C

Chromosome	Chromosomal Position	dbSNP RS ID	Probe Set ID	Family 1-I-2		Family 1-II-1		Family 1-II-5		Family 1-II-7	
				Call Codes	Base Calls	Call Codes	Base Calls	Call Codes	Base Calls	Call Codes	Base Calls
6	5007636	rs811259	S-4NEIX	BB	CC	BB	CC	BB	CC	BB	CC
6	5036433	rs726743	S-4FAYI	AA	AA	AA	AA	AA	AA	AA	AA
6	5043572	rs59552394	S-4MNTS	BB	GG	BB	GG	BB	GG	BB	GG
6	5054515	rs11242987	S-3LKFC	BB	AA	BB	AA	BB	AA	BB	AA
6	5058939	rs1246995	S-4OMGR	BB	GG	BB	GG	BB	GG	BB	GG
6	5059671	rs1246997	S-3ZZNC	AA	AA	AA	AA	AA	AA	AA	AA
6	5106145	rs17139431	S-4IZLD	BB	CC	BB	CC	BB	CC	BB	CC
6	5118017	rs375064	S-3YGFN	BB	GG	BB	GG	AB	AG	BB	GG
6	5122495	rs736004	S-4ITRB	BB	CC	BB	CC	BB	CC	BB	CC
6	5137685	rs457453	S-3URLH	AA	TT	AA	TT	AB	TC	AA	TT
6	5144671	rs200866	S-3AMNX	BB	TT	BB	TT	BB	TT	BB	TT
6	5146806	rs10484845	S-4ANJD	AB	TC	AA	TT	AB	TC	AB	TC
6	5147416	rs73717686	S-4FJSS	AB	AT	BB	TT	AB	AT	AB	AT
6	5150783	rs60478717	S-3FDTX	AB	AG	AA	AA	AB	AG	AB	AG
6	5158991	rs2746242	S-3MMWP	AB	AC	AA	AA	AB	AC	AB	AC
6	5172547	rs439356	S-3QRJG	AB	GA	AB	GA	AB	GA	BB	AA
6	5176905	rs7763280	S-3PVAA	AB	CT	AB	CT	AB	CT	BB	TT
6	5233112	rs61089858	S-4AYXU	BB	GG	BB	GG	BB	GG	BB	GG
6	5236647	rs9392665	S-4PPPV	AB	GC	AB	GC	AA	GG	AA	GG
6	5240666	rs2773296	S-4KOVP	AB	CT	AB	CT	BB	TT	BB	TT
6	5245885	rs13212749	S-4IXTN	BB	TT	BB	TT	BB	TT	BB	TT
6	5248305	rs2753230	S-4DPTR	AB	CG	AB	CG	AA	CC	AA	CC
6	5272438	rs11752747	S-4MRQU	AA	AA	AA	AA	AB	AG	AA	AA
6	5276208	rs78640466	S-4MDQD	BB	CC	BB	CC	BB	CC	BB	CC

6	5295202	rs17140122	S-3HUPI	AA	AA	AA	AA	AA	AA	AA	AA
6	5305382	rs7765867	S-4JUZH	AA	CC	AA	CC	AB	CA	AA	CC
6	5323458	rs9392076	S-3GRKD	AB	AC	AB	AC	AB	AC	BB	CC
6	5331350	rs11243003	S-4JBCZ	AB	CT	AB	CT	AA	CC	AA	CC
6	5335239	rs797134	S-3GORH	BB	AA	BB	AA	BB	AA	BB	AA
6	5370723	rs2432807	S-3MGNJ	AB	AG	AB	AG	AA	AA	AA	AA
6	5378995	rs2432749	S-3RQHE	AB	CT	AB	CT	AA	CC	AA	CC
6	5384551	rs62385366	S-4KNBL	AB	CT	AB	CT	AA	CC	AA	CC
6	5390457	rs2503813	S-4HZDT	AB	TC	AB	TC	AA	TT	AA	TT
6	5391035	rs2503815	S-3BEMS	AA	GG	AA	GG	AA	GG	AA	GG
6	5395052	rs2032989	S-3GFED	BB	TT	BB	TT	BB	TT	BB	TT
6	5396363	rs11243010	S-3NPTY	AB	GT	AA	GG	AA	GG	AA	GG
6	5407231	rs2432771	S-4PBIC	AB	CT	AB	CT	AA	CC	AA	CC
6	5434845	rs2503835	S-3MLVE	BB	AA	BB	AA	BB	AA	BB	AA
6	5475252	rs433295	S-4NBCJ	BB	GG	BB	GG	BB	GG	BB	GG
6	5481160	rs17140553	S-3MIZB	BB	TT	BB	TT	BB	TT	BB	TT
6	5482270	rs2432804	S-3KKMK	AA	TT	AB	TC	AA	TT	AB	TC
6	5483381	rs9504408	S-4ETDK	AA	CC	AA	CC	AA	CC	AA	CC
6	5487805	rs408705	S-4LZNG	AA	TT	AA	TT	AA	TT	AA	TT
6	5491835	rs12212484	S-4RAUZ	BB	TT	BB	TT	BB	TT	BB	TT
6	5519892	rs9502311	S-3OEKB	AB	GA	BB	AA	AB	GA	BB	AA
6	5519921	rs73356365	S-3ZUJI	BB	GG	BB	GG	BB	GG	BB	GG
6	5528649	rs10458088	S-4APDN	BB	CC	BB	CC	BB	CC	BB	CC
6	5540871	rs9504431	S-3KPXH	BB	AA	AB	GA	BB	AA	AB	GA
6	5544813	rs2326613	S-4DUDN	BB	GG	BB	GG	BB	GG	BB	GG
6	5545421	rs7749599	S-4KRGF	BB	TT	BB	TT	BB	TT	BB	TT
6	5556464	rs9504434	S-3DUUD	BB	TT	AB	CT	AB	CT	AB	CT
6	5562313	rs9378429	S-3XBEW	BB	TT	BB	TT	BB	TT	BB	TT
6	5575408	rs4960103	S-4SGGH	BB	GG	BB	GG	BB	GG	BB	GG

6	5593732	rs1985346	S-3SLTU	AA	GG	AA	GG	AA	GG	AA	GG
6	5593847	rs9405853	S-4PJTL	BB	GG	BB	GG	BB	GG	BB	GG
6	5594055	rs9502319	S-4RYAY	AA	CC	AA	CC	AA	CC	AA	CC
6	5596751	rs9378969	S-3CXOB	AA	TT	AA	TT	AA	TT	AA	TT
6	5599615	rs9504442	S-4FCTV	BB	AA	BB	AA	BB	AA	BB	AA
6	5600205	rs9328321	S-4NJWH	BB	GG	BB	GG	BB	GG	BB	GG
6	5603880	rs9504449	S-4GNPW	AA	GG	AA	GG	AA	GG	AA	GG
6	5606543	rs7747835	S-3VWKQ	AA	AA	AA	AA	AA	AA	AA	AA
6	5612347	rs7755407	S-4KEQR	AA	TT	AA	TT	AB	TC	AA	TT
6	5612642	rs9328323	S-3IOVU	AB	TC	AB	TC	AB	TC	BB	CC
**6**	**5623153**	**rs7762094**	**S-3EQOU**	**AA**	**CC**	**AA**	**CC**	**AA**	**CC**	**AA**	**CC**
**6**	**5647854**	**rs10484314**	**S-3LXAH**	**AB**	**GA**	**AA**	**GG**	**AB**	**GA**	**AB**	**GA**
**6**	**5659632**	**rs10484315**	**S-3ULOP**	**BB**	**AA**	**BB**	**AA**	**BB**	**AA**	**BB**	**AA**
**6**	**5675612**	**rs2142738**	**S-3QXXJ**	**BB**	**TT**	**BB**	**TT**	**BB**	**TT**	**BB**	**TT**
**6**	**5676226**	**rs9405859**	**S-3DCLA**	**BB**	**GG**	**BB**	**GG**	**BB**	**GG**	**BB**	**GG**
**6**	**5676331**	**rs9378979**	**S-4CWYY**	**BB**	**AA**	**BB**	**AA**	**BB**	**AA**	**BB**	**AA**
**6**	**5682196**	**rs9504488**	**S-3HGKT**	**BB**	**GG**	**BB**	**GG**	**BB**	**GG**	**BB**	**GG**
**6**	**5682920**	**rs4960125**	**S-4LNUL**	**AA**	**GG**	**AA**	**GG**	**AA**	**GG**	**AA**	**GG**
**6**	**5684092**	**rs76121789**	**S-3ZUKZ**	**AA**	**CC**	**AA**	**CC**	**AA**	**CC**	**AA**	**CC**
**6**	**5709995**	**rs13210778**	**S-3THAM**	**AA**	**TT**	**AA**	**TT**	**AA**	**TT**	**AA**	**TT**
**6**	**5744759**	**rs7740019**	**S-3DNIP**	**AB**	**GA**	**AA**	**GG**	**AB**	**GA**	**AB**	**GA**
**6**	**5748068**	**rs9504506**	**S-4PJCN**	**AA**	**AA**	**AA**	**AA**	**AA**	**AA**	**AA**	**AA**
**6**	**5757417**	**rs2326673**	**S-3OJGN**	**AB**	**CA**	**AA**	**CC**	**AB**	**CA**	**AB**	**CA**
**6**	**5803522**	**rs957530**	**S-3PFLT**	**AA**	**GG**	**AA**	**GG**	**AA**	**GG**	**AA**	**GG**
**6**	**5821999**	**rs4604310**	**S-3CSDH**	**AA**	**TT**	**AA**	**TT**	**AA**	**TT**	**AA**	**TT**
**6**	**5829482**	**rs13219662**	**S-4KVPL**	**BB**	**CC**	**BB**	**CC**	**BB**	**CC**	**BB**	**CC**
**6**	**5860948**	**rs2146455**	**S-3ERMX**	**BB**	**AA**	**BB**	**AA**	**BB**	**AA**	**BB**	**AA**
**6**	**5865410**	**rs41488745**	**S-4FMUR**	**BB**	**CC**	**BB**	**CC**	**BB**	**CC**	**BB**	**CC**
**6**	**5877417**	**rs12526809**	**S-4TCYP**	**AA**	**TT**	**AA**	**TT**	**AA**	**TT**	**AA**	**TT**

**6**	**5879688**	**rs79834396**	**S-4GBIZ**	**AA**	**AA**	**AA**	**AA**	**AA**	**AA**	**AA**	**AA**
**6**	**5884626**	**rs1407793**	**S-4QTOY**	**BB**	**AA**	**BB**	**AA**	**BB**	**AA**	**BB**	**AA**
**6**	**5885085**	**rs56971956**	**S-3NVOS**	**BB**	**GG**	**BB**	**GG**	**BB**	**GG**	**BB**	**GG**
**6**	**5903761**	**rs7744130**	**S-4QSCL**	**BB**	**GG**	**BB**	**GG**	**BB**	**GG**	**BB**	**GG**
**6**	**5905226**	**rs6597170**	**S-3WVNE**	**BB**	**GG**	**BB**	**GG**	**BB**	**GG**	**BB**	**GG**
**6**	**5908931**	**rs7775306**	**S-4FZHE**	**BB**	**GG**	**BB**	**GG**	**BB**	**GG**	**BB**	**GG**
**6**	**5923459**	**rs6919620**	**S-3VLMP**	**AA**	**GG**	**AA**	**GG**	**AA**	**GG**	**AA**	**GG**
**6**	**5939520**	**rs9504590**	**S-4NKFM**	**BB**	**GG**	**BB**	**GG**	**BB**	**GG**	**BB**	**GG**
6	5946906	rs4959364	S-3RKKG	BB	GG	AB	AG	AB	AG	AB	AG
6	5947650	rs9405884	S-3AAZQ	AB	CT	AA	CC	AB	CT	AB	CT
6	5955032	rs9405886	S-4FXGS	AB	AT	BB	TT	AB	AT	AB	AT
6	5959820	rs9504609	S-3KQZC	AA	CC	AB	CT	AB	CT	AB	CT
6	5961368	rs1555536	S-3IEMF	AB	AG	AA	AA	BB	GG	AB	AG
6	5968121	rs78705825	S-4GDMI	AA	CC	AA	CC	AA	CC	AA	CC
6	5970683	rs6930257	S-3EHVL	BB	TT	BB	TT	AB	CT	BB	TT
6	5976806	rs6907426	S-4EUHH	AA	GG	AB	GA	AB	GA	AB	GA
6	5981067	rs882529	S-4GQRX	BB	GG	BB	GG	BB	GG	BB	GG
6	5983543	rs6597173	S-3HWPX	BB	CC	BB	CC	BB	CC	BB	CC
6	5990591	rs17363382	S-4KBVJ	NoCall		BB	AA	BB	AA	BB	AA
6	5995628	rs7748529	S-4SMVS	AB	CA	BB	AA	AB	CA	AB	CA

Table S2: *In silico* analysis of the *FARS2* variants

Family			Patient 1 (Family 1)	Patient 2	Patient 3, 4	Patient 5	Patient 6	Patient 7	Patient 8
Variant position			c.1244G>T (6/5771317)	c.21G>T (6/5368591)	c.308G>T (6/53688878)	c.1051A>C (6/55453326)	c.1220C>T (6/57712293)	c.682C>G (6/5404611)	c.1157G>A (6/5613260)
			p.R415L	p.R7S	p.G103V	p.K351Q	p.T407M	p.Q228E	p.R386Q
Predictions	Aggregated	Aggregated Prediction	Deleterious (0.79)	Benign (0.06)	Benign (0.14)	Benign (0.09)	Uncertain (0.4)	Uncertain (0.46)	Deleterious (0.87)
	Functional Coding	MUT Assesor	Hi (3.7)	Lo (0.9)	Lo (1.61)	Lo (1.25)	Med (2.86)	Med (2.9)	Med (3.29)
		SIFT	Damaging (0)	Tolerated (0.72)	Tolerated (0.22)	Tolerated (0.6)	Damaging (0.03)	Deleterious (Supporting) (0)	Uncertain (0.001)
		DANN	Deleterious (1)	Deleterious (low) (0.73)	Deleterious (0.96)	Benign (low) (0.49)	Deleterious (1)	N/A	N/A
		MetaLR	Deleterious (0.7)	Benign (0.05)	Benign (0.12)	Benign (0.07)	Benign (low) (0.39)	Benign (low) (0.34)	Deleterious (low) (0.68)
		REVEL	Deleterious (0.87)	Benign (0.07)	Benign (low) (0.16)	Benign (0.1)	Benign (low) (0.41)	Uncertain (0.36)	Deleterious (Moderate) (0.9)
		PrimateAI	Tolerated (0.6)	Tolerated (0.32)	Tolerated (0.47)	Tolerated (0.24)	Tolerated (0.57)	N/A	N/A
		BayesDel	Damaging (0.36)	Tolerated (-0.47)	Tolerated (low) (-0.29)	Tolerated (-0.57)	Tolerated (low) (-0.15)	Uncertain (-0.16)	Deleterious (Moderate) (0.35)
		CADD phred (raw score)	31 (4.35)	5.25 (0.16)	15.90 (1.43)	18.16 (1.79)	26.30 (3.80)	25.6 (3.653222)	32 (4.482680)
		VARITY	Deleterious (0.97)	Benign (0.09)	Deleterious (low) (0.33)	Benign (low) (0.14)	Deleterious (low) (0.38)	Deleterious (0.76)	Deleterious (0.84)
		Mutation Taster	Deleterious (1)	Benign (0)	Deleterious (1)	Benign (0)	Deleterious (1)	Deleterious (1)	Deleterious (1)
	Splice Altering	SpliceAI	Benign (0)	Benign (0)	Benign (0)	Benign (0.02)	Benign (0)	Benign (0)	Benign (0)
		dbscSNV Ada	N/A	N/A	N/A	N/A	N/A	N/A	N/A
		RF	N/A	N/A	N/A	N/A	N/A	N/A	N/A
	Conservation	GERP	High Constraint (5.81)	Low Constraint (0.3)	Low Constraint (2.43)	Medium Constraint (3.05)	High Constraint (5.81)	Uncertain (5.32)	Uncertain (5.53)
		Conservation score	9	3	3	1	7	9	9
	Functional Whole Genome	GenoCanyon	Deleterious (1)	Benign (0)	Benign (0.02)	Benign (0.03)	Deleterious (0.99)	Deleterious (1)	Deleterious (1)
		fitCons	Deleterious (0.66)	Deleterious (0.71)	Deleterious (0.71)	Deleterious (0.71)	Deleterious (0.66)	Deleterious (0.71)	Deleterious (0.71)
	Mitochondrial	MitoTip	N/A	N/A	N/A	N/A	N/A	N/A	N/A
		APOGEE	N/A	N/A	N/A	N/A	N/A	N/A	N/A

Population Frequencies	gnomAD (Aggregated)	N/A	0.0057%	0.0074%	0.0057%	0.005%	N/A	0.0008%
	gnomAD_EAS	N/A	0.0752%	0.1054%	0.0803%	0.0351%	N/A	0.0109%
	China MAP	N/A	0.0614%	0.0661%	0.0708%	0.0567%	N/A	N/A
	TOPMed Bravo	N/A	N/A	N/A	N/A	0.0034%	N/A	N/A
	GME Variome	N/A	N/A	N/A	N/A	N/A	N/A	N/A
	1000 Genomes	N/A	N/A	N/A	N/A	N/A	N/A	N/A
	ESP 6500	N/A	N/A	N/A	N/A	0.0077%	N/A	N/A
	4.7KJPN	N/A	N/A	N/A	N/A	0.01%	N/A	N/A
	GenomeAsia	N/A	N/A	0.0289%	N/A	0.0289%	N/A	N/A

Table S3: Case-Control Association Studies: observed allele counts for all variants screened in our cases and the China Metabolic Analytics Project reference database

cDNA	Protein	Index Cases Allele Counts		GnomAD_EAS Allele Counts[†]					China MAP Allele Counts[†]				
		mutant	all	mutant	all	OR[‡]	95% CI (OR)	*P* value[‡]	mutant	all	OR[‡]	95% CI (OR)	*P* value[‡]
**c.1244G>T**	**p.R415L**	**1**	**1141**	**0**	**19948**	**Infinity**	**1.941- Infinity**	**0.0541**	**0**	**21176**	**Infinity**	**2.060 - Infinity**	**0.0512**
c.21G>T	p.R7S	1	1141	15	19940	1.165	0.1100 - 6.739	0.5896	13	21176	1.428	0.1336 – 8.644	0.5206
c.308G>T	p.G103V	2	1141	21	19924	0.8315	0.3841 - 6.118	0.3566	14	21176	2.651	0.5970– 11.01	0.1961
c.1051A>C	p.K351Q	1	1141	16	19934	1.092	0.1034 - 6.206	0.6120	15	21176	1.237	0.1168 – 7.157	0.5683
c.1220C>T	p.T407M	1	1141	7	19948	2.498	0.2214 - 17.52	0.3594	12	21176	1.547	0.1439 – 9.654	0.4947
**c.682C>G**	**p.Q228E**	**1**	**1141**	**0**	**19948**	**Infinity**	**1.941 - Infinity**	**0.0541**	**0**	**21176**	**Infinity**	**2.060 - Infinity**	**0.0512**
**c.1157G>A**	**p.R386Q**	**1**	**1141**	2	18372	8.051	0.5550 - 69.29	0.1655	**0**	**21176**	**Infinity**	**2.060 - Infinity**	**0.0512**

[†] Observed allele counts for all variants screened in our cases and the exomes or exome and genome collections of allele counts in the GnomAD_EAS and China Metabolic Analytics Project reference.

[‡] An OR of 1 indicates no difference between the frequency of cases and controls. The nominal *P*-value is the probability that the observed OR is greater than expected by random chance (> 1) (using two-sided Fisher's exact test).

Table S4: Summaries of the ACMG/AMP evidentiary for all *FARS2* variants

Classification†	cDNA	Protein	Pathogenic Criteria																BA1	Benign Criteria										
				Strong				Moderate						Supporting						Strong				Supporting						
			PVS1	PS1	PS2	PS3	PS4	PM1	PM2	PM3	PM4	PM5	PM6	PP1	PP2	PP3	PP4	PP5	BA1	BS1	BS2	BS3	BS4	BP1	BP2	BP3	BP4	BP5	BP6	BP7
Uncertain	c.1244G>T	p.R415L							√					√		√	√													
Uncertain	c.21G>T	p.R7S															√													
Uncertain	c.308G>T	p.G103V														√	√													
Uncertain	c.1051A>C	p.K351Q															√										√			
Uncertain	c.1220C>T	p.T407M														√	√													
Uncertain	c.682C>G	p.Q228E							√							√	√													
Uncertain	c.1157G>A	p.R386Q							√							√	√													

† Classification of these risk allele meets the rules for combining criteria to classify sequence variants according to the ACMG/AMP guidelines (https://www.acmg.net/ACMG).

√ Variants meets the standard for corresponding criteria according to the ACMG/AMP guidelines (https://www.acmg.net/ACMG).

Table S5: Protein Stability Change Upon the *FARS2* variants

Conformation of FARS2	Protein Stability Change Upon variants	Details	p.R7S	p.G103V	p.Q228E	p.K351Q	p.R386Q	p.T407M	p.R415L
Closed	mCSM (ΔΔG, kcal/mol)	value	-	-0.486	-2.019	-0.055	-1.347	-0.1	-0.275
		classification	-	Destabilizing	Destabilizing	Destabilizing	Destabilizing	Destabilizing	Destabilizing
	SDM (ΔΔG, kcal/mol)	value	-	-0.12	0.28	-0.59	-2.05	0.11	0.47
		classification	-	Destabilizing	Stabilizing	Destabilizing	Destabilizing	Stabilizing	Stabilizing
	DUET (ΔΔG, kcal/mol)	value	-	-0.119	-1.851	0.076	-1.645	-0.07	0.027
		classification	-	Destabilizing	Destabilizing	Stabilizing	Destabilizing	Destabilizing	Stabilizing
Open	mCSM (ΔΔG, kcal/mol)	value	-	-0.504	-1.961	-0.187	-0.271	-0.052	-0.495
		classification	-	Destabilizing	Destabilizing	Destabilizing	Destabilizing	Destabilizing	Destabilizing
	SDM (ΔΔG, kcal/mol)	value	-	0.09	0.28	-0.62	-0.15	0.09	0.47
		classification	-	Stabilizing	Stabilizing	Destabilizing	Destabilizing	Stabilizing	Stabilizing
	DUET (ΔΔG, kcal/mol)	value	-	-0.17	-1.789	-0.066	-0.014	-0.025	-0.177
		classification	-	Destabilizing	Destabilizing	Destabilizing	Destabilizing	Destabilizing	Destabilizing

Table S6: Docking analysis of the *FARS2* variants

Wild type or variant position		WT	p.R7S	p.G103V	p.Q228E	p.K351Q	p.R386Q	p.T407M	p.R415L	p.D142Y
mt-tRNA^{Phe}	Docking score	-535.06	-	-330.87	-521.89	-526.73	-517.03	-409.54	-479.48	-481.93
	Ligand rmsd (Å)	0.47	-	2.31	0.49	0.32	0.43	2.09	0.54	0.53
ATP-docking score		-157.194053	-	-119.902	-102.263361	-97.758902	-105.703638	-123.263032	-91.984510	-106.333456

Table S7: Primers used for the different assays in this study

Assays	Species	Target genes	Forward primers (5'-3')	Reverse primers (5'-3')	Figure
PCR	Human	*FARS2*-Exon-1	CTGTGCGGAAACCACGAAC	TTGACAGAAAGCGGGCCT	Figure 1, S1-2, S8
		FARS2-Exon-2	GCTGTGTGGAGGTCGTAGTG	GCAAGCAAGGATGACTATGCTG	
		FARS2-Exon-3	TGACAGGACATAATGAGTCTTATTCCC	TCCAGCCTGGGTGACAGAG	
		FARS2-Exon-4	TGATCACAAGAAAGGGCAGACAGC	GACCAGTGATGCCGCTGC	
		FARS2-Exon-5	TTTGCCCGCTGGTAGGCA	AGTGGCTTTCAAGTACATGATCCTGT	
		FARS2-Exon-6	CATTTCCTAATTAGTCAAGTGCAACT	CCCTGGGTTTCAGCAAATTTT	
		FARS2-Exon-7	GGCAAGCCACACTGCTCC	CCATACTAAGCACCAATGCCAGTG	
Transfection efficiency (qRT-PCR)	Plasmid	*AmpR*	ACCCAGAAACGCTGGTGAAA	GGGGCGAAAACTCTCAAGGA	Figure S3
mRNA expression level (qRT-PCR)	Human	*FARS2-HA*	CACGTACCGCCACATGGAACG	TCAGAACCTGCCCTCCAC	
qRT-PCR	Human	*β-actin*	CATGTACGTTGCTATCCAGGC	CTCCTTAATGTCACGCACGAT	
RT-PCR	zebrafish	*fars2*	CACTATCCCGTCTTCCATCAG	TGAAAGAACACCTCCATCTCG	Figure S7
		ef1α	GGAAATTCGAGACCAGCAAATAC	GATACCAGCCTCAAACTCACC	
mtDNA-CN/ qRT-PCR	Rat	*Gapdh*	GCGAGATCCCGCTAACATCA	CTCGTGGTTCACACCCATCA	Figure S10, S12, 6
qRT-PCR		*Fars2*	GCCATTGCTACCGCATTACC	CAAAAACGGCCTTCCACACC	Figure S10
mtDNA-CN/ qRT-PCR		*Nd1*	ATGGCCTTCCTCACCCTAGT	GTTAGGGGGCGTATGGGTTC	Figure S10, S12, 6
PCR	Mouse	$Fars2^{Mut\text{-}LoxP}$	CCATCTGAAACCCACTTGC	CTCTTCCTGCTACCTGTCAATC	Figure S4
PCR		*Myh6-Cre*	GACAGACAGATCCCTCCTATCTCC	GTTCTTGCGAACCTCATCACTC	
RT-PCR		Transcript 1 ($Fars2^{Mut\text{-}LoxP\ (ML)}$)	TTTCAGCCTCTTAGCAAGTAC	CCCGGACTATGCAGGATCCTAT	
RT-PCR		Transcript 2 ($Fars2^{cMut\ (R415L)}$)	AAGTACCCCGCCGTGTTCAA	CACCATCACCATCATCACCAC	
PCR		$Fars2^{L/L}$	GAGGGAGCCTTTTGGTCTTTCATCTTGC	CGACAGTATCCCCTTCCCCACCCC	Figure S7
PCR		*Myh6-MerCreMer*	TCTATTGCACACAGCAATCCA	CCAGCATTGTGAGAACAAGG	
mtDNA-CN/ qRT-PCR		*Gapdh*	TGACCTCAACTACATGGTCTACA	CTTCCCATTCTCGGCCTTG	Figure 2, 4, 6, 7, S14
qRT-PCR		*Fars2*	GAGGAAGGTCAGTCATGCCTG	TCTGGGTGAGGTTAGTGTGGT	Figure 7
qRT-PCR		*Nppa*	GCTTCCAGGCCATATTGGAG	GGGGGCATGACCTCATCTT	Figure 2, 7
qRT-PCR		*Nppb*	GAGGTCACTCCTATCCTCTGG	GCCATTTCCTCCGACTTTTCTC	
qRT-PCR		*Myh7*	CCTGCGGAAGTCTGAGAAGG	CTCGGGACACGATCTTGGC	
qRT-PCR		*Atg5*	CACCCCTGAAATGGCATTATCC	TGGACAGTGTAGAAGGTCCTTT	Figure S14
qRT-PCR		*Atg7*	TCTGGGAAGCCATAAAGTCAGG	GCGAAGGTCAGGAGCAGAA	
qRT-PCR		*Map1lc3b*	TTATAGAGCGATACAAGGGGGAG	CGCCGTCTGATTATCTTGATGAG	
qRT-PCR		*Map1lc3a*	GACCGCTGTAAGGAGGTGC	CTTGACCAACTCGCTCATGTTA	
qRT-PCR		*Mfn1*	AACTTGATCGAATAGCATCCGAG	GCATTGCATTGATGACAGAGC	
qRT-PCR		*Mfn2*	CTGGGGACCGGATCTTCTTC	CTGCCTCTCGAAATTCTGAAACT	
qRT-PCR		*Opa1*	TGGAAAATGGTTCGAGAGTCAG	CATTCCGTCTCTAGGTTAAAGCG	
qRT-PCR		*Dnm1l (Drp1)*	TAAGCCCTGAGCCAATCCATC	CATTCCCGGTAAATCCACAAGT	
qRT-PCR		*Fis1*	AGGCTCTAAAGTATGTGCGAGG	GGCCTTATCAATCAGGCGTTC	
qRT-PCR		*Ppargc1a*	TATGGAGTGACATAGAGTGTGCT	GTCGCTACACCACTTCAATCC	
qRT-PCR		*Tfam*	AACACCCAGATGCAAAACTTTCA	GACTTGGAGTTAGCTGCTCTTT	
mtDNA-CN/ qRT-PCR		*Nd1*	AACCTCAACCTAGGCCTCCT	TGTTTGGGCTACTGCTCGC	Figure 4, 6
qRT-PCR		*Nd2*	TTTACCCGCTACTCAACTATAC	CATCCTATGTGGGCAATTGATG	
qRT-PCR		*Nd3*	CTACTTCCACTACCATGAGCAACCACT	TGTTCATTCATATGCTAGGCCT	
qRT-PCR		*Nd6*	CATTCATTGACCTACCT	GCTCCGTTTGCGTGTATATATC	
qRT-PCR		*Cytb*	CCACTCATTCATTGACCTACCT	GCTCCGTTTGCGTGTATATATC	
qRT-PCR		*Cox1*	ACTCCTACCACCATCATTTCTCC	GGCTAGATTTCCGGCTAGAGG	
qRT-PCR		*Cox2*	CTTGGTCTACAAGACGCCAC	CTATTGGCAGAACGACTCGG	
qRT-PCR		*Cox3*	ACACTATTAACCCTTGGCCTAC	AGGCTAGAATGATAGAACGCTC	
qRT-PCR		*Atp6*	AAGCTCACTTGCCCACTTCC	GTAAGCCGGACTGCTAATGC	
qRT-PCR		*Atp8*	CTCATCACAAACATTCCCACTG	TGGGGTAATGAATGAGGCAAAT	

Supplemental Figure legends:

Figure S1. Genetic analysis pipeline for family 1.

A. Variant identification pipeline for family 1.

B. The Sanger sequencing results of *ADA* : c.872C>T in family 1.

C. The SNP-array results of family 1-I-2, II-1, II-5, and II-7 were obtained for the genomic region ch6:5,000,000 to ch6:6,000,000. The potential homozygous region of individual II-1 was highlighted with a yellow rectangle. The *FARS2* gene and its corresponding exon regions are displayed above. The variant (c.1244G>T) was indicated by a red arrow.

Figure S2. Screening analysis pipeline, validation and conservation analyses for HCM-related *FARS2* variants.

A, Variant identification pipeline of *FARS2* related variants.

B, ECG results of family 1-II-3.

C-I, Pedigrees and Sanger validation of *FARS2* variants in patient 2 (**C**, c.21G>T), patient 3 (**D**, c. 308 G>T), patient 4(**E**, c. 308 G>T), patient 5(**F**, c. 1051 A>C), patient 6(**G**, c. 1220 C>T), patient 7 (**H**, c. 682C>G), and patient 8 (**I**, c. 1157G>A).

J, Summary of the residue variety in % for each identified variant in FARS2 across homologues of 150 species following ClustalW-based multiple sequence alignment using the ConSurf server (see Methods). Human wild-type FARS2 residues were indicated in blue and human FARS2 variants in red.

ECG indicates electrocardiogram.

Figure S3. The functional analysis of FARS2 variants in A549 cells and protein stability studies of the FARS2 variants.

A, Expression level of FARS2 from A549 cells transfected with empty vector control, WT or 7 variants vectors. The relative statistical analysis was shown in the lower panel (n=3 per group).

B, Confocal images of A549 cells transfected with HA-FARS2 constructs and immunolabeled with TOMM20 (mitochondrial marker). The regions were magnified with split channels beneath each group and boxed with red (HA) or green (TOMM20). The representative co-localization parts were emphasized by blue arrows, while the non co-localization parts were highlighted by red asterisks. Scare bar=10 μm.

C-E, The evaluation of transfection efficiency (**C**), mRNA expression levels & stability (**D**) and protein turnover (**E**) of HeLa cells transfected with WT or 7 variants vectors (see methods). A schematic of the experimental design was shown in the upper panel. Relative statistical analysis (relative to WT) and western blotting results were shown in the lower panel (n=3 per group).

**P<0.01; ***P<0.001; ****P<0.0001. WT indicates wild type.

Figure S4. Establishment, identification and phenotypic characteristics of heterozygous cardiac-specific *Fars2* p.R415L mutant model *in vivo*

A, Targeting strategy for the cardiac specific *Fars2* p.R415L mutant of *Fars2* gene ($Fars2^{cMut}$). Blue arrows indicated the genome identification primers. Red asterisk indicated the variant of c.1244G>T.

B, Genotype analysis of control (*Fars2* $^{ML/-}$, *Myh6*-Cre^{-}) and *Fars2* $^{cMut/-}$ (*Fars2* $^{ML/-}$, *Myh6*-Cre^{+}) mice. DNA fragments corresponding to the LoxP (114 and 170 bp) and *Myh6*-Cre (~300 bp) sites were indicated.

C, Schematic illustration of transcripts of *Fars2* $^{Mut\text{-}LoxP\ (ML)}$ (up) and *Fars2* $^{cMut\ (R415L)}$ (down). Identification primers were indicated by green (or red) arrows. Red asterisk indicated the variant of c.1244G>T.

D, Transcripts' Sanger sequencing results of *cMut/-* mice heart tissue.

E, Western blot analysis of FARS2, HA-tag, HIS-tag levels in the heart of control and *cMut/-* mice (4 weeks old), GAPDH as lodding control.

F, Representative photograph (up), heart (scare bar=2 mm, middle), longitudinal sections (scare bar=2 mm), Masson's trichrome staining (scare bar=20 μm) from control and *cMut/-* male mice at 4 weeks old.

G, Ratios of HW/BW and HW/TL (n =3 mice per group).

H, Representative M-mode echocardiographic images were shown from control and *cMut/-* male mice at 4 weeks old. End-systole stages were indicated by red lines and end-diastole stages were indicated by yellow lines (up). Echocardiographic quantifications of WT and icKO mice. Shown in the statistical graph were LVEF and LVFS (down).

***P*<0.01. Control indicates *Fars2* $^{ML/-}$, *Myh6*-Cre^{-} mice; *cMut/-*, heterozygous cardiac-specific *Fars2* p.R415L mutant mice; HW/BW, ratio of heart weight to body weight; HW/TL, ratio of heart weight to tibia length; LVEF, left ventricular ejection fraction; LVFS, left ventricular fractional shortening.

Figure S5. Molecular simulation studies of wild type FARS2 and patients derived variants.

A, Structural predictive analysis of WT and FARS2 variants (see Methods). WT was used as negative control and D142Y was used as positive control (known FARS2 variant with damaged aminoacylation and ATP-binding activity) in this study.

B, FARS2-ATP docking studies. Residues interacting with ATP were shown.

C, FARS2-mt-tRNA^{Phe} docking studies. The relative distance of binding residues between FARS2 variants and mt-tRNA^{Phe} was indicated in the upper panel (see methods). The specific binding residues details and those sites which distances were predicted further than 5 Å were summarized in the middle panel. The structural details of the binding residues were indicated in the lower panel.

ATP indicates adenosine triphosphate.

Figure S6. FARS2 is downregulated under heart failure and various cardiomyopathies.

A, The RNA-sequencing data of *FARS2* transcriptional expression was examined in heart tissue of patients suffering from various cardiomyopathies, including hypertrophic cardiomyopathy (GSE180313, GSE160997, GSE1145) and HFrEF (GSE161472).

B, The RNA-sequencing data of *FARS2* transcriptional expression was examined in heart tissue of mice models of heart failure (GSE670) and hypertrophic cardiomyopathy (GSE180720) established by TAC.

HCM indicates hypertrophic cardiomyopathy; TAC, transverse aortic constriction; IDCM, idiopathic dilated cardiomyopathy; PPCM, peripartum cardiomyopathy; ICM, ischemic

cardiomyopathy; VCM, valvular cardiomyopathy; HFrEF, heart failure with reduced ejection fraction; LV, left ventricle; RV, right ventricle; LA, left atrium; RA, right atrium; HF, heart failure; CM, cardiomyocytes; FB, fibroblasts; EC, endothelial cells.

Figure S7. Establishment, identification and phenotypic characteristics of *Fars2* deficiency model *in vivo*

A, Targeting strategy for the inducible cardiac disruption of *Fars2* gene.

B, Genotype analysis of WT and *Fars2* icKO mice. DNA fragments corresponding to the LoxP (393 and 336 bp) and Cre (295 bp) sites were indicated.

C, Effects of cardiac FARS2 ablation on mouse body weight. (left, male; right, female; n =14-30 mice per group; icKO versus WT at the same time).

D, Representative Masson’s trichrome-stained myocardial sections from apex to base heart of sudden death mice (12.9 weeks after icKO) and WT control at the same time. Scare bar = 2 mm.

E, Ratios of HW/BW (n =3–16 mice per group).

F, Ratios of LW/BW (n =3–16 mice per group).

G, Up, schematic illustration showing the two morpholinos (ATG-MO and E3I3-MO) targeted positions (red bars) to generate *fars2* knock-down zebrafish model. Down, RT-PCR analyses of *fars2* transcripts from zebrafish embryos injected with a control MO or the E3I3-MO. Analyses were performed at 2 days post-fertilization.

**P*<0.05; ****P*<0.001; *****P*<0.0001. WT indicates wild type; icKO, inducible cardiac-specific *Fars2* knock-out mice; ratio of HW/BW, heart weight to body weight; LW/BW, ratio

of lung weight to body weight; MO, morpholino.

Figure S8. Pathological analysis of myocardial samples from patient 4 (c.308G>T) and family 1-II-3 (c.1244G>T).

A, Sanger validation of FARS2 variants of two myocardial samples from patient 4 (c.308G>T, up) and family 1-II-3 (c.1244G>T, down).

B, Representative immunohistochemistry images of FARS2 were shown from the heart tissue of control (five healthy donors) and two patients. Scare bar = 100 μm.

C, Representative Masson's trichrome staining of two patients. Lipid droplets were indicated by black arrows. Scare bar = 50 μm.

D, WGA staining of two samples. Cardiomyocyte sectional size analysis of each sample was shown in right (n=200 cells per sample). Mean value of each group was indicated by black arrow. Mean values of the control were derived from 5 healthy donors. Scare bar = 20 μm.

E, Representative TEM images of two samples. Representative cardiomyocyte mitochondria were emphasized by white lines. The "honeycomb" cristae were indicated by white arrows. Upper panel scare bar=2 μm. Lower panel scare bar=200 nm. Quantification of the mitochondrial area (μm^2) of two samples (at least 200 individual mitochondrion) was shown in right panel separately. Mean value of each group was indicated by black arrow. Mean value of the control group was derived from supplemental figure VII-C of the reference [17].

F, Representative immunofluorescence images of Parkin (red) and VDAC1 (green) were shown from the samples. Scare bar=20 μm.

G, Representative immunofluorescence images of α-actin (red) and SQSTM1/p62 (green)

were shown from the samples. Scare bar=20 μm.

WGA indicates wheat germ agglutinin; TEM, transmission electron microscopy.

Figure S9. FARS2 deficiency causes myocardial and mitochondrial dysfunctions in mice

A, Echocardiographic quantifications of WT and *Fars2* icKO mice. Shown in the statistical graph were IVS;D and IVS;S (left panel), LVID;D and LV vol;D (right panel).

B, Quantification of the relative ATP content in icKO mice (n=3).

C, Representative images of DHE staining in different groups. Scare bar= 20 μm.

D, The associated statistics from **C** (n=3).

**P*<0.05; ***P*<0.01; ****P*<0.001; *****P*<0.0001. WT indicates wild type; icKO, inducible cardiac-specific *Fars2* knock-out mice; IVS;D, interventricular septal thickness at end-diastole; IVS;S, interventricular septal thickness at end-systole; LVID;D, left ventricle diastolic internal diameters; LV vol;D, left ventricle volume at end-diastole; ATP, adenosine triphosphate; DHE, dihydroethidium.

Figure S10. FARS2 deficiency causes mitochondrial dysfunctions in NRVMs

A, Schematic diagram of experimental protocol in *Fars2* knock-down NRVMs.

B, Relative *Fars2* mRNA expression levels of *Fars2* knock-down NRVMs at different time (n=3).

C, Down, western blot of FARS2 of *Fars2* knock-down NRVM. The statistical analysis was shown in upper panel (n=3).

D, Quantification of the relative ATP content of *Fars2* knock-down NRVMs (n=6).

E, Quantification of relative DCF fluorescence intensity of *Fars2* knock-down NRVMs from **Figure 3G** (n=3).

F, Representative FACS images in *Fars2* knock-down NRVMs by JC-1 staining.

G, Quantification of relative JC-1 aggregates/monomer ratio from **F** (n=3).

H, The relative levels of total NAD, NAD^+, NADH, and ratio of NAD^+/NADH in shCtrl or sh-*Fars2* NRVMs at 5 days after sh-*Fars2* or shCtrl treatment.

I, Basal respiration, ATP production, proton leak, maximal respiration, and spare respiratory capacity in shCtrl or sh-*Fars2* NRVMs (n=7).

J, Glycolysis and glycolytic capacity in shCtrl or sh-*Fars2* NRVMs (n=5-6).

***P*<0.01; ****P*<0.001; *****P*<0.0001. NRVMs indicates neonatal rat ventricular myocytes; ATP, adenosine triphosphate; DCF, 2’,7’-dichlorofluorescein; FACS, fluorescence activated cell sorting; NAD, nicotinamide adenine dinucleotid.

Figure S11. *Fars2* deficiency causes increase of mitochondrion-derived ROS production and decrease of ΔΨm in NRVMs

A, Representative images of Mito-Tracker Green (green) and TMRE (red) in control or *Fars2* knock-down NRVMs; the mitochondrial deporlarization was emphasized by white arrow. Scare bar=10 μm.

B, Representative images of Mito-Tracker Green (green) and MitoSOX (red) in control or *Fars2* knock-down NRVMs; the production of mitochondrion-derived ROS was emphasized by white arrow. Scare bar=10 μm.

TMRE indicates tetramethylrhodamine ethyl ester perchlorate; NRVMs, neonatal rat

ventricular myocytes; ROS, reactive oxygen species.

Figure S12. RNA-seq analysis in icKO hearts.

A, The bar plot of RNA-seq analysis. The blue bar indicated the downregulated genes, while the red indicated the upregulated.

B, The volcano plot in RNA-seq analysis. The blue dot indicated the downregulated genes, while the red indicated the upregulated.

C, KEGG enrichment analysis of icKO mice from RNA-seq.

KEGG indicates Kyoto Encyclopedia of Genes and Genomes; icKO, inducible cardiac-specific *Fars2* knock-out mice.

Figure S13. Impaired mitochondrial homeostasis and protein synthesis in icKO hearts and *Fars2* knock-down NRVMs.

A, Western blots of mtDNA coding proteins in WT and icKO mice.

B, Quantification of mtDNA coding proteins from **A** (n=3 male mice per group).

C, Western blots of OXPHOs complexes of control and *Fars2* knock-down NRVMs.

D, Quantification of the OXPHOs complexes from **D** (n=3).

E, Quantification of OXPHOs complexes activity in heart of WT and icKO mice (10 weeks after icKO) (n=4 male mice per group).

F, mtDNA-CN (mtDNA/nDNA) was detected by qRT-PCR of WT and icKO mice (n=3-9 mice per group).

G, mtDNA-CN (mtDNA/nDNA) was detected by qRT-PCR of NRVMs (n=6 per group).

*P<0.05; **P<0.01; ***P<0.001; ****P<0.0001. mtDNA indicates mitochondrial DNA; WT, wild type; icKO, inducible cardiac-specific *Fars2* knock-out mice; OXPHOs, oxidative phosphorylation; NRVMs, neonatal rat ventricular myocytes; mtDNA-CN, mitochondrial DNA copy number.

Figure S14. FARS2 deficiency causes MQC system disruption.

A, Relative mRNA levels of key genes in autophagy and mitochondrial dynamics in mice 3 weeks or 10 weeks after icKO (n=3).

B, Western blots of proteins in mitochondrial dynamics of whole cell lysate of WT and icKO mice.

C, Quantification of relative protein expression in **B** (n=3).

D, Western blots of proteins in mitochondrial dynamics of mitochondrial lysate of WT and icKO mice.

E, Quantification of relative protein expression in **D** (n=3).

F, Up, western blots of PGC-1α in WT and icKO mice. Down, the quantification of PGC-1α (n=3).

G, Relative mRNA levels of key genes in mito-genesis in mice 3 weeks or 10 weeks after icKO (n=3).

*P<0.05; **P<0.01; ***P<0.001; ****P<0.0001. WT indicates wild type; icKO, inducible cardiac-specific *Fars2* knock-out mice.

Figure S15. The continuous increase in autophagy flow caused by FARS2 deficiency was

an excessive stress in MQC and resulted in unbalanced mitochondrial mass.

A, Relative LC3 levels of *Fars2* knock-down NRVMs at different time.

B, Quantification of the LC3 from **A** (n=3).

C, Western of key proteins of autophagy after BafA1 treatment.

D-E, Quantification from C (n=3).

F, Representative images of NRVMs after mGFP-RFP-LC3 adenovirus transfection and *Fars2* knock-down. Autophagagosomes were indicated by yellow dots and autolysosomes were indicated by red dots.

G, Quantification of autophagy flow changes from **F**.

H, Representative images of TEM images in control or *Fars2* knock-down NRVMs; the fission of mitochondrion was emphasized by red arrow. Scare bar=500 nm.

I, Decease of mitochondrial mass in *Fars2* knock-down NRVMs at 3, 5, 7 days by mito-Tracker Green staining.

J, Quantification of mitochondrial mass changes from **J** (n=3).

**P*<0.05; ***P*<0.01; ****P*<0.001; *****P*<0.0001. NRVMs indicates neonatal rat ventricular myocytes; TEM, transmission electron microscopy.

Figure S16. Mitochondrial fission and autophagy inhibitors attenuate FARS2 deficiency-induced mitochondrial dyshomeostasis in NRVMs

A, Schematic diagram of experimental protocol in *Fars2* knock-down NRVMs with 3-MA or Mdivi-1 treatment.

B, Western blots of proteins in autophagy in *Fars2* knock-down NRVMs after 3-MA or

Mdivi-1 treatment.

C, Quantification of relative protein expression in **B** (n=3).

D, Western blots of proteins in mitochondrial dynamics in *Fars2* knock-down NRVMs after 3-MA or Mdivi-1 treatment.

E, Quantification of relative protein expression in **D** (n=3).

F, Representative immunofluorescence images of LC3 (red) and TOMM20 (green) were shown from NRVMs after 3-MA or Mdivi-1 treatment. Scare bar= 20 μm.

G, Representative immunofluorescence images of DRP1 (red) and TOMM20 (green) were shown from NRVMs after 3-MA or Mdivi-1 treatment. Scare bar= 20 μm.

H, Quantification of mitochondrial mass changes from **Figure 6B** (n=3).

I, Quantification of relative DCF fluorescence intensity of *Fars2* knock-down NRVMs from **Figure 6E** (n=3).

*P<0.05; **P<0.01; ***P<0.001; ****P<0.0001. NRVMs indicates neonatal rat ventricular myocytes; DCF, 2',7'-dichlorofluorescein.

Figure S17. Mitochondrial dynamics intervention attenuates *Fars2* deficiency-induced cardiac hypertrophy in mice.

A, Western blots of FARS2, DRP1 and MFN1 of different group in mice 10 weeks after icKO (n=3).

B, Ratios of HW/BW (n =6).

***P<0.001; ****P<0.0001. icKO indicates inducible cardiac-specific *Fars2* knock-out mice; HW/BW, ratio of heart weight to body weight.

Videos.

Video 1. The physical activity status of WT and *Fars2* icKO mice (12 weeks after icKO).

Video 2-4. The phenotypic characteristics of control-MO (Video 1), *fars2*-ATG-MO (Video 2) and *fars2*-EI3I-MO (Video 3) injected morphants at 50-hpf.

Figure S1

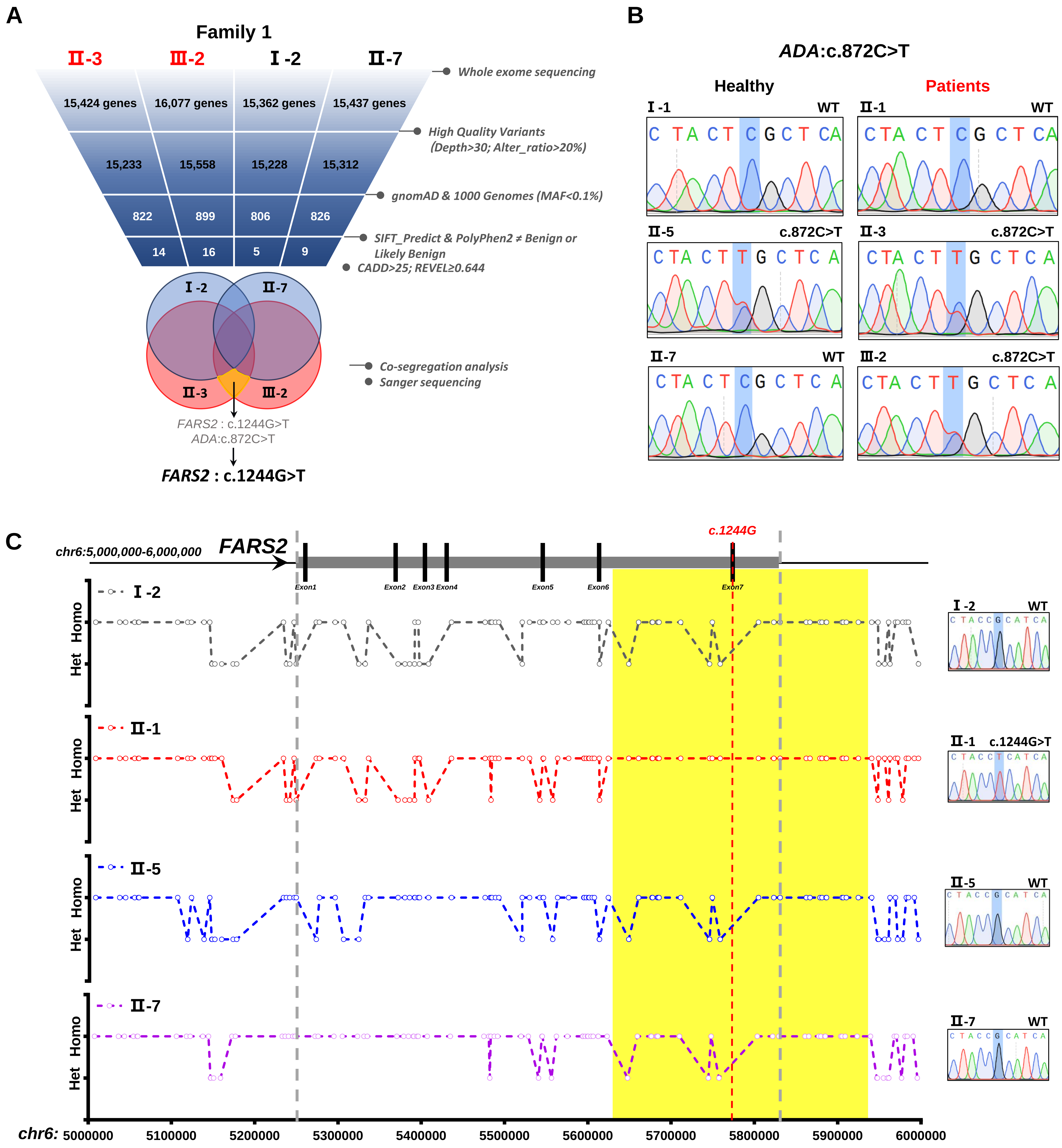

A
Family 1
Ⅱ-3
Ⅲ-2
Ⅰ-2
Ⅱ-7
Whole exome sequencing
15,424 genes
16,077 genes
15,362 genes
15,437 genes
High Quality Variants (Depth>30; Alter_ratio>20%)
15,233
15,558
15,228
15,312
gnomAD & 1000 Genomes (MAF<0.1%)
822
899
806
826
SIFT_Predict & PolyPhen2 ≠ Benign or Likely Benign
CADD>25; REVEL≥0.644
14
16
5
9
Co-segregation analysis
Sanger sequencing
FARS2 : c.1244G>T
ADA:c.872C>T
FARS2 : c.1244G>T
B
ADA:c.872C>T
Healthy
Patients
Ⅰ-1 WT
Ⅱ-1 WT
Ⅱ-5 c.872C>T
Ⅱ-3 c.872C>T
Ⅱ-7 WT
Ⅲ-2 c.872C>T
C
chr6:5,000,000-6,000,000
FARS2
c.1244G
Exon1
Exon2
Exon3
Exon4
Exon5
Exon6
Exon7
Ⅰ-2
Ⅱ-1
Ⅱ-5
Ⅱ-7
Homo
Het
Ⅰ-2 WT
Ⅱ-1 c.1244G>T
Ⅱ-5 WT
Ⅱ-7 WT
chr6:
5000000
5100000
5200000
5300000
5400000
5500000
5600000
5700000
5800000
5900000
6000000

Figure S2

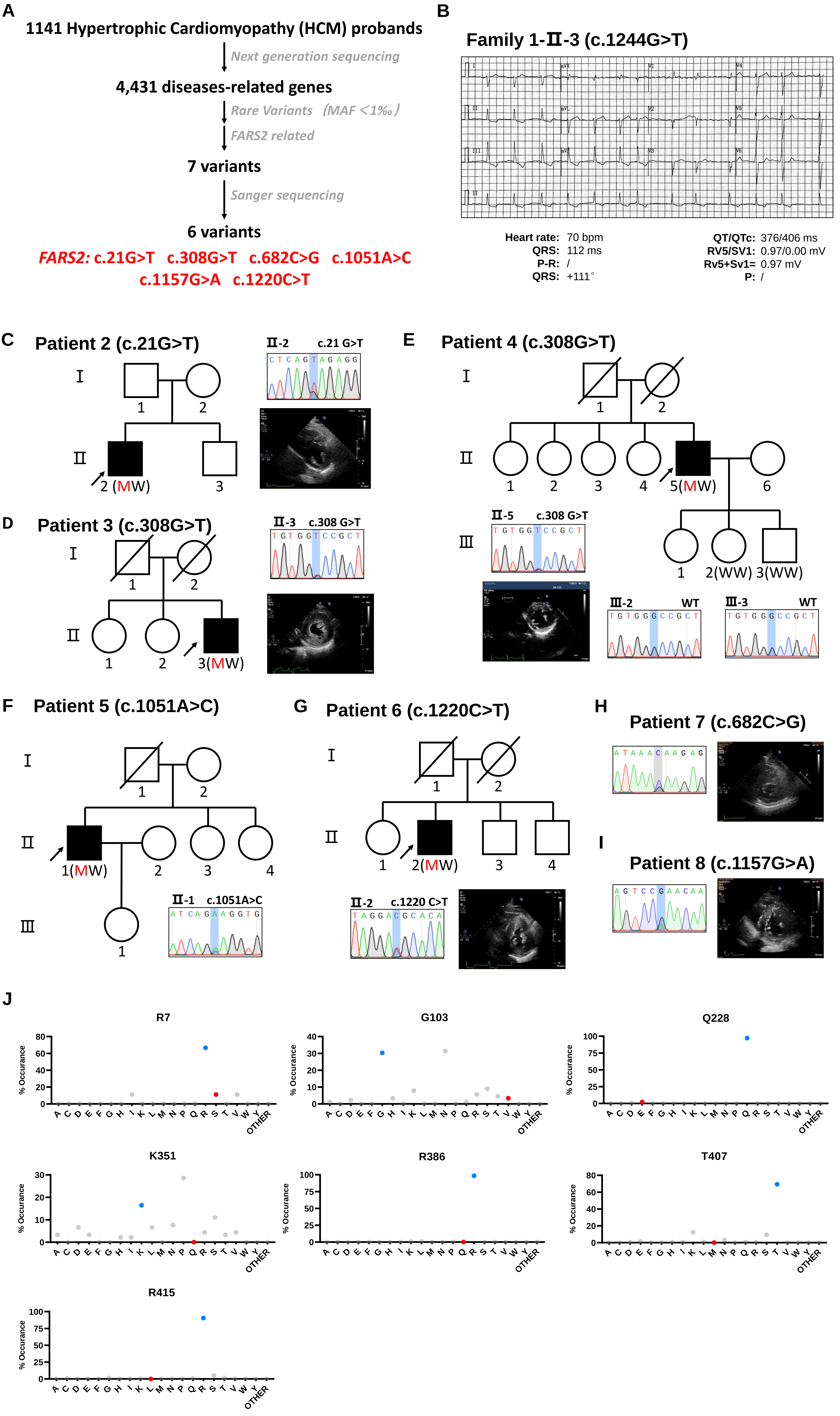

A
1141 Hypertrophic Cardiomyopathy (HCM) probands
Next generation sequencing
4,431 diseases-related genes
Rare Variants (MAF <1‰)
FARS2 related
7 variants
Sanger sequencing
6 variants
FARS2: c.21G>T c.308G>T c.682C>G c.1051A>C
c.1157G>A c.1220C>T
B
Family 1-Ⅱ-3 (c.1244G>T)
Heart rate: 70 bpm
QRS: 112 ms
P-R: /
QRS: +111°
QT/QTc: 376/406 ms
RV5/SV1: 0.97/0.00 mV
Rv5+Sv1= 0.97 mV
P: /
C
Patient 2 (c.21G>T)
Ⅱ-2 c.21 G>T
2 (MW)
D
Patient 3 (c.308G>T)
Ⅱ-3 c.308 G>T
3(MW)
E
Patient 4 (c.308G>T)
5(MW)
Ⅱ-5 c.308 G>T
2(WW) 3(WW)
Ⅲ-2 WT
Ⅲ-3 WT
F
Patient 5 (c.1051A>C)
1(MW)
Ⅱ-1 c.1051A>C
G
Patient 6 (c.1220C>T)
2(MW)
Ⅱ-2 c.1220 C>T
H
Patient 7 (c.682C>G)
I
Patient 8 (c.1157G>A)
J
R7
G103
Q228
K351
R386
T407
R415
% Occurance
OTHER

Figure S3

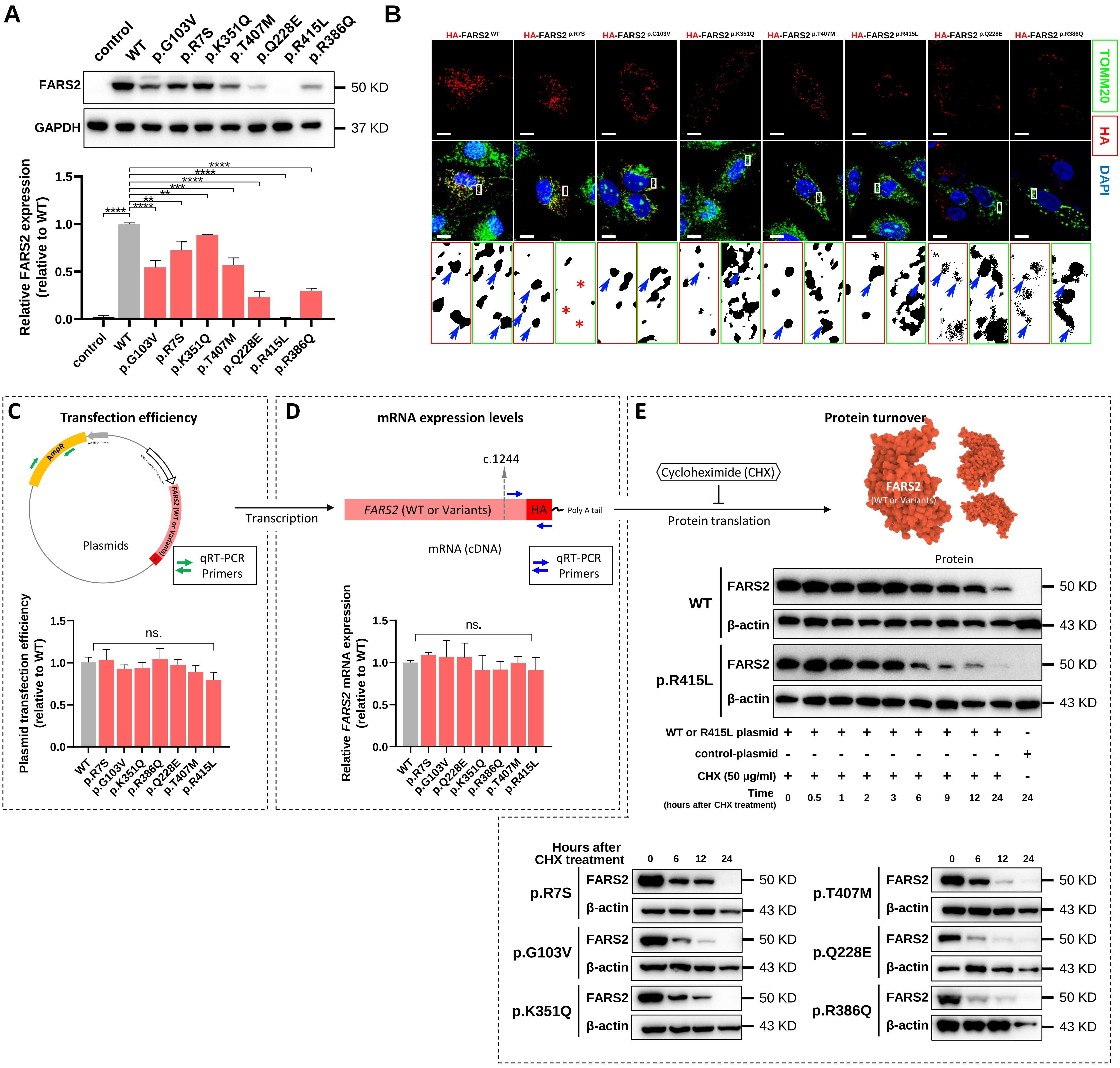
A
B
C
D
E
Transfection efficiency
mRNA expression levels
Protein turnover
Cycloheximide (CHX)
Protein translation
Transcription
qRT-PCR Primers
Plasmids
mRNA (cDNA)
c.1244
Poly A tail
ns.
Relative FARS2 expression (relative to WT)
Plasmid transfection efficiency (relative to WT)
Relative FARS2 mRNA expression (relative to WT)
TOMM20
HA
DAPI
50 KD
37 KD
43 KD
WT or R415L plasmid
control-plasmid
CHX (50 μg/ml)
Time (hours after CHX treatment)
Hours after CHX treatment

Figure S4

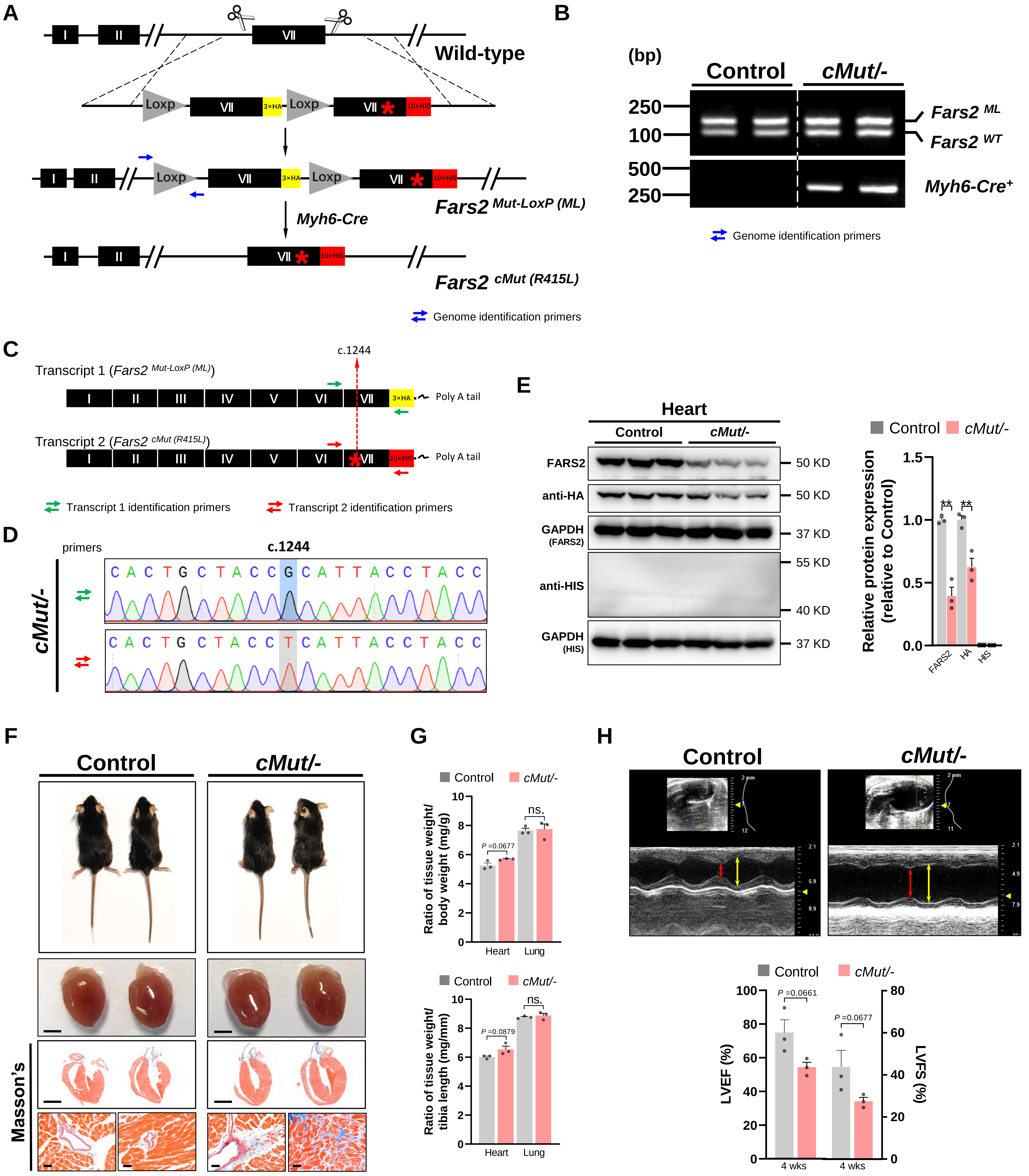

A
Wild-type
Loxp
3×HA
10×HIS
Fars2 Mut-LoxP (ML)
Myh6-Cre
Fars2 cMut (R415L)
Genome identification primers
B
(bp)
Control
cMut/-
250
100
500
250
Fars2 ML
Fars2 WT
Myh6-Cre+
Genome identification primers
C
c.1244
Transcript 1 (Fars2 Mut-LoxP (ML))
Poly A tail
Transcript 2 (Fars2 cMut (R415L))
Transcript 1 identification primers
Transcript 2 identification primers
D
primers
c.1244
cMut/-
C A C T G C T A C C G C A T T A C C T A C C
C A C T G C T A C C T C A T T A C C T A C C
E
Heart
Control
cMut/-
FARS2
anti-HA
GAPDH (FARS2)
anti-HIS
GAPDH (HIS)
50 KD
50 KD
37 KD
55 KD
40 KD
37 KD
Relative protein expression (relative to Control)
FARS2
HA
HIS
F
Control
cMut/-
Masson's
G
Ratio of tissue weight/ body weight (mg/g)
P =0.0677
ns.
Heart
Lung
Ratio of tissue weight/ tibia length (mg/mm)
P =0.0879
H
Control
cMut/-
LVEF (%)
LVFS (%)
P =0.0661
P =0.0677
4 wks

Figure S5

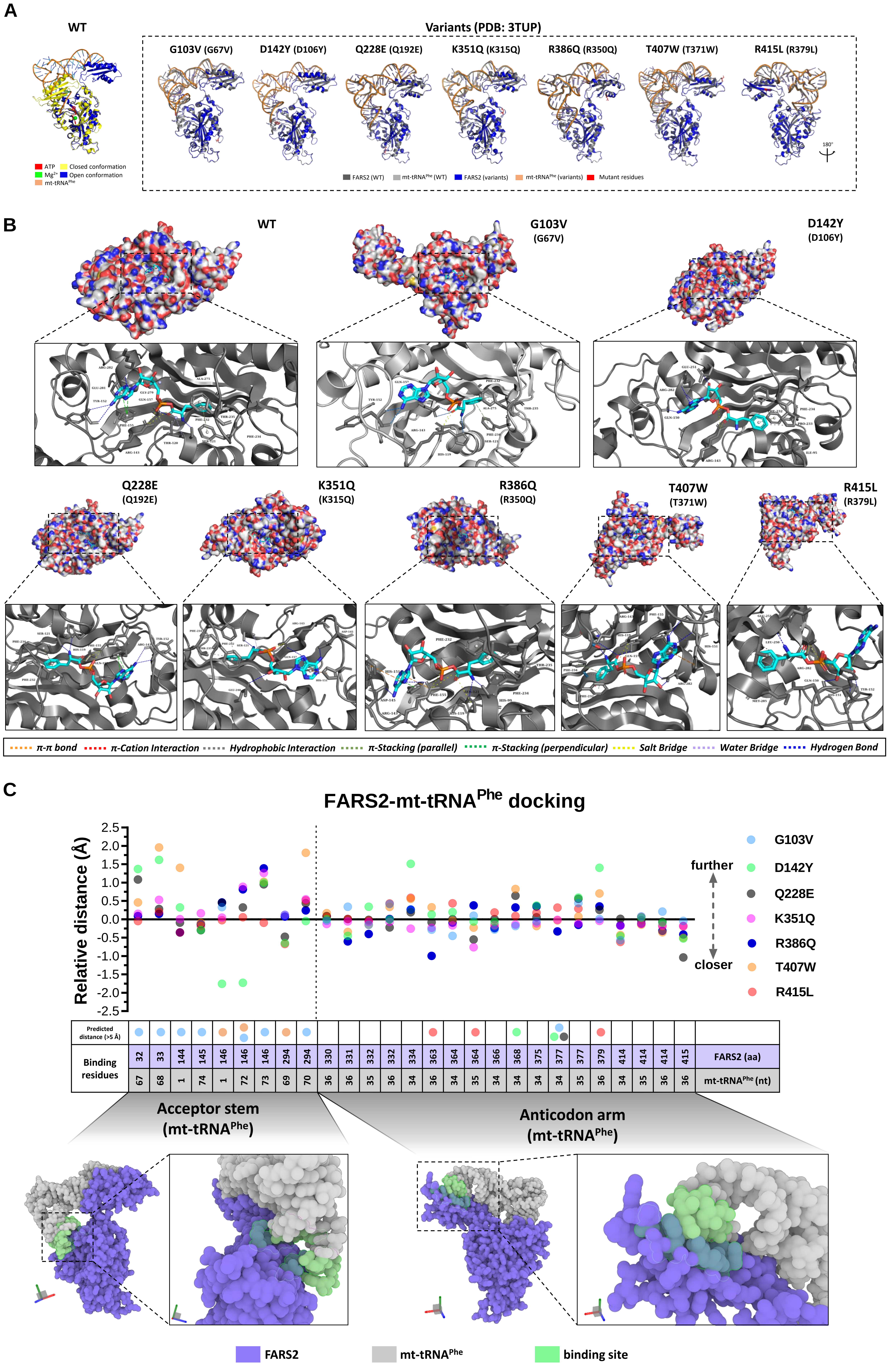

A
WT
Variants (PDB: 3TUP)
G103V (G67V)
D142Y (D106Y)
Q228E (Q192E)
K351Q (K315Q)
R386Q (R350Q)
T407W (T371W)
R415L (R379L)
ATP
Closed conformation
Mg2+
Open conformation
mt-tRNAPhe
FARS2 (WT)
mt-tRNAPhe (WT)
FARS2 (variants)
mt-tRNAPhe (variants)
Mutant residues
B
WT
G103V (G67V)
D142Y (D106Y)
Q228E (Q192E)
K351Q (K315Q)
R386Q (R350Q)
T407W (T371W)
R415L (R379L)
π-π bond
π-Cation Interaction
Hydrophobic Interaction
π-Stacking (parallel)
π-Stacking (perpendicular)
Salt Bridge
Water Bridge
Hydrogen Bond
C
FARS2-mt-tRNAPhe docking
Relative distance (Å)
further
closer
G103V
D142Y
Q228E
K351Q
R386Q
T407W
R415L
Predicted distance (>5 Å)
Binding residues
FARS2 (aa): 32 33 144 145 146 146 146 294 294 330 331 332 332 334 363 364 364 366 368 375 377 377 379 414 414 414 415
mt-tRNAPhe (nt): 67 68 1 74 1 72 73 69 70 36 36 35 36 34 36 34 35 34 34 34 34 35 36 34 35 36 36
Acceptor stem (mt-tRNAPhe)
Anticodon arm (mt-tRNAPhe)
FARS2
mt-tRNAPhe
binding site

Figure S6

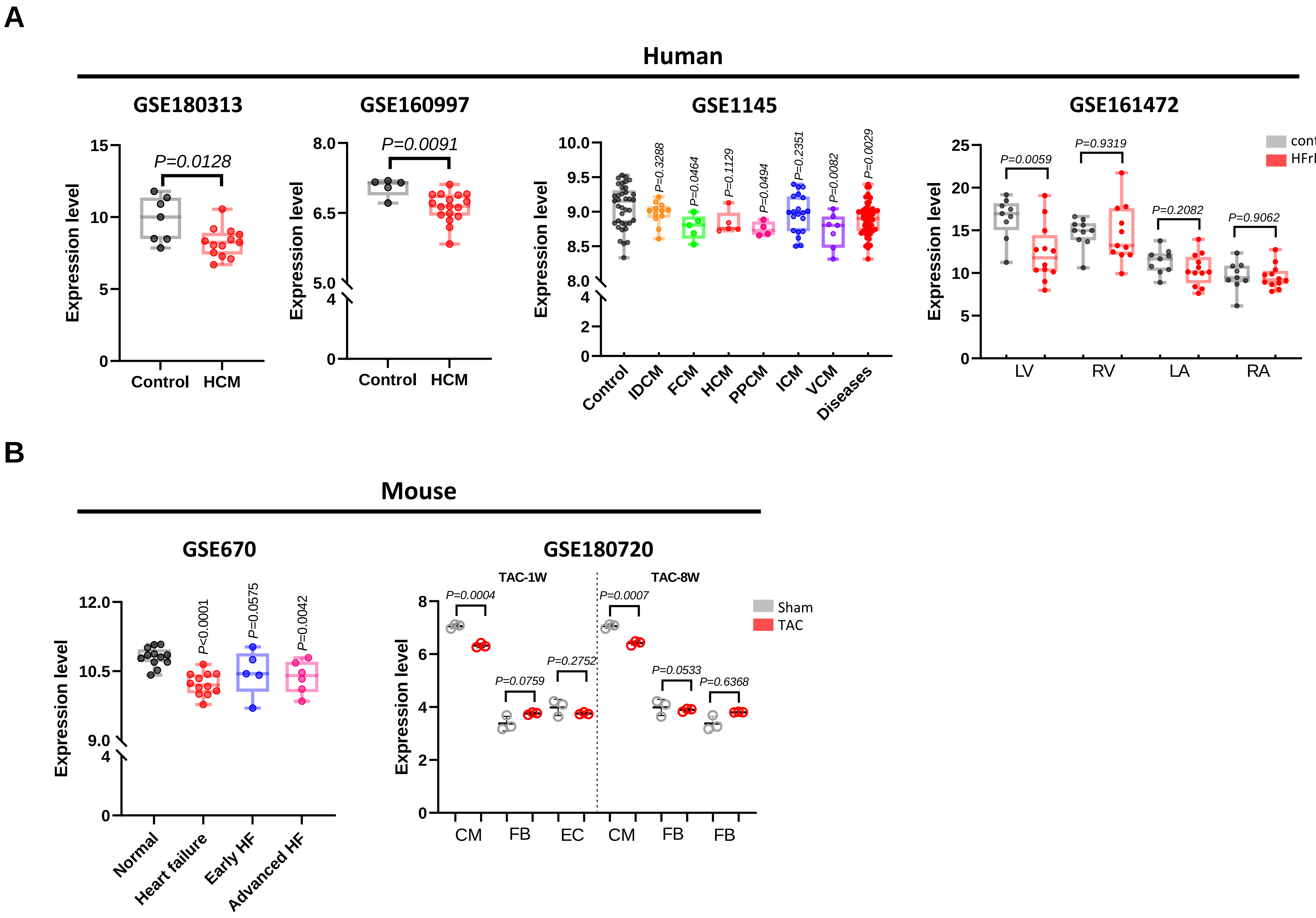
A
Human
GSE180313
P=0.0128
Expression level
Control
HCM
GSE160997
P=0.0091
GSE1145
Control
IDCM
FCM
HCM
PPCM
ICM
VCM
Diseases
P=0.3288
P=0.0464
P=0.1129
P=0.0494
P=0.2351
P=0.0082
P=0.0029
GSE161472
control
HFrEF
P=0.0059
P=0.9319
P=0.2082
P=0.9062
LV
RV
LA
RA
B
Mouse
GSE670
Normal
Heart failure
Early HF
Advanced HF
P<0.0001
P=0.0575
P=0.0042
GSE180720
TAC-1W
TAC-8W
Sham
TAC
P=0.0004
P=0.0759
P=0.2752
P=0.0007
P=0.0533
P=0.6368
CM
FB
EC

Figure S7

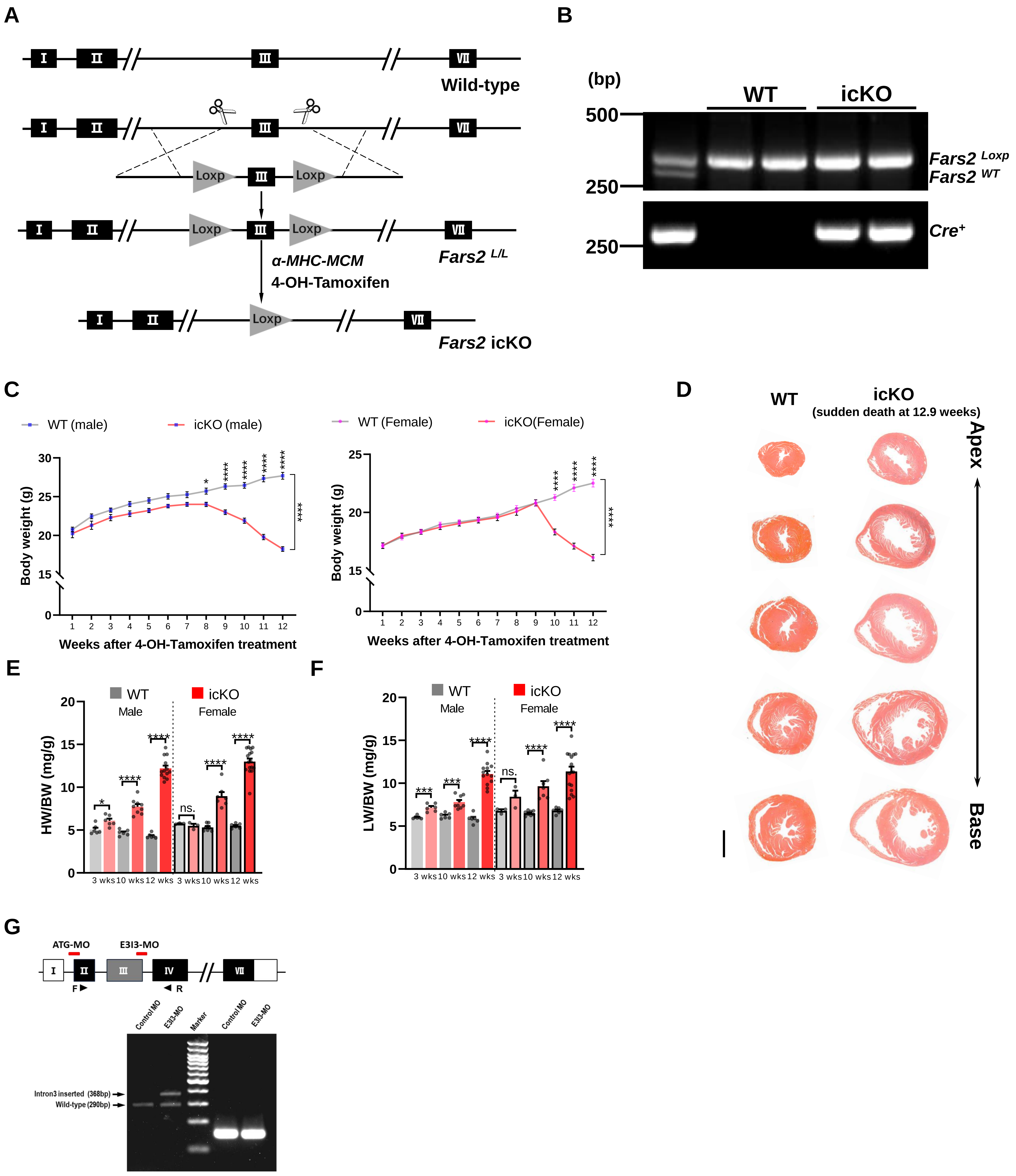
A
Wild-type
Loxp
α-MHC-MCM
4-OH-Tamoxifen
Fars2 L/L
Fars2 icKO
B
(bp)
WT
icKO
500
250
250
Fars2 Loxp
Fars2 WT
Cre+
C
WT (male)
icKO (male)
WT (Female)
icKO(Female)
Body weight (g)
Weeks after 4-OH-Tamoxifen treatment
D
WT
icKO
(sudden death at 12.9 weeks)
Apex
Base
E
WT
icKO
Male
Female
HW/BW (mg/g)
3 wks 10 wks 12 wks 3 wks 10 wks 12 wks
ns.
F
WT
icKO
Male
Female
LW/BW (mg/g)
3 wks 10 wks 12 wks 3 wks 10 wks 12 wks
ns.
G
ATG-MO
E3I3-MO
F
R
Control MO
E3I3-MO
Marker
Control MO
E3I3-MO
Intron3 inserted (368bp)
Wild-type (290bp)

Figure S8

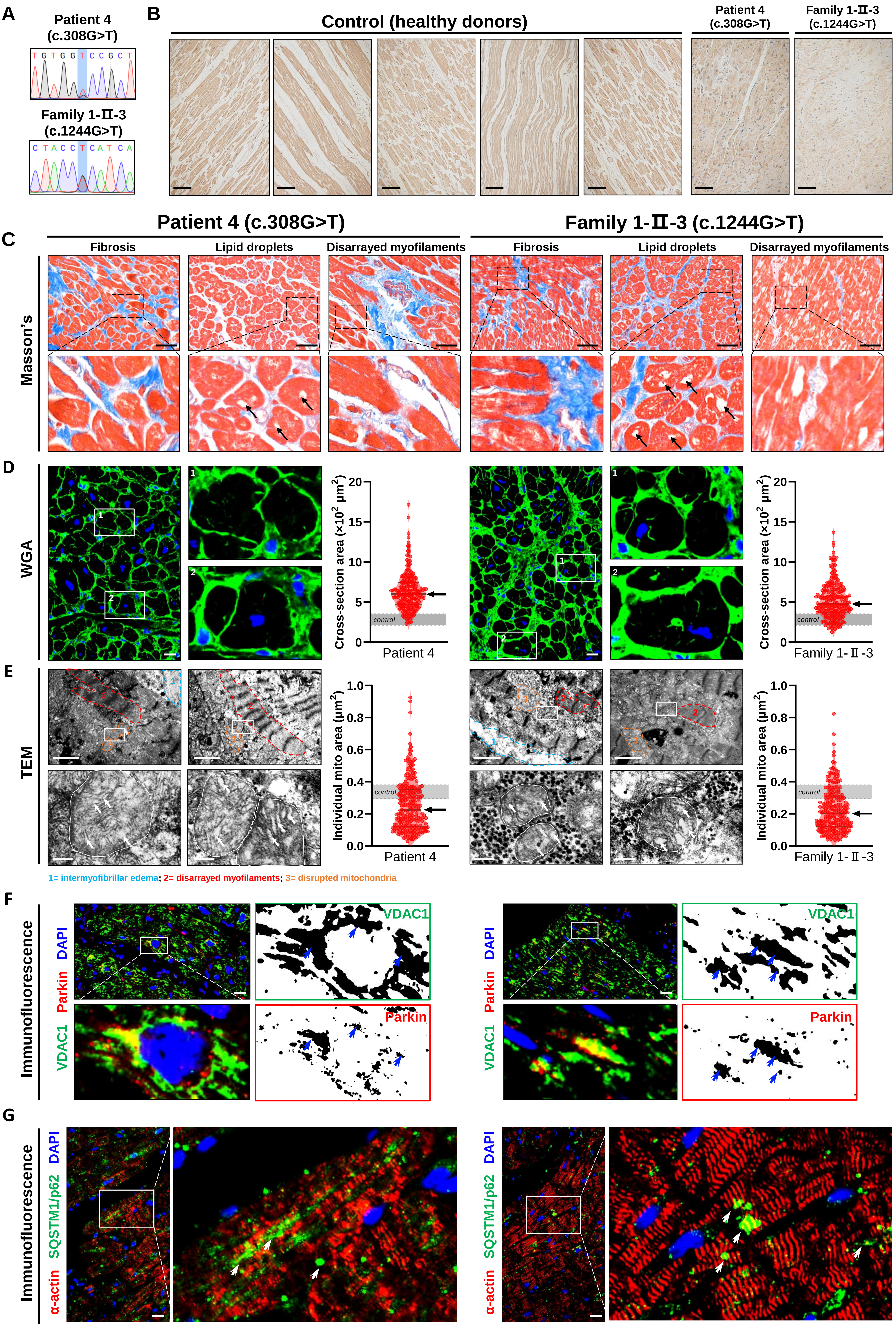
A
Patient 4
(c.308G>T)
Family 1-Ⅱ-3
(c.1244G>T)
B
Control (healthy donors)
Patient 4
(c.308G>T)
Family 1-Ⅱ-3
(c.1244G>T)
C
Patient 4 (c.308G>T)
Family 1-Ⅱ-3 (c.1244G>T)
Fibrosis
Lipid droplets
Disarrayed myofilaments
Masson's
D
WGA
Cross-section area (×10² μm²)
control
Patient 4
Family 1- Ⅱ -3
E
TEM
Individual mito area (μm²)
1= intermyofibrillar edema; 2= disarrayed myofilaments; 3= disrupted mitochondria
F
Immunofluorescence
DAPI
Parkin
VDAC1
G
SQSTM1/p62
α-actin

Figure S9

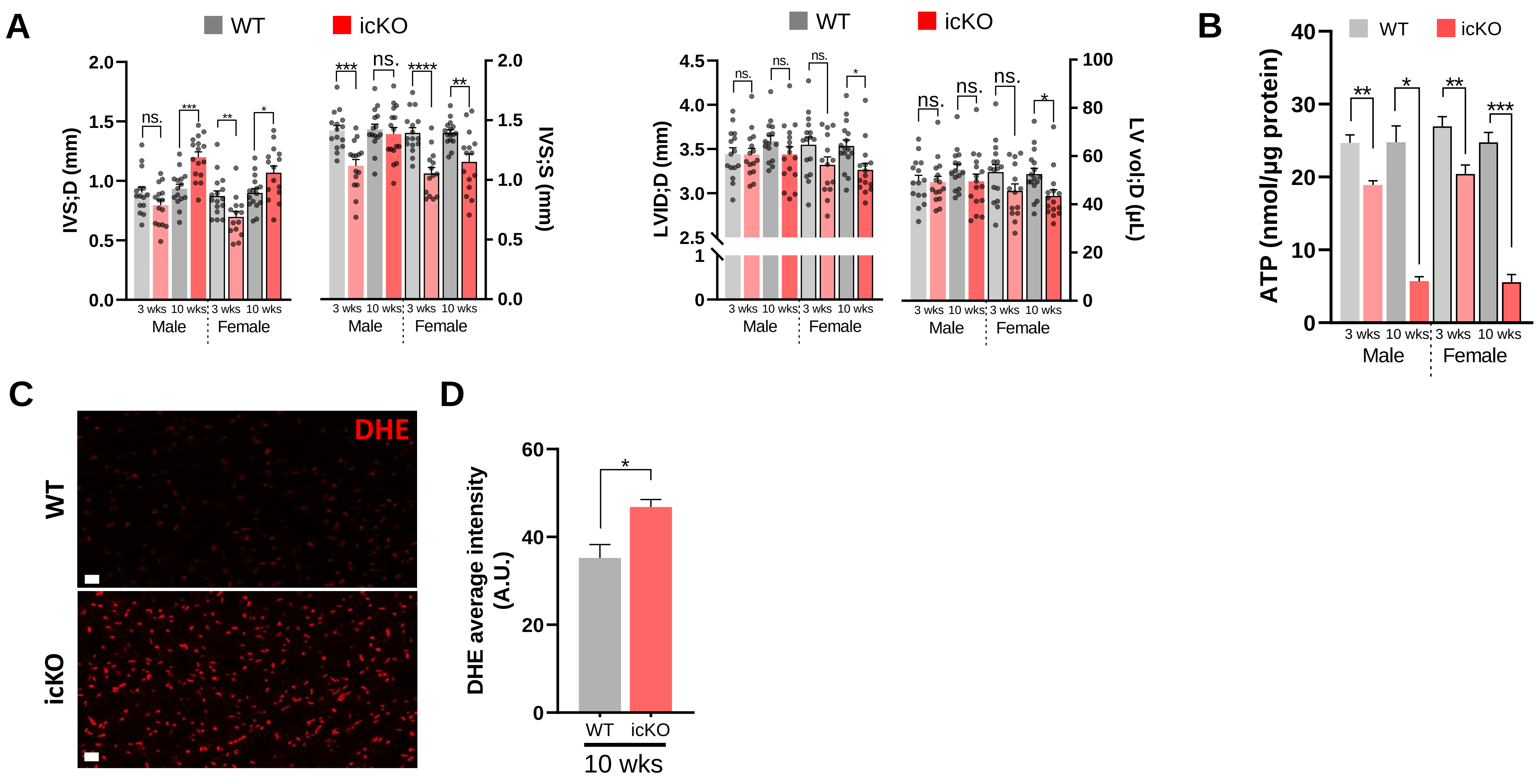

A
WT
icKO
IVS;D (mm)
IVS;S (mm)
LVID;D (mm)
LV vol;D (µL)
3 wks
10 wks
Male
Female
ns.
B
ATP (nmol/µg protein)
C
DHE
WT
icKO
D
DHE average intensity (A.U.)
10 wks

Figure S10

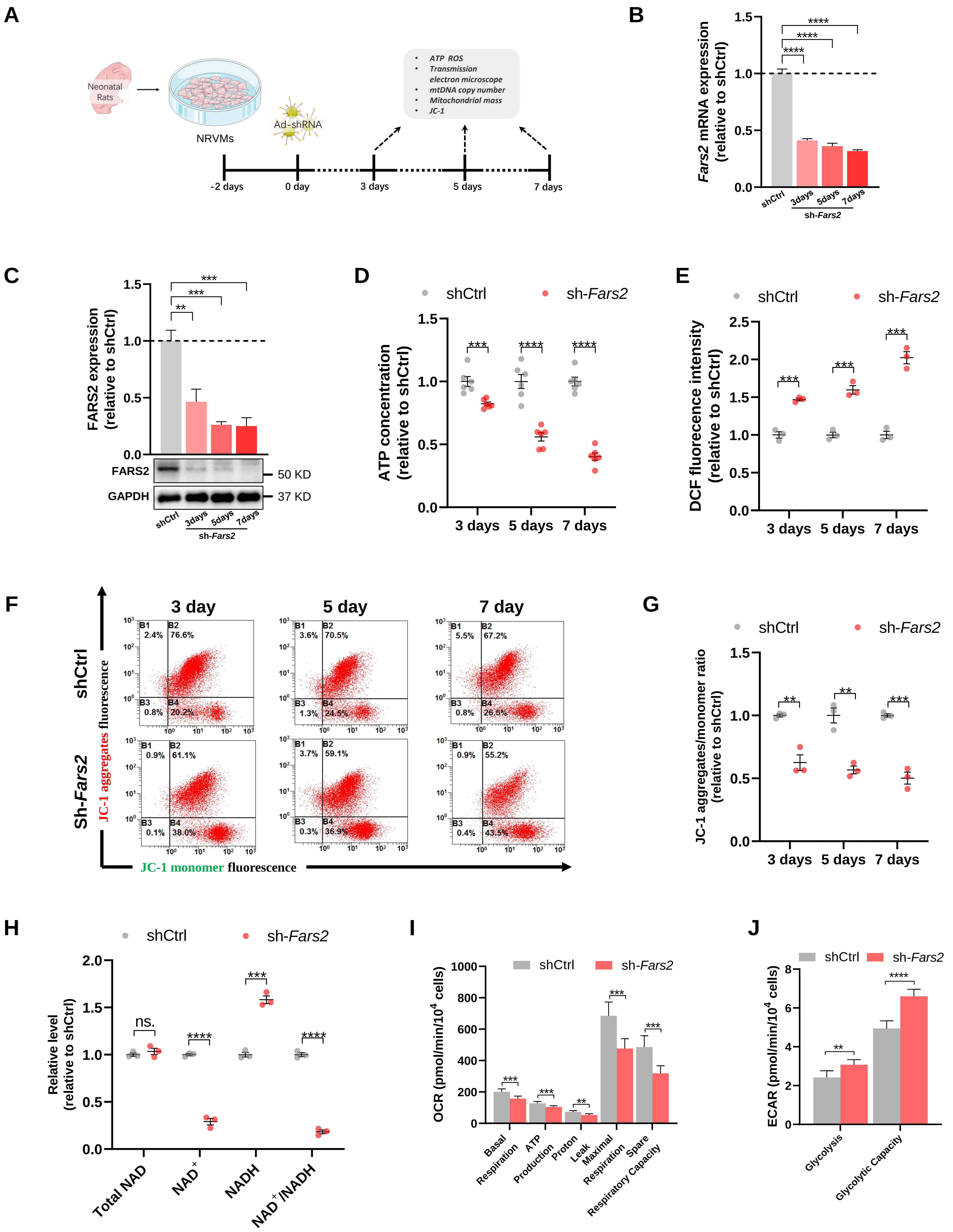

A
Neonatal Rats
NRVMs
Ad-shRNA
ATP ROS
Transmission electron microscope
mtDNA copy number
Mitochondrial mass
JC-1
-2 days
0 day
3 days
5 days
7 days
B
Fars2 mRNA expression (relative to shCtrl)
shCtrl
3days
5days
7days
sh-Fars2
C
FARS2 expression (relative to shCtrl)
FARS2
GAPDH
50 KD
37 KD
D
shCtrl
sh-Fars2
ATP concentration (relative to shCtrl)
3 days
5 days
7 days
E
DCF fluorescence intensity (relative to shCtrl)
F
3 day
5 day
7 day
shCtrl
Sh-Fars2
JC-1 aggregates fluorescence
JC-1 monomer fluorescence
G
JC-1 aggregates/monomer ratio (relative to shCtrl)
H
Relative level (relative to shCtrl)
Total NAD
NAD+
NADH
NAD+/NADH
ns.
I
OCR (pmol/min/10^4 cells)
Basal Respiration
ATP Production
Proton Leak
Maximal Respiration
Spare Respiratory Capacity
J
ECAR (pmol/min/10^4 cells)
Glycolysis
Glycolytic Capacity

Figure S11

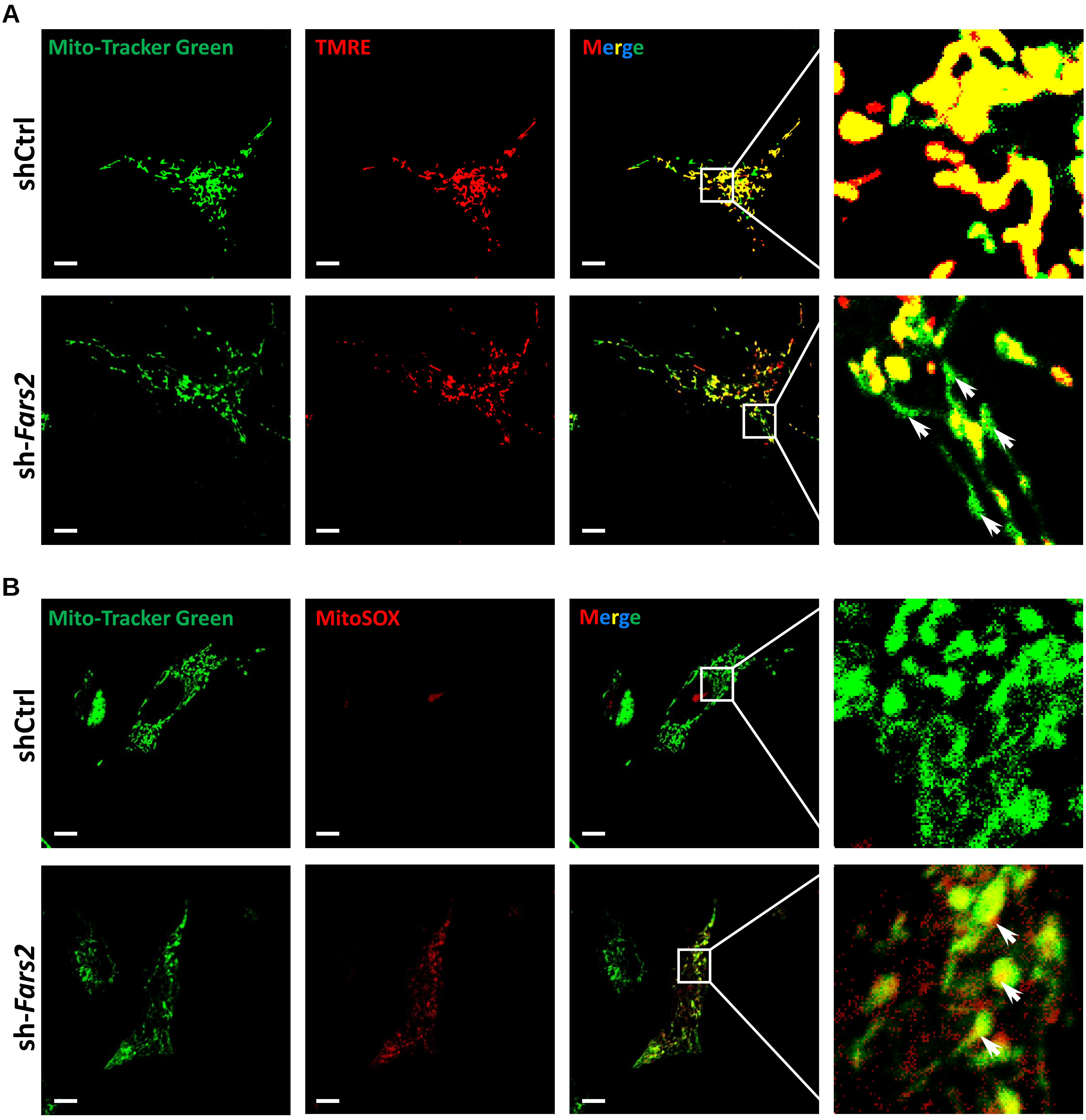
A
Mito-Tracker Green
TMRE
Merge
shCtrl
sh-Fars2
B
Mito-Tracker Green
MitoSOX
Merge
shCtrl
sh-Fars2

Figure S12

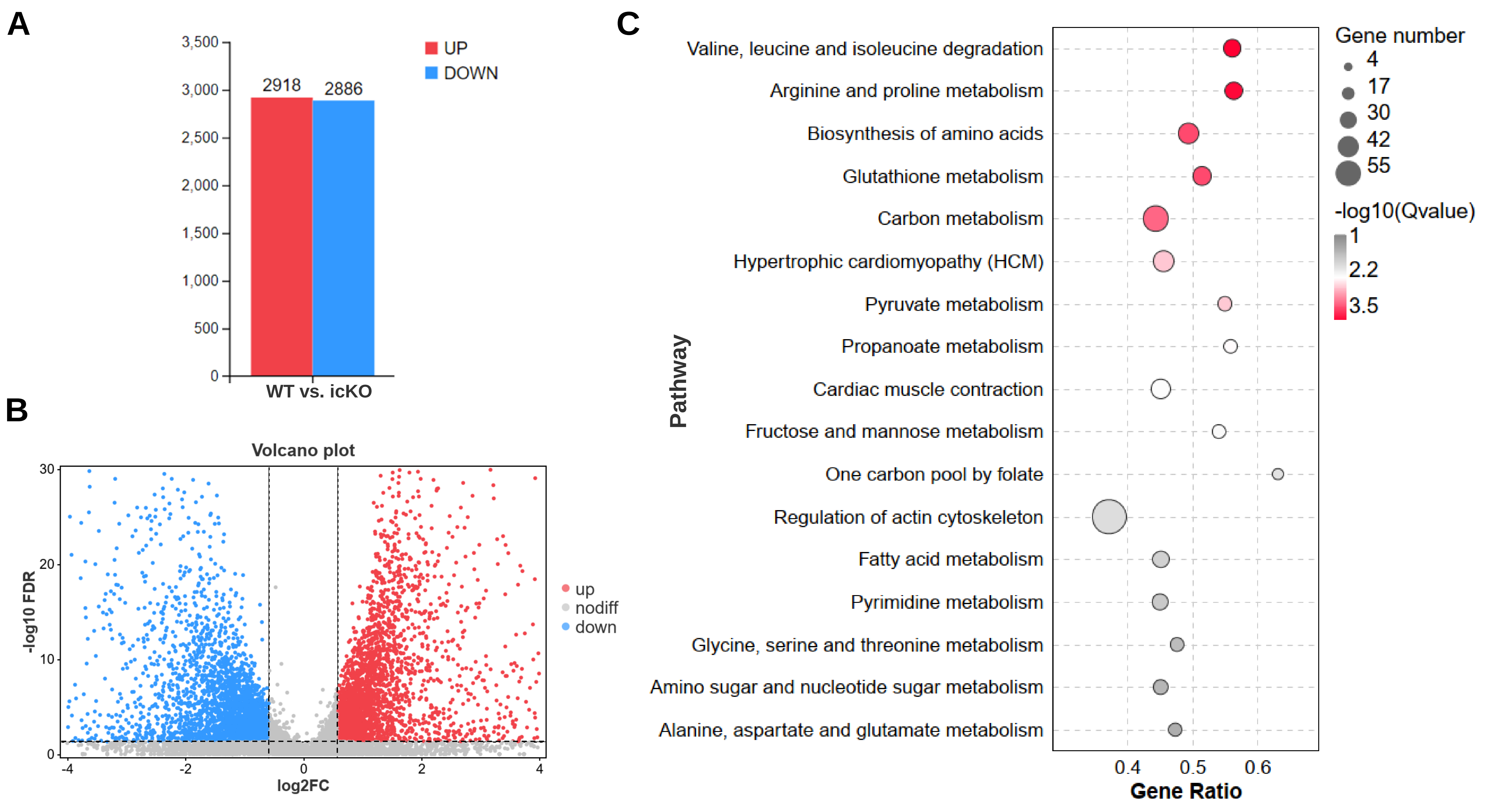
A
3,500
3,000
2,500
2,000
1,500
1,000
500
0
2918
2886
UP
DOWN
WT vs. icKO
B
Volcano plot
-log10 FDR
log2FC
up
nodiff
down
C
Pathway
Valine, leucine and isoleucine degradation
Arginine and proline metabolism
Biosynthesis of amino acids
Glutathione metabolism
Carbon metabolism
Hypertrophic cardiomyopathy (HCM)
Pyruvate metabolism
Propanoate metabolism
Cardiac muscle contraction
Fructose and mannose metabolism
One carbon pool by folate
Regulation of actin cytoskeleton
Fatty acid metabolism
Pyrimidine metabolism
Glycine, serine and threonine metabolism
Amino sugar and nucleotide sugar metabolism
Alanine, aspartate and glutamate metabolism
Gene Ratio
Gene number
4
17
30
42
55
-log10(Qvalue)
1
2.2
3.5

Figure S13

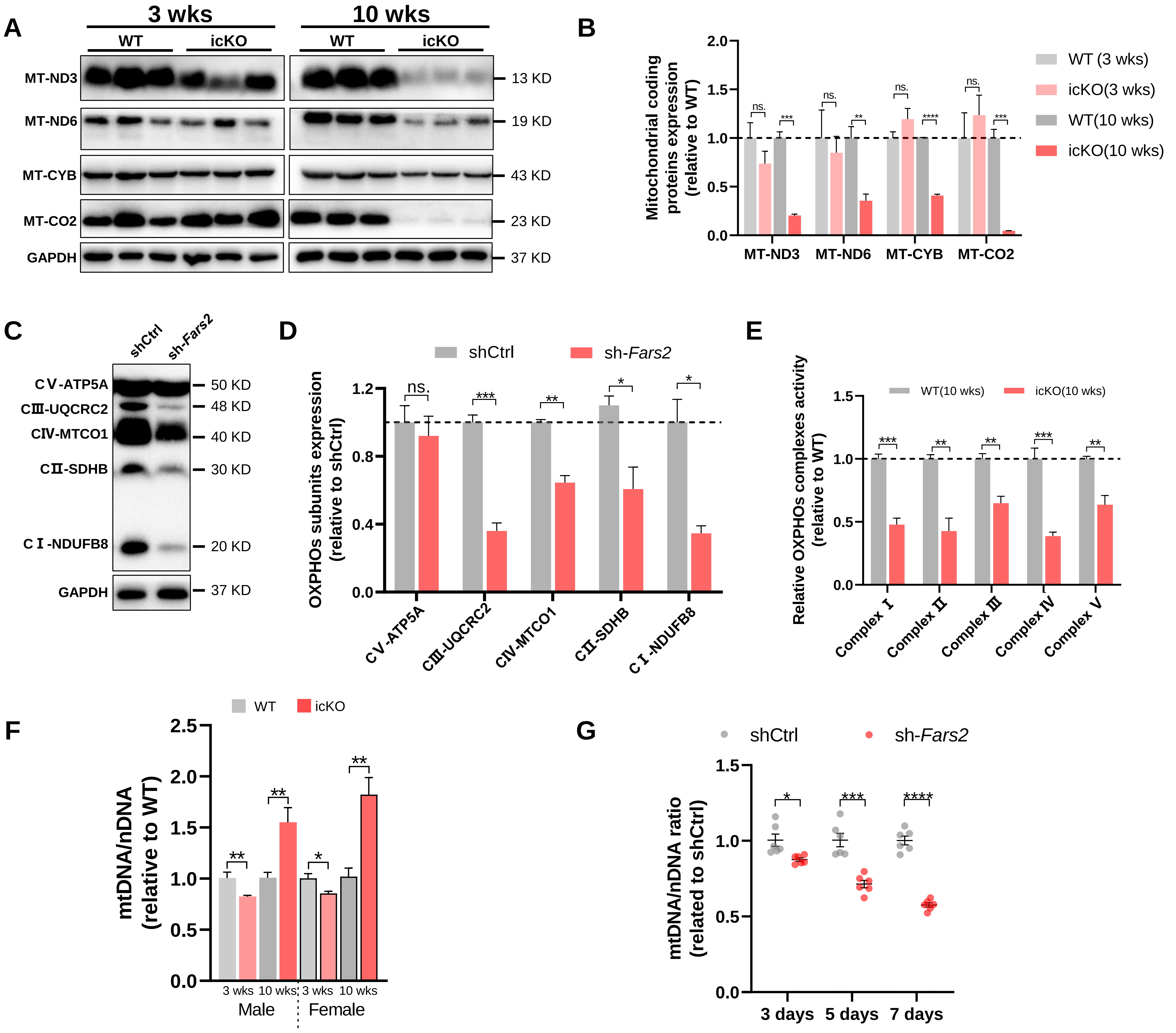

A
3 wks
10 wks
WT
icKO
MT-ND3
MT-ND6
MT-CYB
MT-CO2
GAPDH
13 KD
19 KD
43 KD
23 KD
37 KD
B
Mitochondrial coding proteins expression (relative to WT)
WT (3 wks)
icKO(3 wks)
WT(10 wks)
icKO(10 wks)
C
shCtrl
sh-Fars2
CV-ATP5A
CIII-UQCRC2
CIV-MTCO1
CII-SDHB
CI-NDUFB8
50 KD
48 KD
40 KD
30 KD
20 KD
D
OXPHOs subunits expression (relative to shCtrl)
E
Relative OXPHOs complexes activity (relative to WT)
Complex I
Complex II
Complex III
Complex IV
Complex V
F
mtDNA/nDNA (relative to WT)
Male
Female
G
mtDNA/nDNA ratio (related to shCtrl)
3 days
5 days
7 days

Figure S14

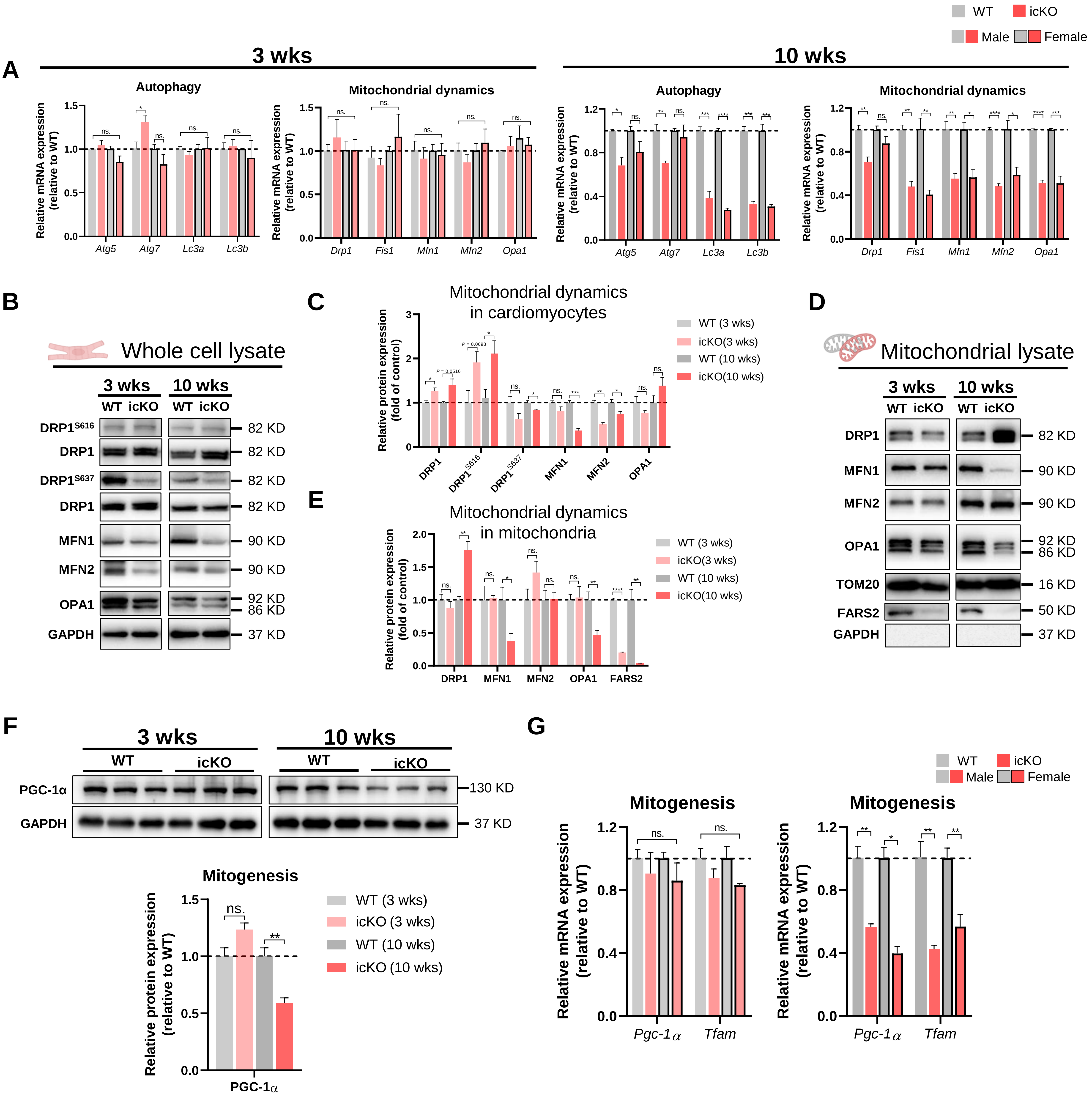

A
3 wks
10 wks
WT
icKO
Male
Female
Autophagy
Mitochondrial dynamics
Relative mRNA expression (relative to WT)
Atg5
Atg7
Lc3a
Lc3b
Drp1
Fis1
Mfn1
Mfn2
Opa1
B
Whole cell lysate
DRP1S616
DRP1
DRP1S637
MFN1
MFN2
OPA1
GAPDH
82 KD
90 KD
92 KD
86 KD
37 KD
C
Mitochondrial dynamics in cardiomyocytes
Relative protein expression (fold of control)
WT (3 wks)
icKO(3 wks)
WT (10 wks)
icKO(10 wks)
D
Mitochondrial lysate
TOM20
FARS2
16 KD
50 KD
E
Mitochondrial dynamics in mitochondria
F
PGC-1α
130 KD
Mitogenesis
Relative protein expression (relative to WT)
G
Pgc-1α
Tfam

Figure S15

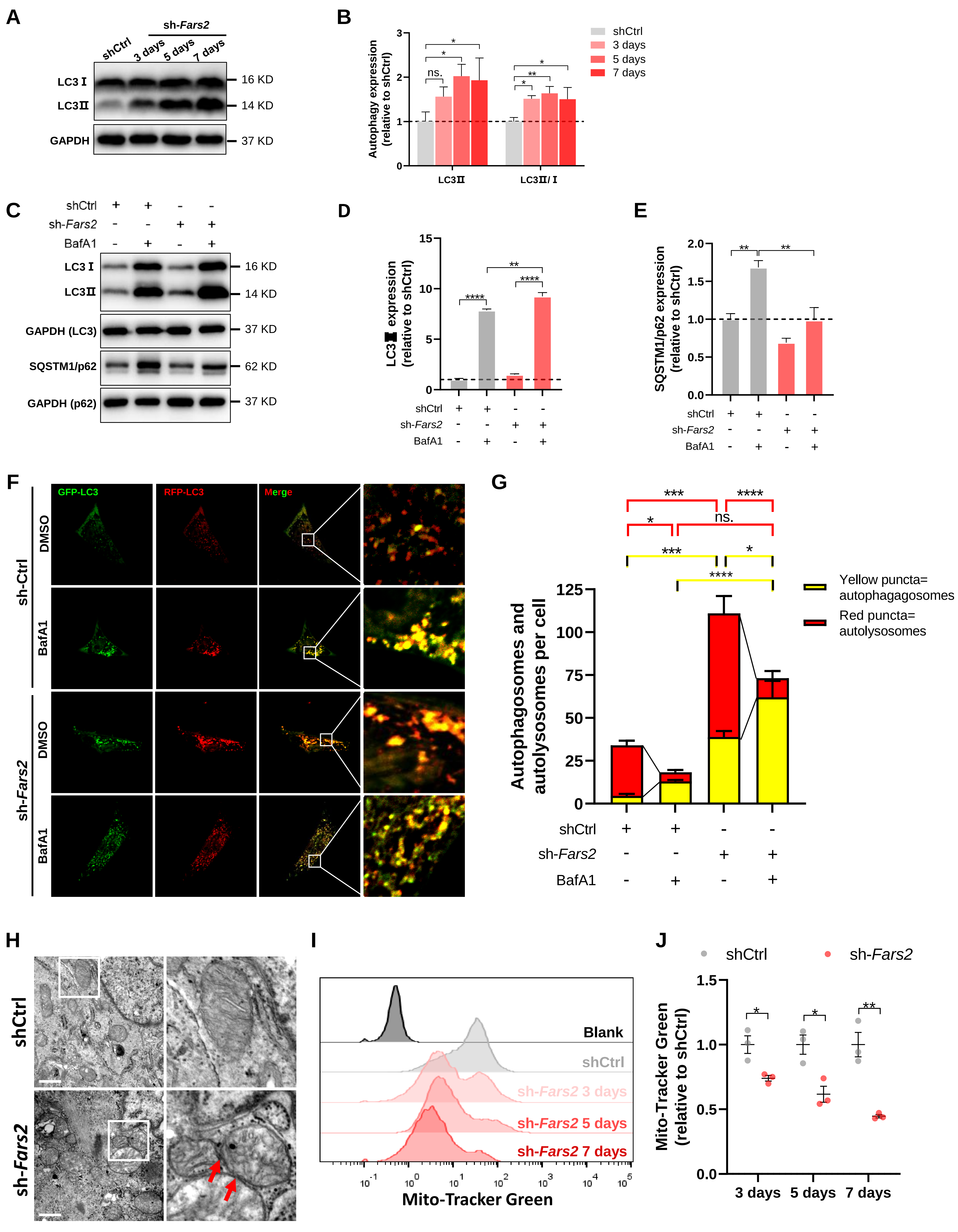

A
sh-Fars2
shCtrl
3 days
5 days
7 days
LC3 I
LC3 II
GAPDH
16 KD
14 KD
37 KD
B
Autophagy expression (relative to shCtrl)
shCtrl
3 days
5 days
7 days
LC3 II
LC3 II / I
C
shCtrl
sh-Fars2
BafA1
GAPDH (LC3)
SQSTM1/p62
GAPDH (p62)
62 KD
D
LC3 II expression (relative to shCtrl)
E
SQSTM1/p62 expression (relative to shCtrl)
F
GFP-LC3
RFP-LC3
Merge
sh-Ctrl
sh-Fars2
DMSO
BafA1
G
Autophagosomes and autolysosomes per cell
Yellow puncta= autophagosomes
Red puncta= autolysosomes
H
shCtrl
sh-Fars2
I
Blank
shCtrl
sh-Fars2 3 days
sh-Fars2 5 days
sh-Fars2 7 days
Mito-Tracker Green
J
shCtrl
sh-Fars2
Mito-Tracker Green (relative to shCtrl)
3 days
5 days
7 days

Figure S16

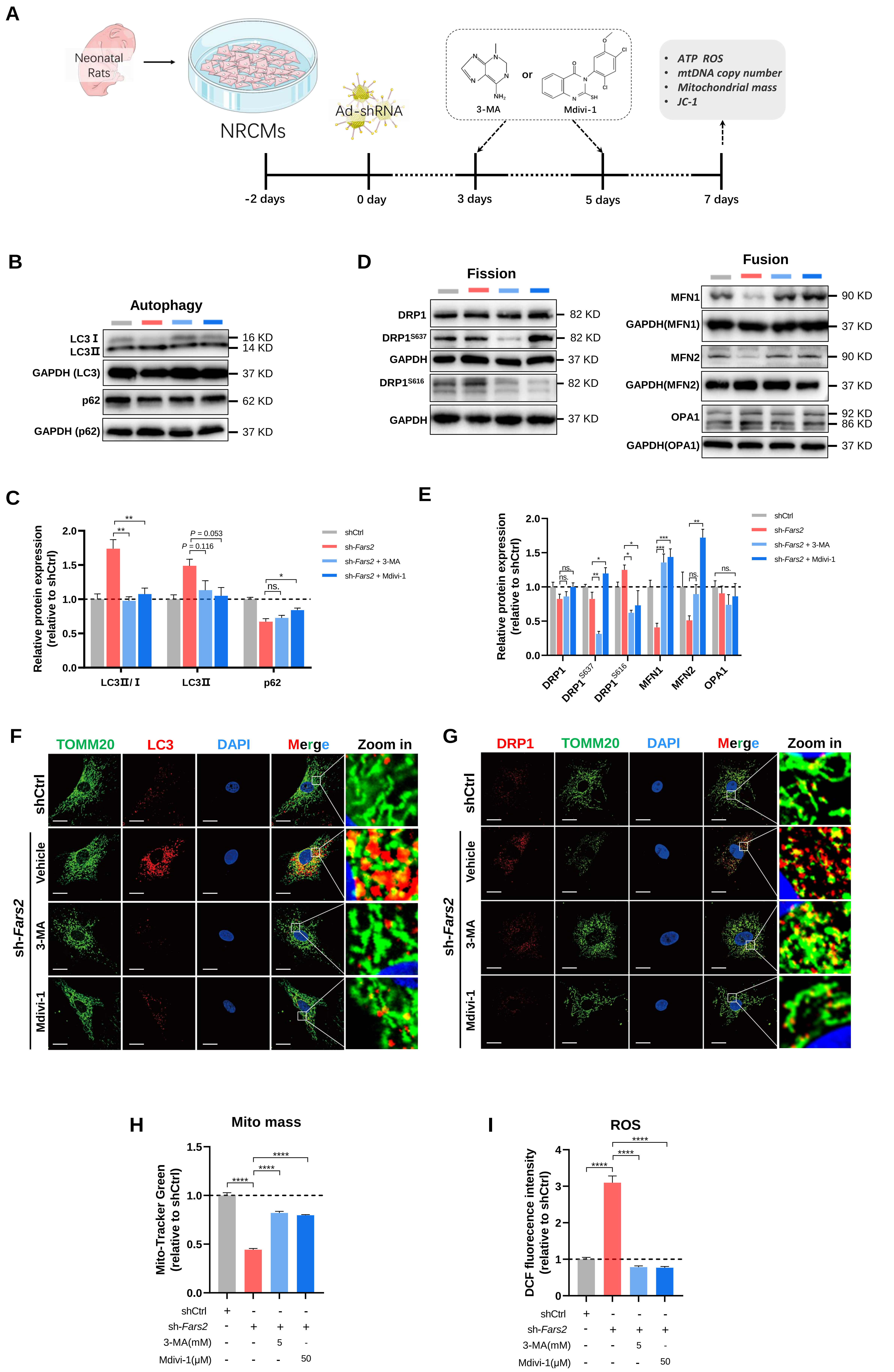

A
Neonatal Rats
NRCMs
Ad-shRNA
3-MA
or
Mdivi-1
ATP ROS
mtDNA copy number
Mitochondrial mass
JC-1
-2 days
0 day
3 days
5 days
7 days
B
Autophagy
LC3 I
LC3 II
GAPDH (LC3)
p62
GAPDH (p62)
16 KD
14 KD
37 KD
62 KD
37 KD
D
Fission
Fusion
DRP1
DRP1S637
GAPDH
DRP1S616
GAPDH
82 KD
82 KD
37 KD
82 KD
37 KD
MFN1
GAPDH(MFN1)
MFN2
GAPDH(MFN2)
OPA1
GAPDH(OPA1)
90 KD
37 KD
90 KD
37 KD
92 KD
86 KD
37 KD
C
Relative protein expression (relative to shCtrl)
shCtrl
sh-Fars2
sh-Fars2 + 3-MA
sh-Fars2 + Mdivi-1
P = 0.053
P = 0.116
ns.
LC3II/I
LC3II
p62
E
Relative protein expression (relative to shCtrl)
DRP1
DRP1 S637
DRP1 S616
MFN1
MFN2
OPA1
F
TOMM20
LC3
DAPI
Merge
Zoom in
shCtrl
Vehicle
3-MA
Mdivi-1
sh-Fars2
G
DRP1
TOMM20
DAPI
Merge
Zoom in
H
Mito mass
Mito-Tracker Green (relative to shCtrl)
shCtrl
sh-Fars2
3-MA(mM)
Mdivi-1(μM)
I
ROS
DCF fluorecence intensity (relative to shCtrl)

Figure S17

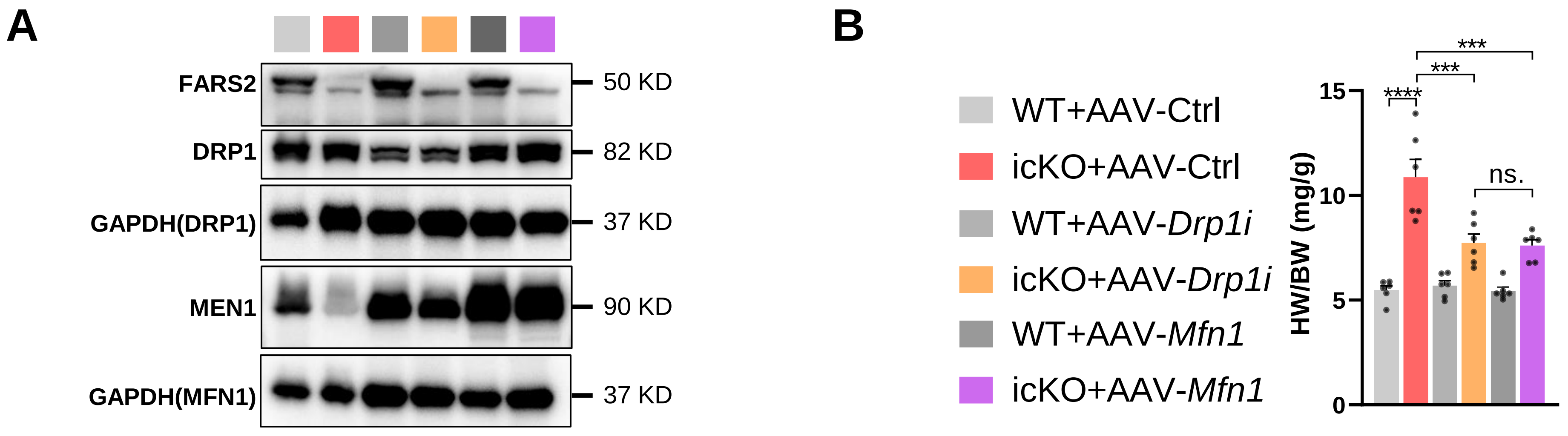
A
FARS2
DRP1
GAPDH(DRP1)
MEN1
GAPDH(MFN1)
50 KD
82 KD
37 KD
90 KD
37 KD
B
WT+AAV-Ctrl
icKO+AAV-Ctrl
WT+AAV-*Drp1i*
icKO+AAV-*Drp1i*
WT+AAV-*Mfn1*
icKO+AAV-*Mfn1*
HW/BW (mg/g)
15
10
5
0
****
***
***
ns.
