# Supplementary material for: FARS2 Deficiency Causes Cardiomyopathy by Disrupting Mitochondrial Homeostasis and the Mitochondrial Quality Control System
Source: Circulation. 2024 Feb 16;149(16):1268–84. doi: 10.1161/CIRCULATIONAHA.123.064489 (PMC11017836; doi:10.1161/CIRCULATIONAHA.123.064489)

Unedited-gel

FARS2 Deficiency Causes Cardiomyopathy by Disrupting Mitochondrial Homeostasis and the Mitochondrial Quality Control System

Running Title: FARS2 deficiency causes cardiomyopathy

- The representative blots shown in the manuscript are indicated by red rectangles.
- The blots used for quantification are indicated by yellow rectangles.

Full unedited gel for Figure 1E

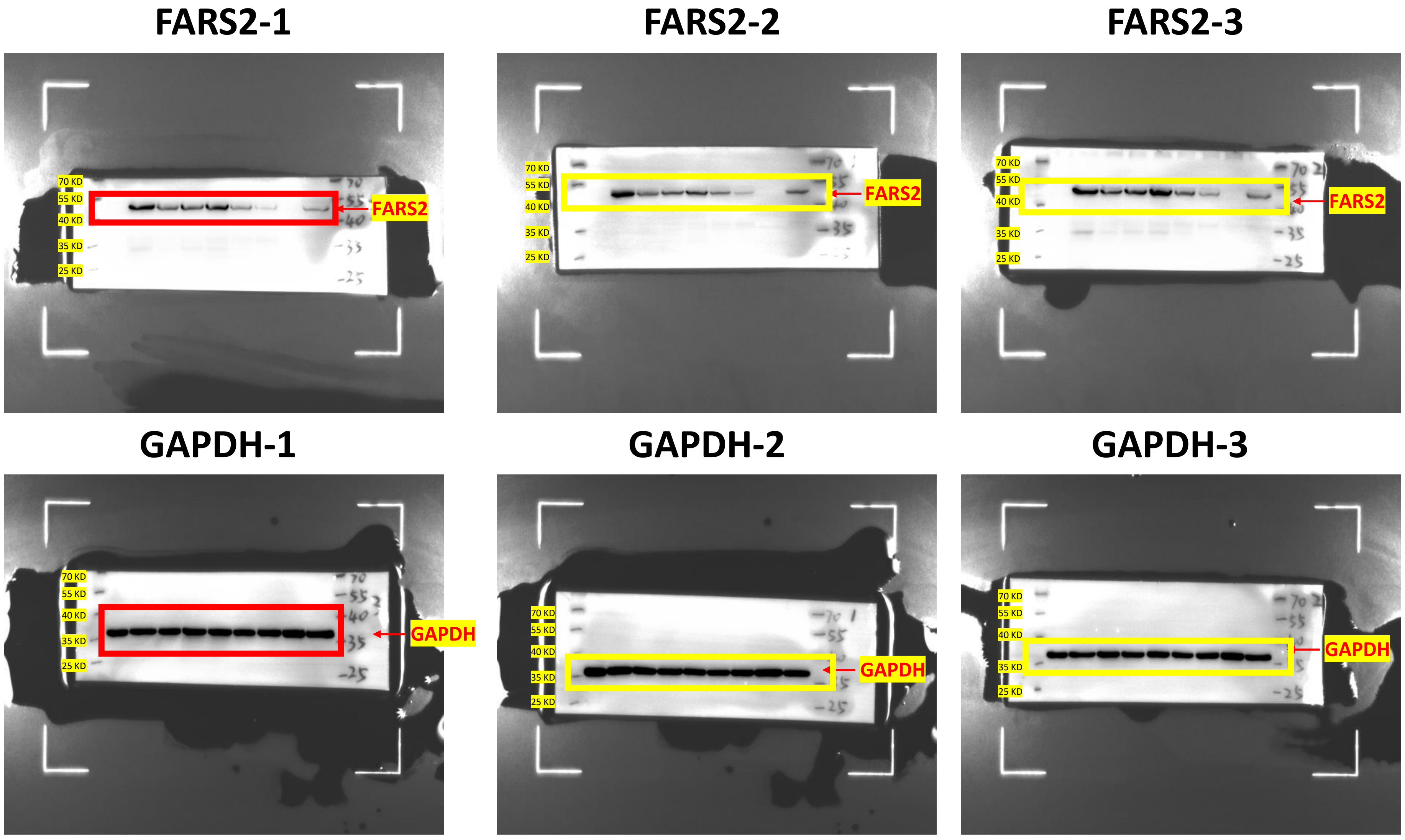

Full unedited gel for Figure 2B

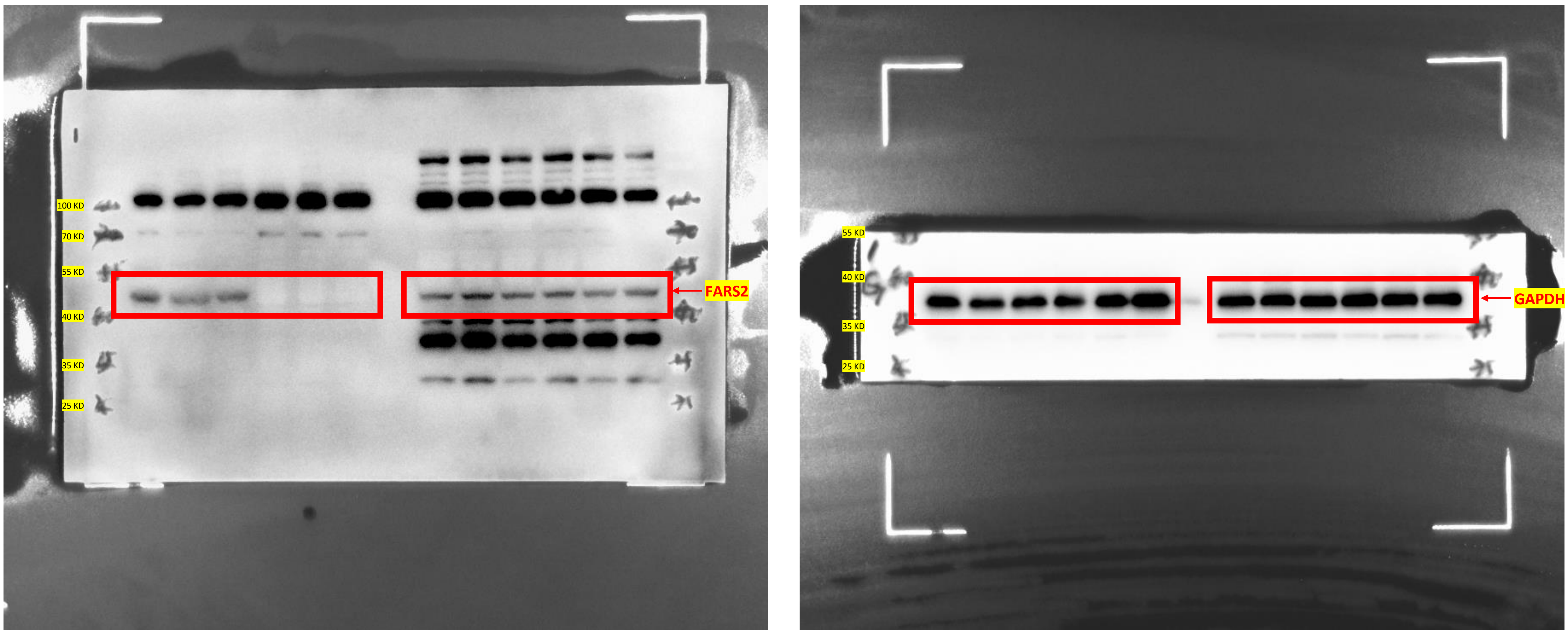

Full unedited gel for Figure 4C

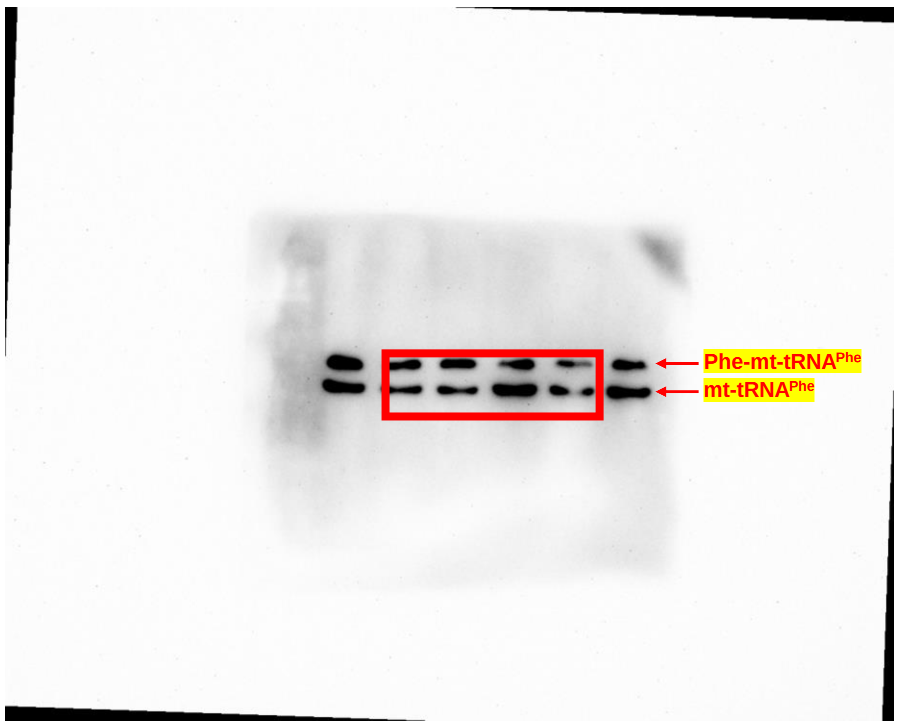

Full unedited gel for Figure 4D

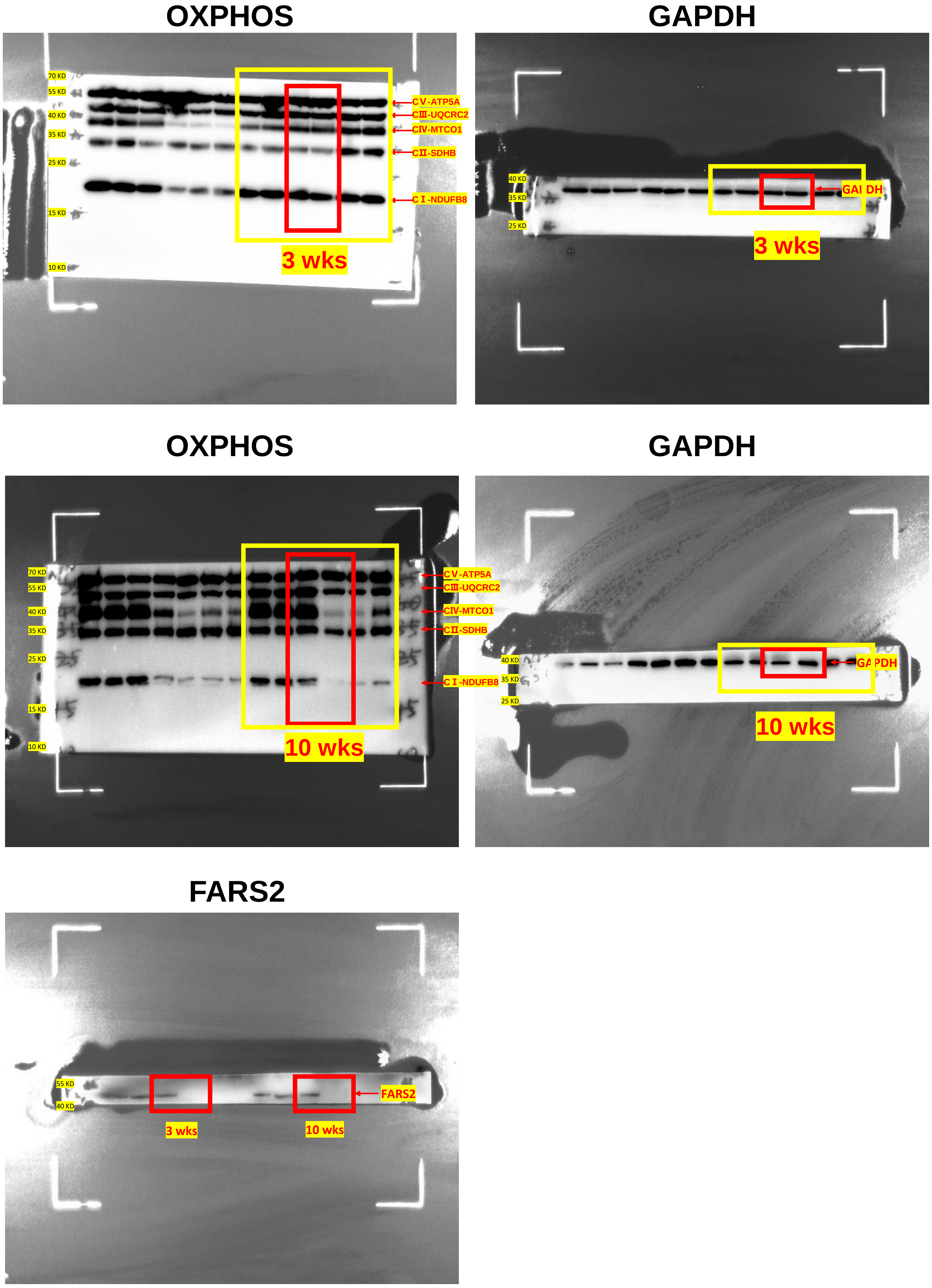

Full unedited gel for Figure 5C

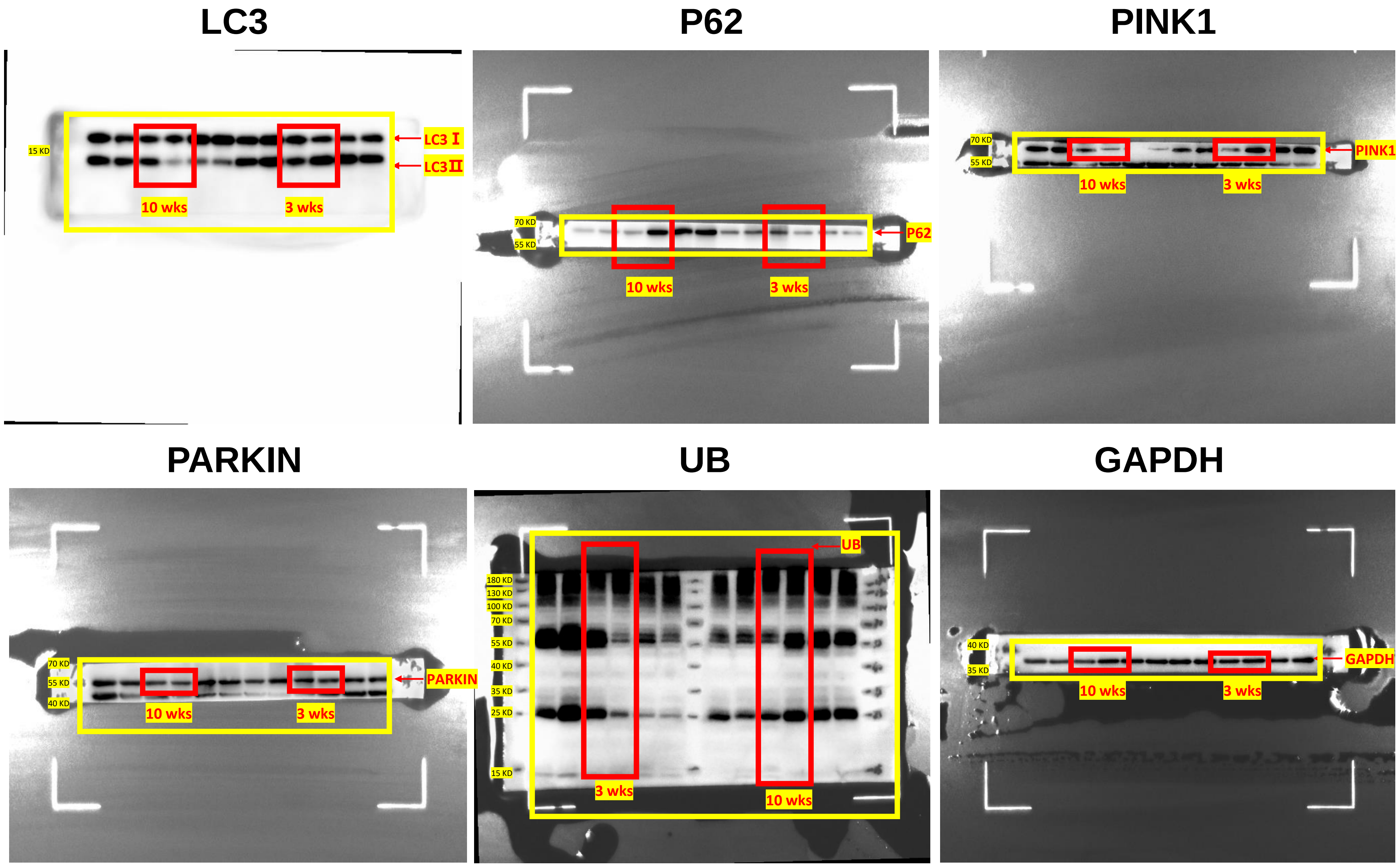

Full unedited gel for Figure 5E

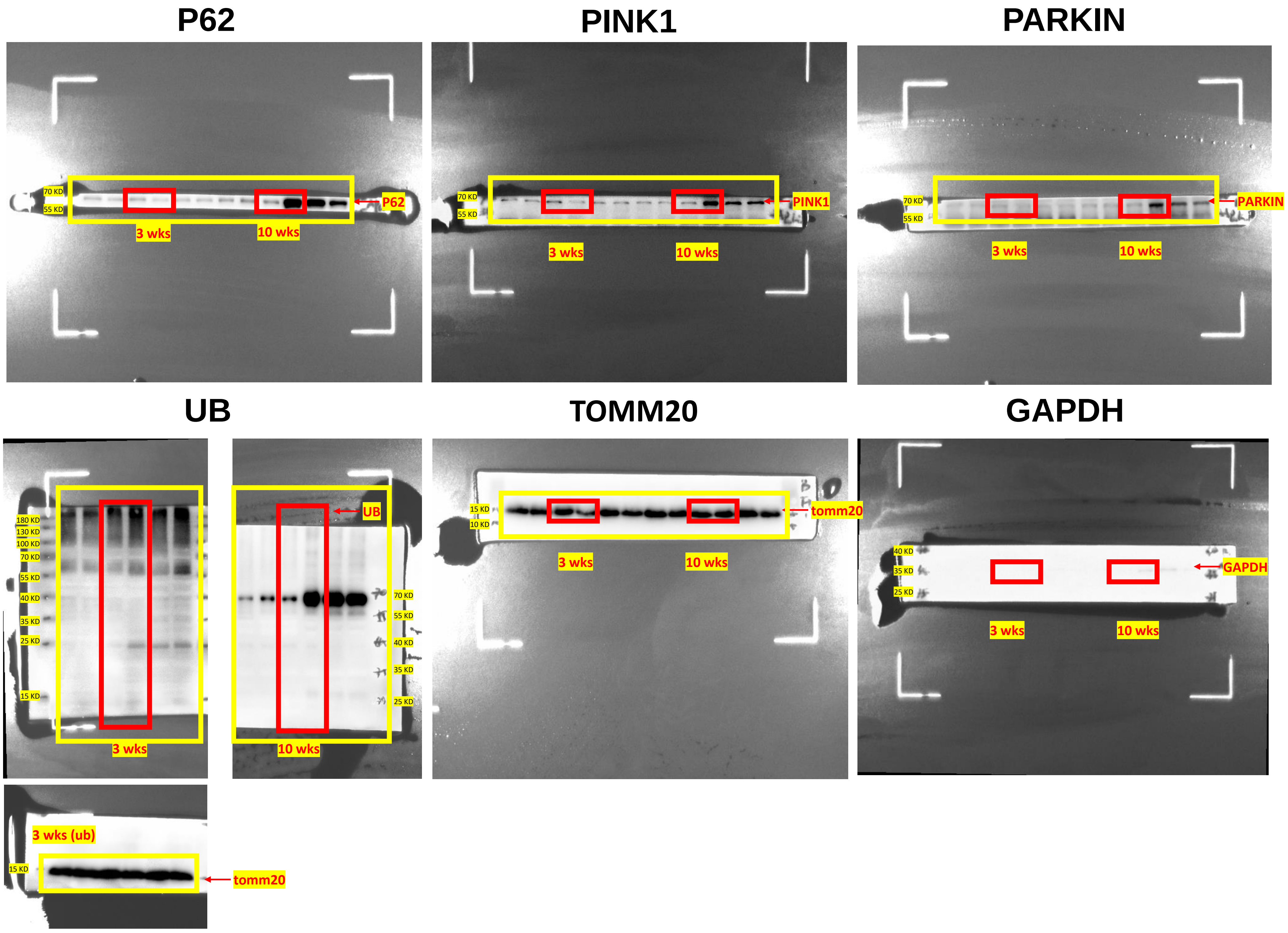

Full unedited gel for Figure 6A

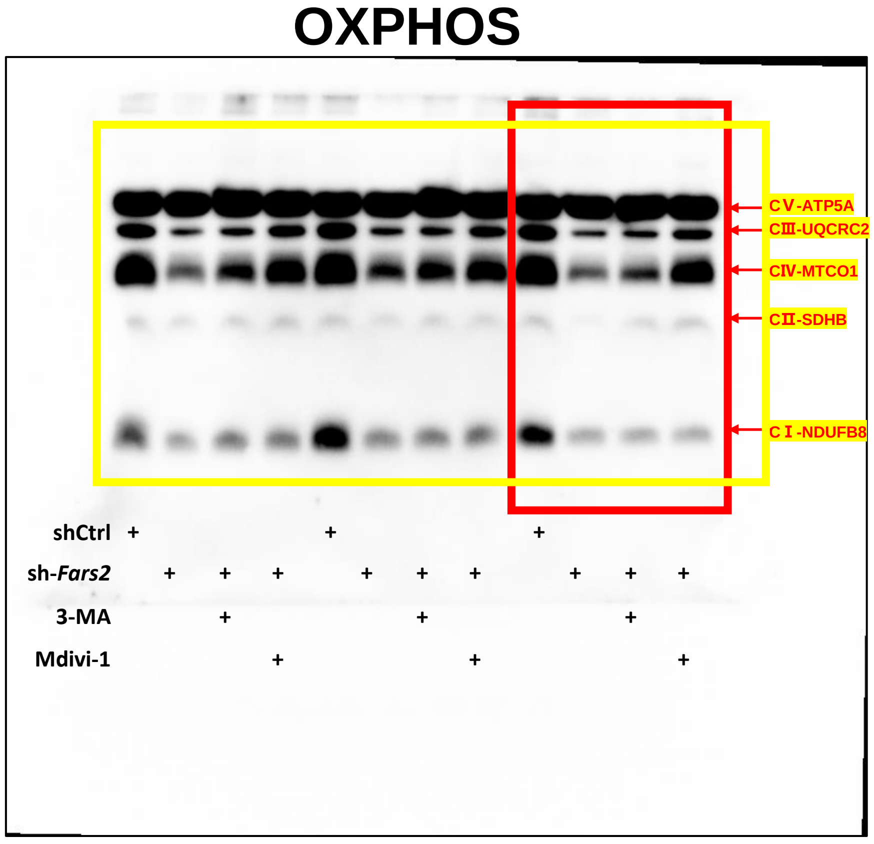

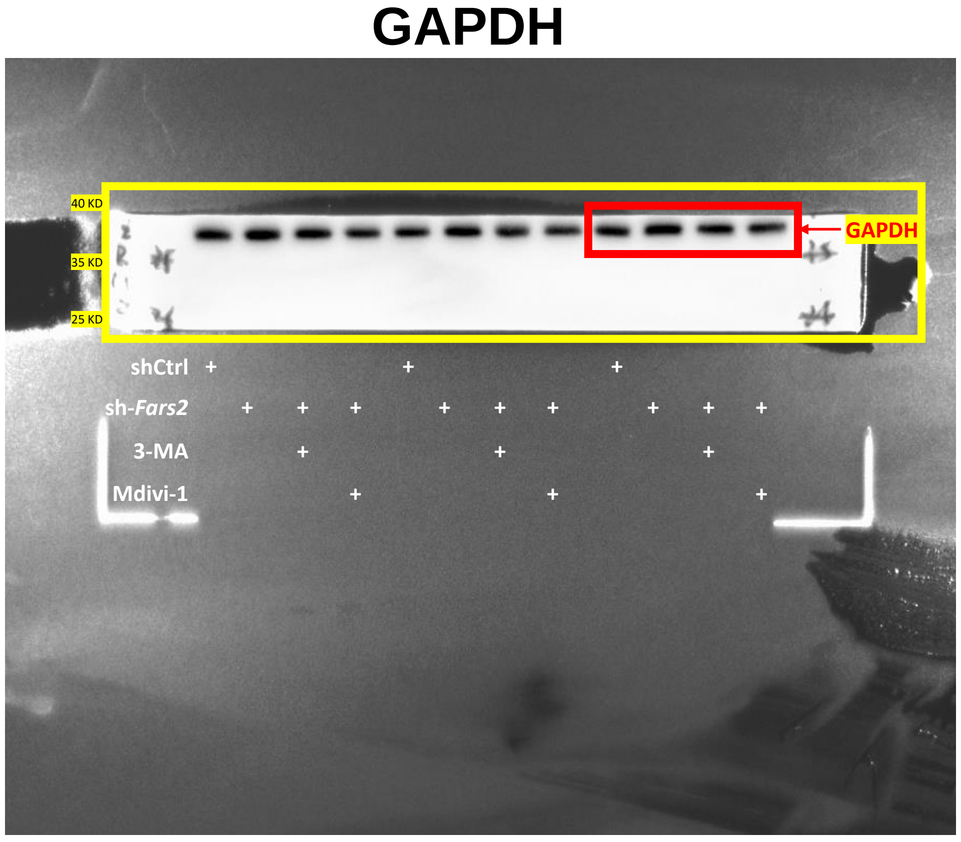

Full unedited gel for Figure S3A

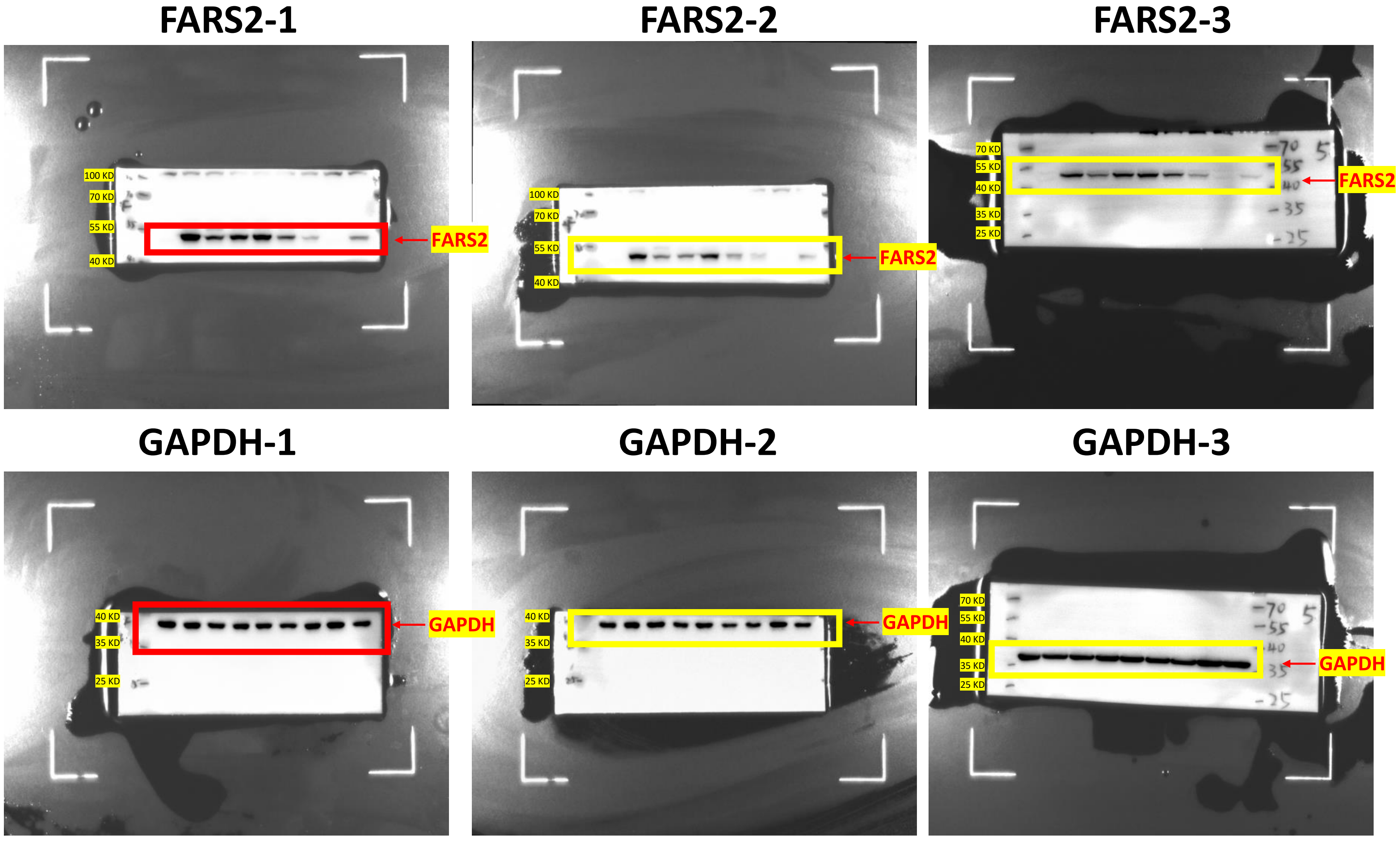

Full unedited gel for Figure S3E

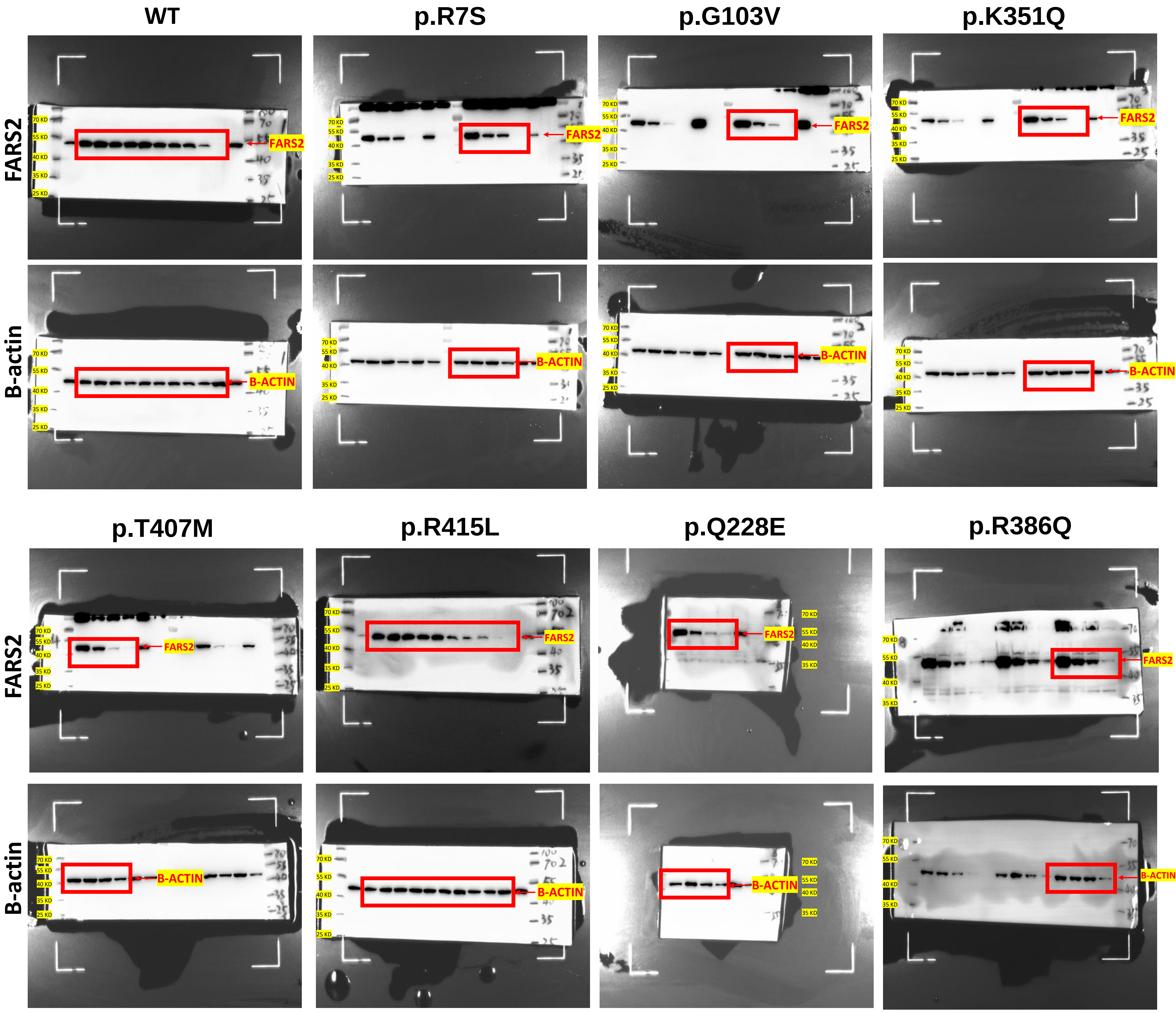

Full unedited gel for Figure S4B

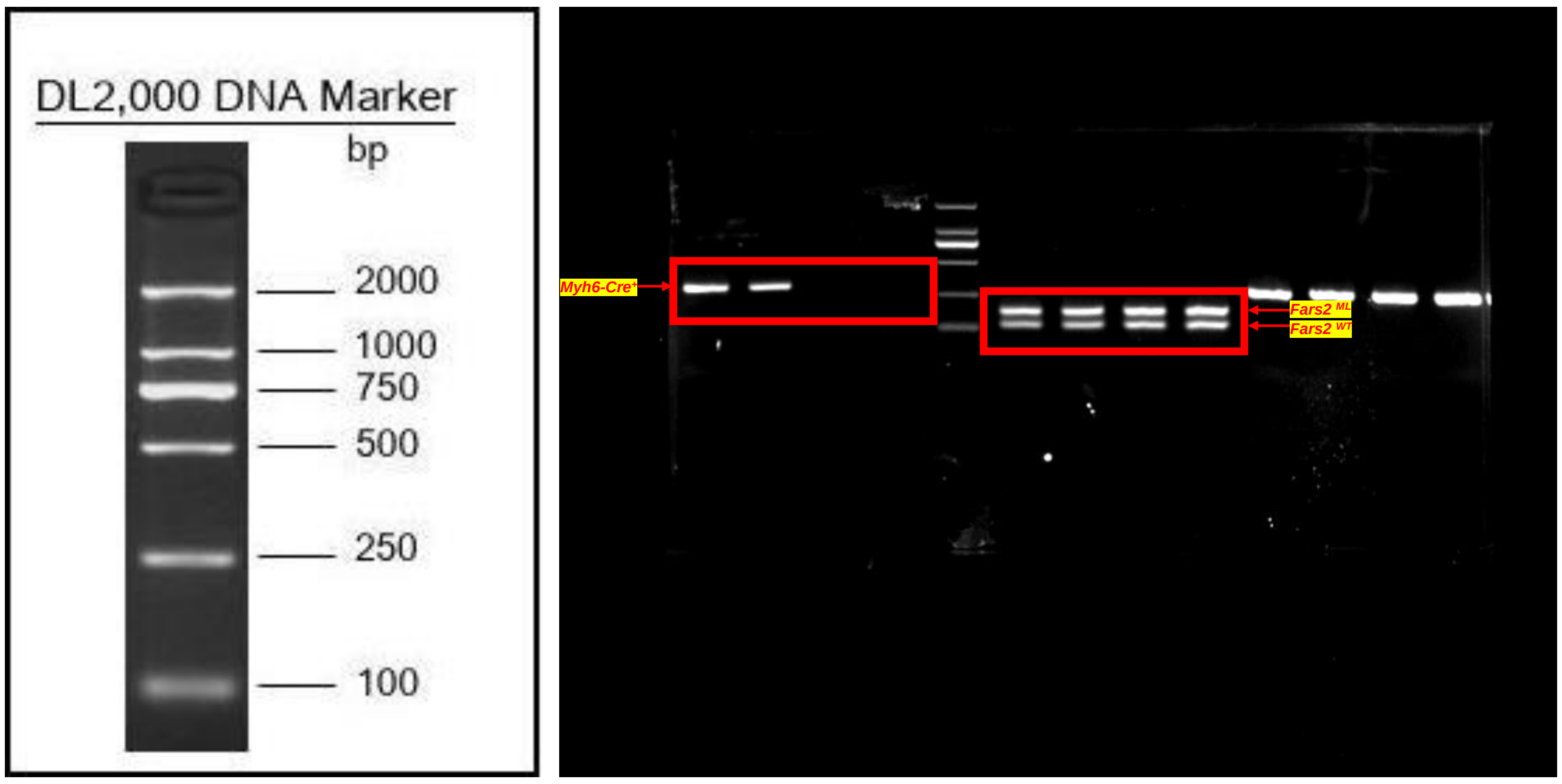

Full unedited gel for Figure S4E

FARS2

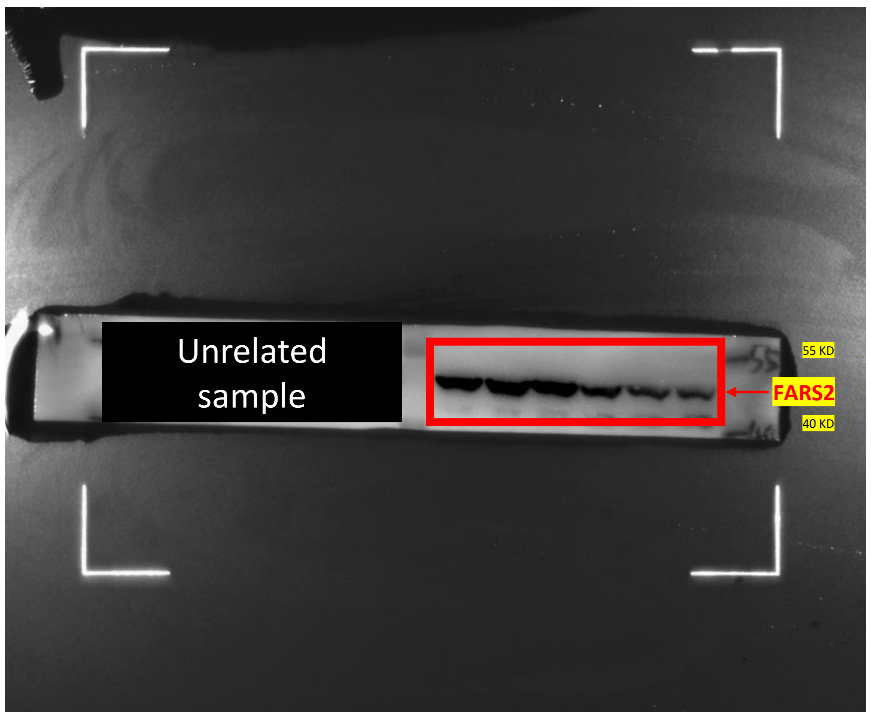

GAPDH

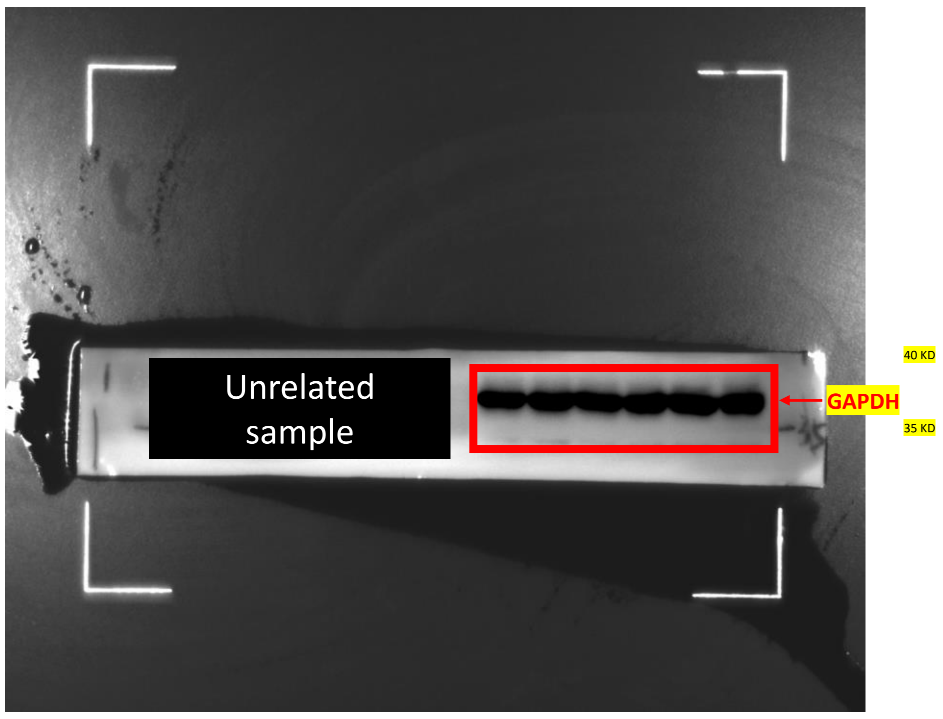

HA

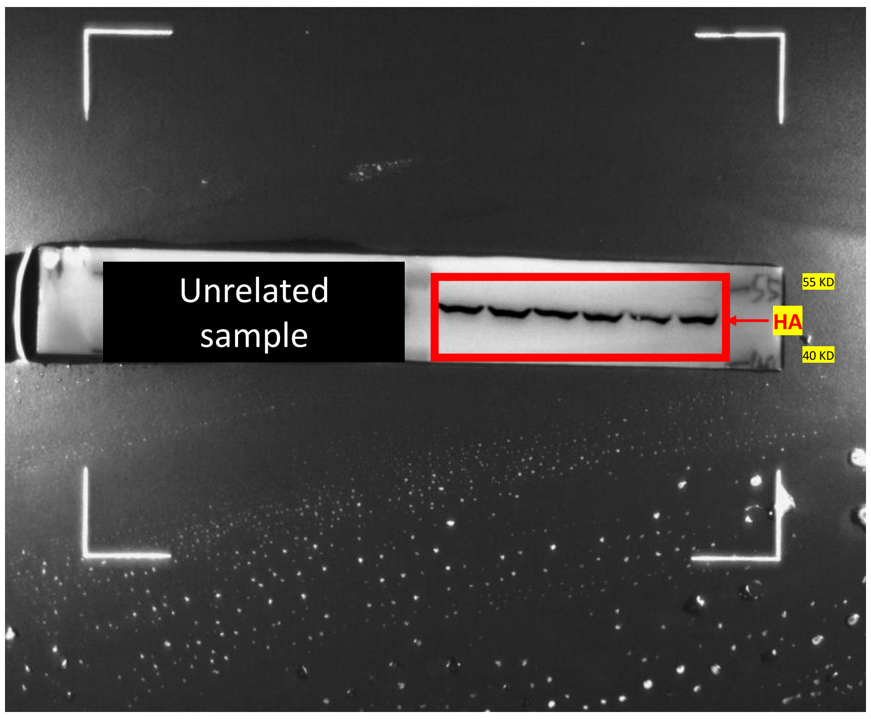

HIS

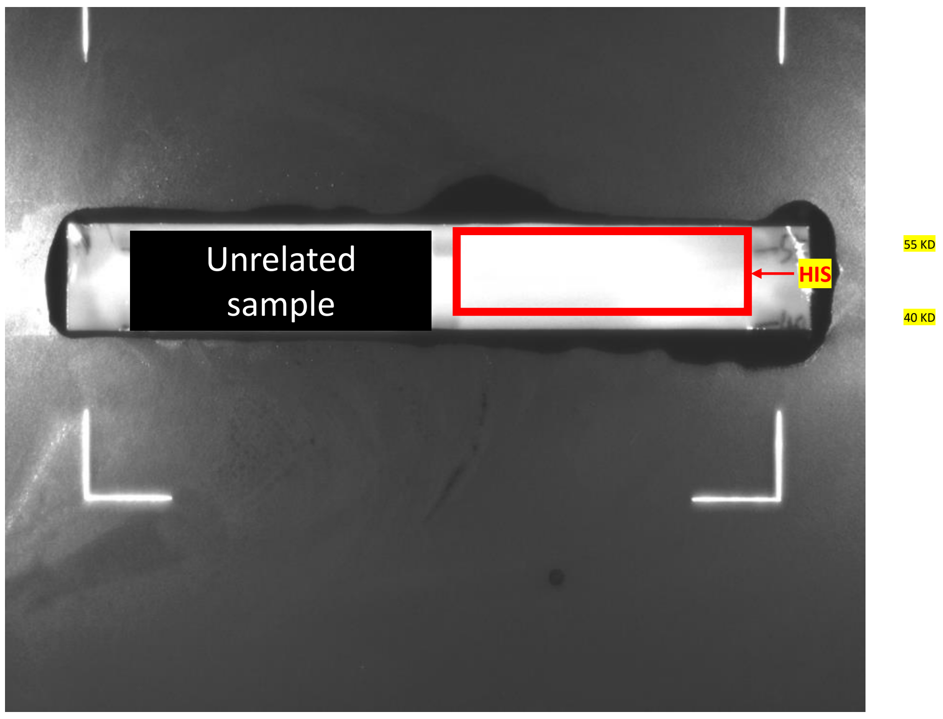

Full unedited gel for Figure S7B

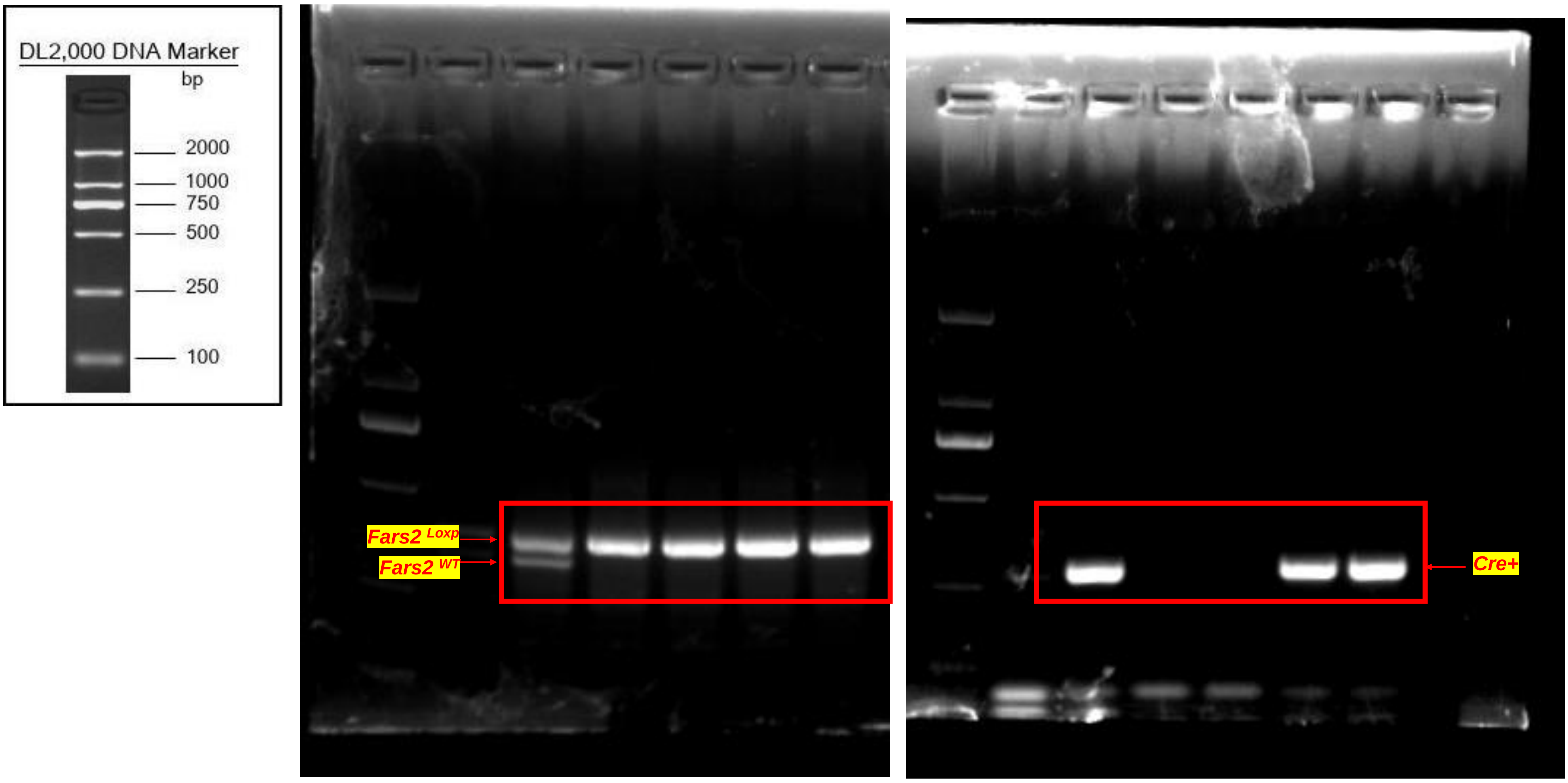

Full unedited gel for Figure S7G

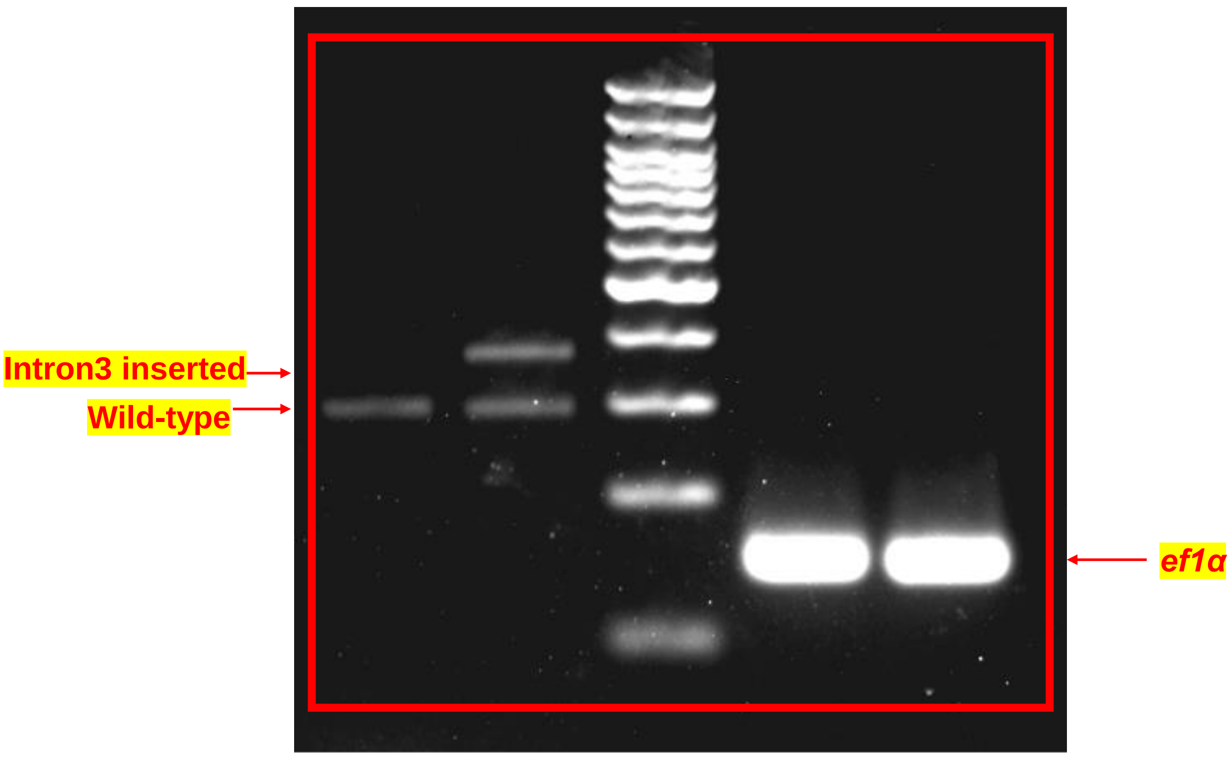

Full unedited gel for Figure S10C

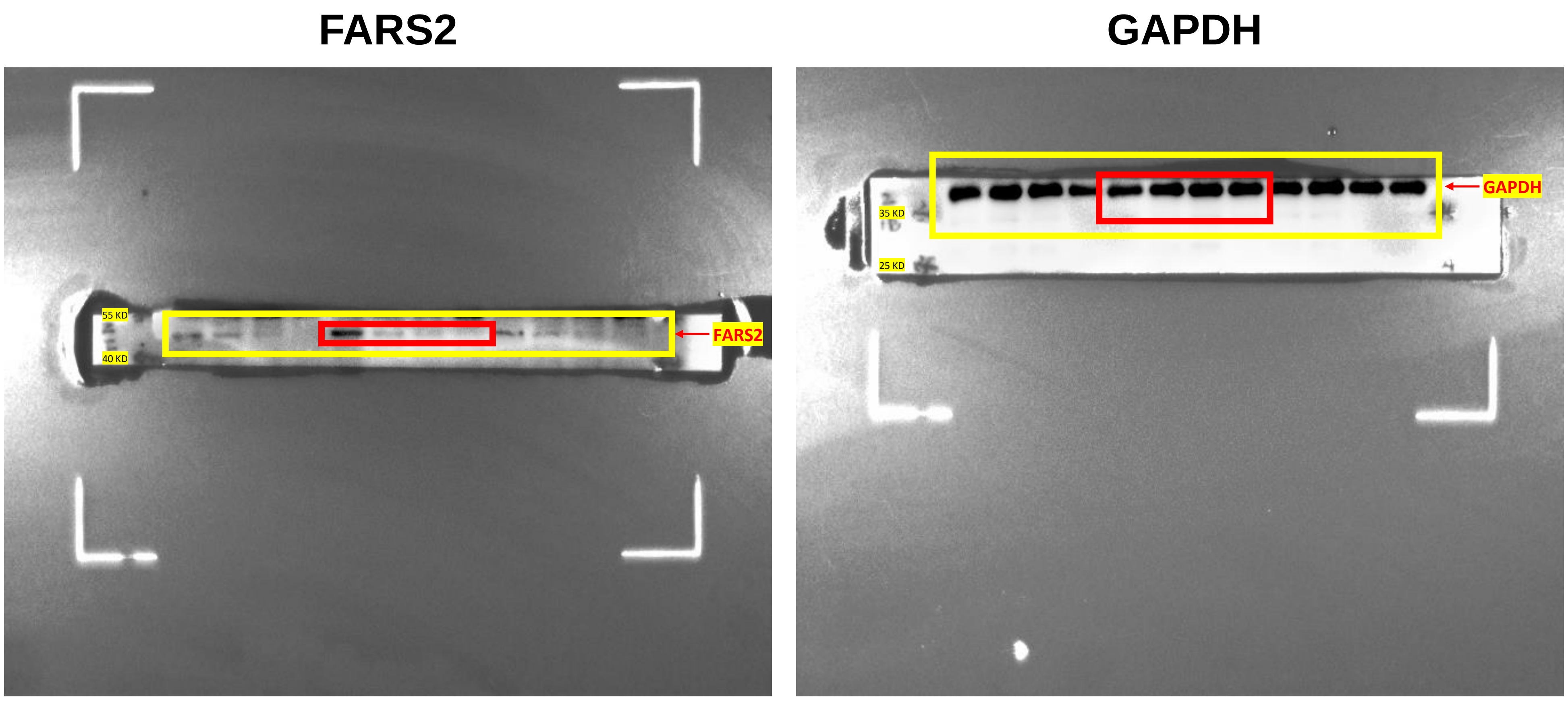

Full unedited gel for Figure S13A

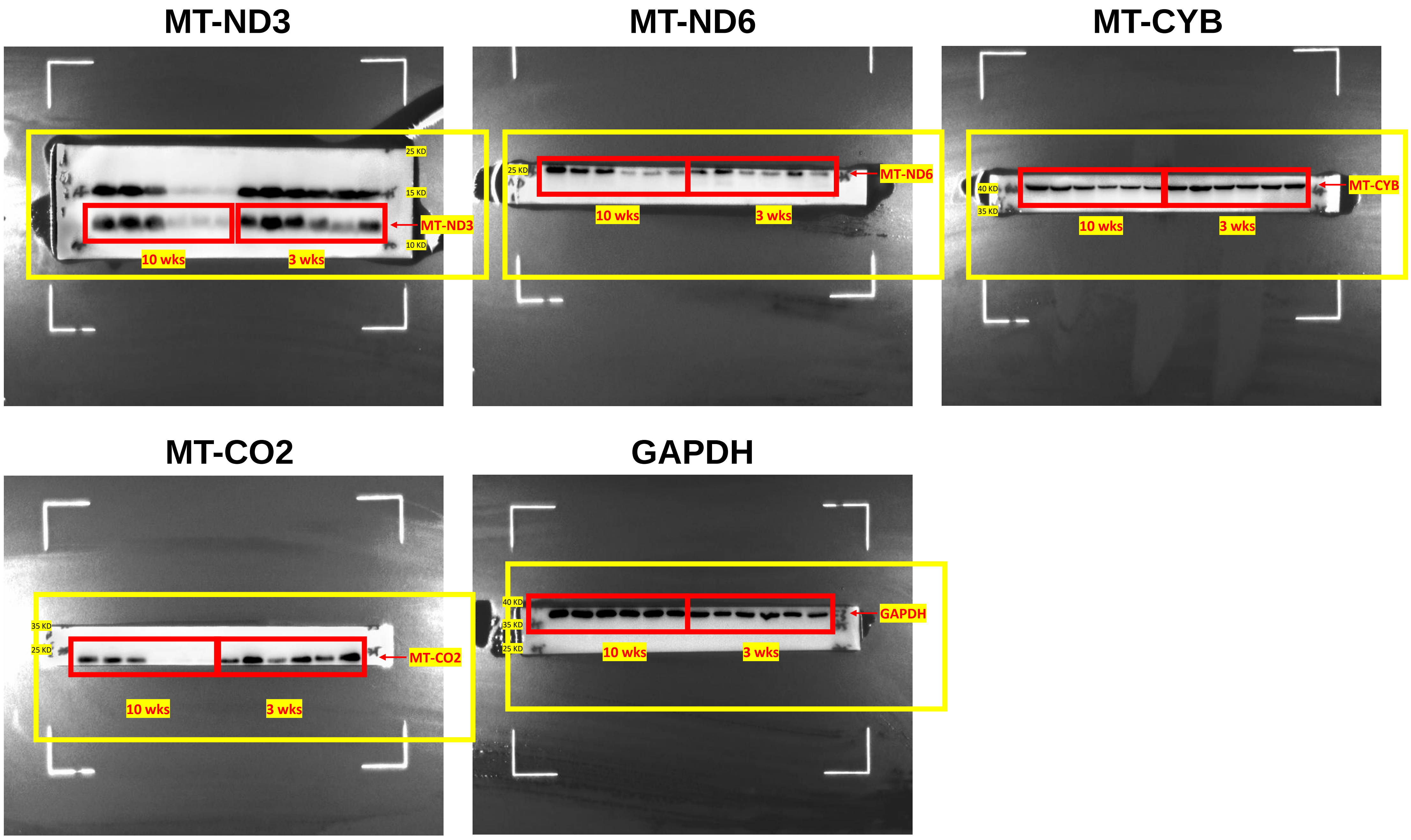

Full unedited gel for Figure S13C

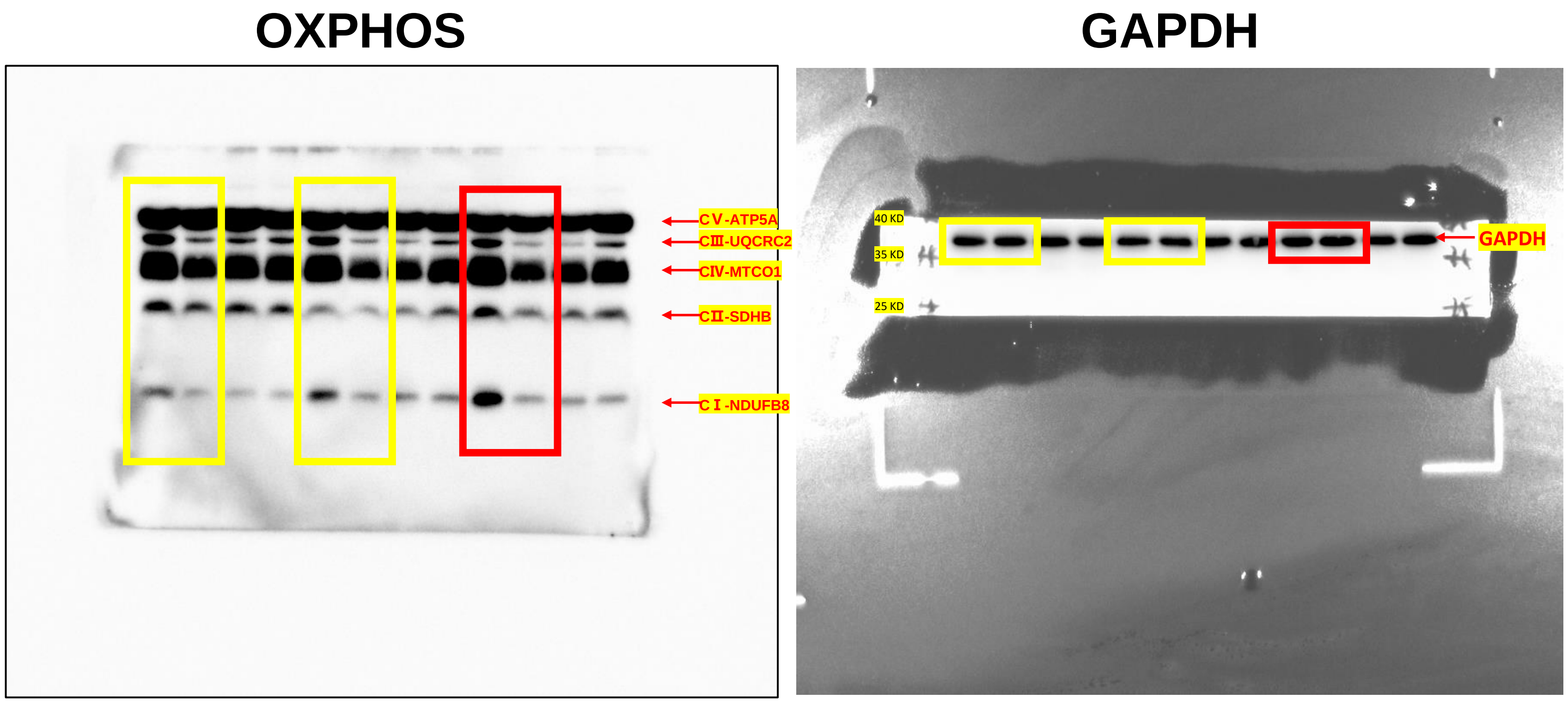

Full unedited gel for Figure S14B

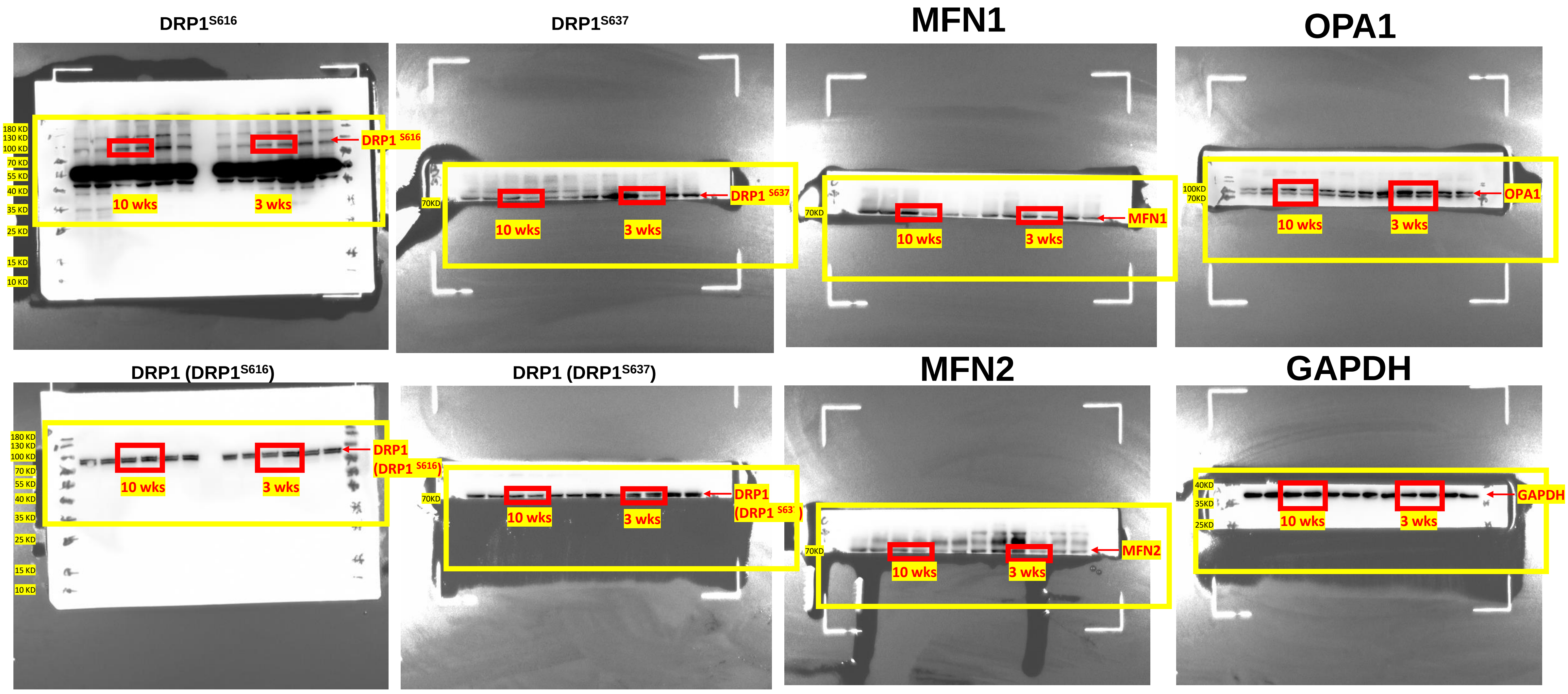

Full unedited gel for Figure S14D

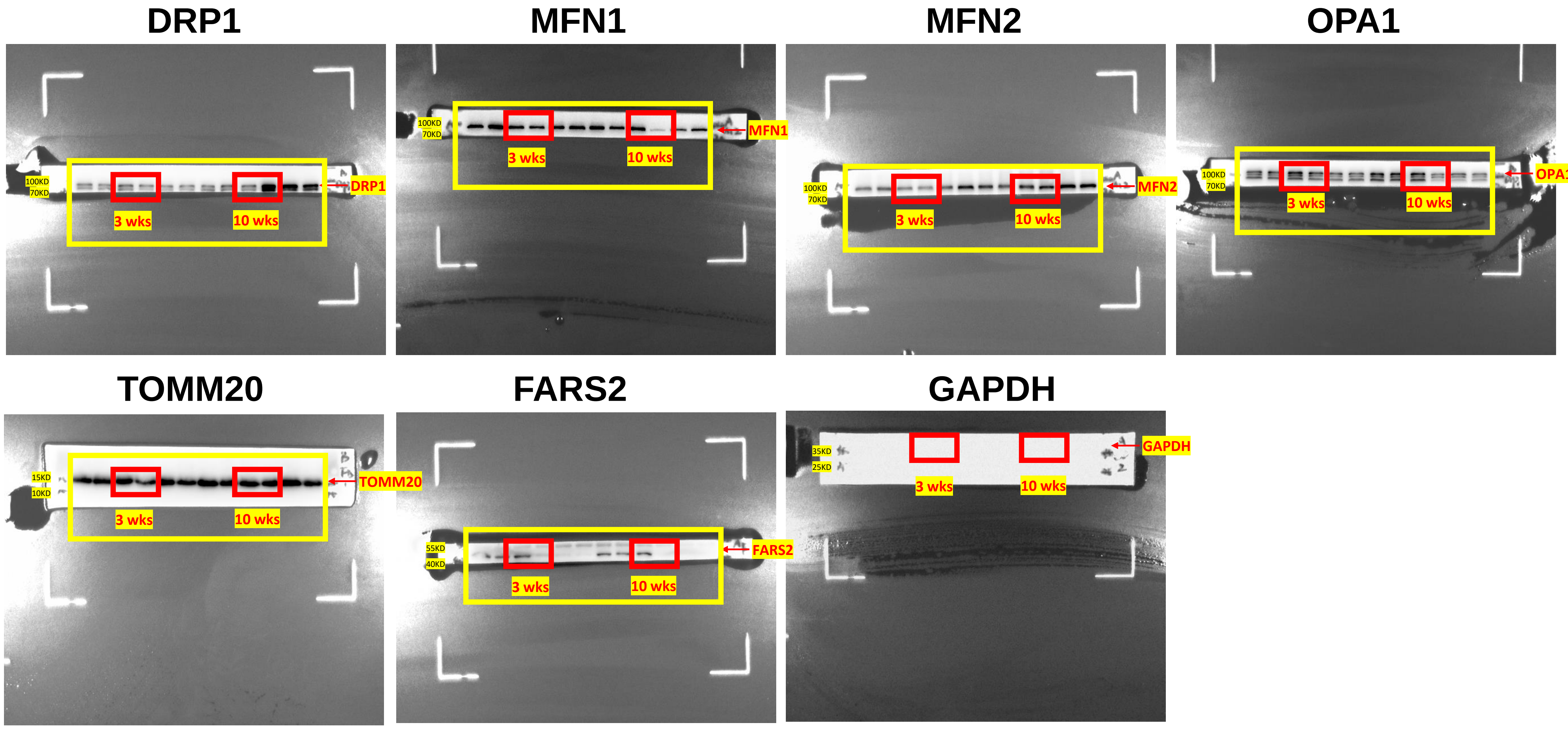

Full unedited gel for Figure S14F

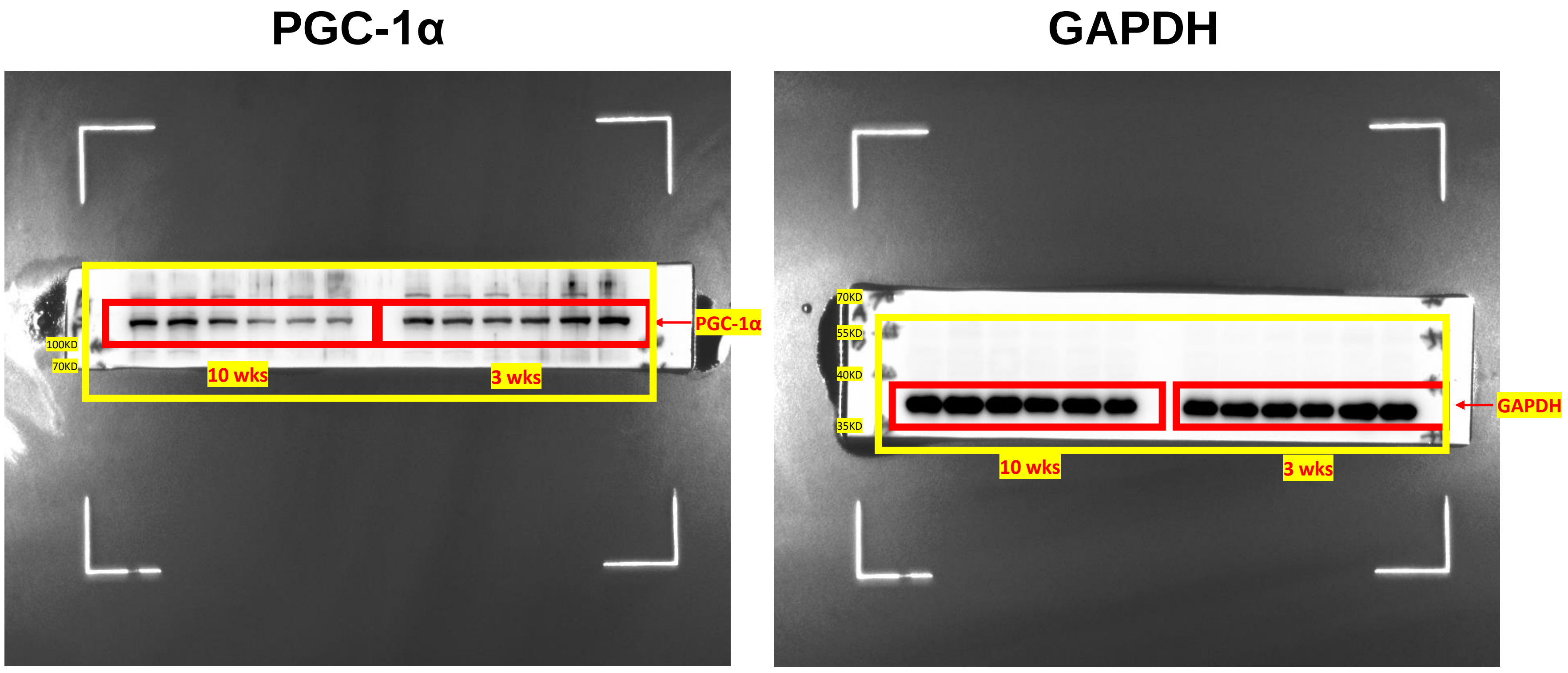

Full unedited gel for Figure S15A

LC3

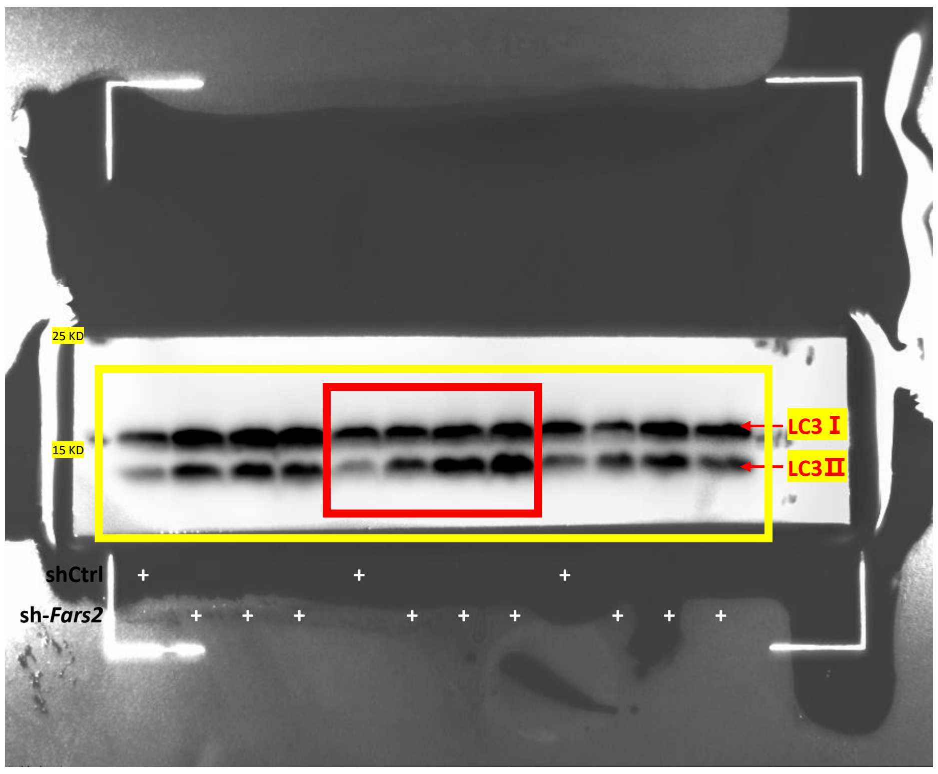

GAPDH

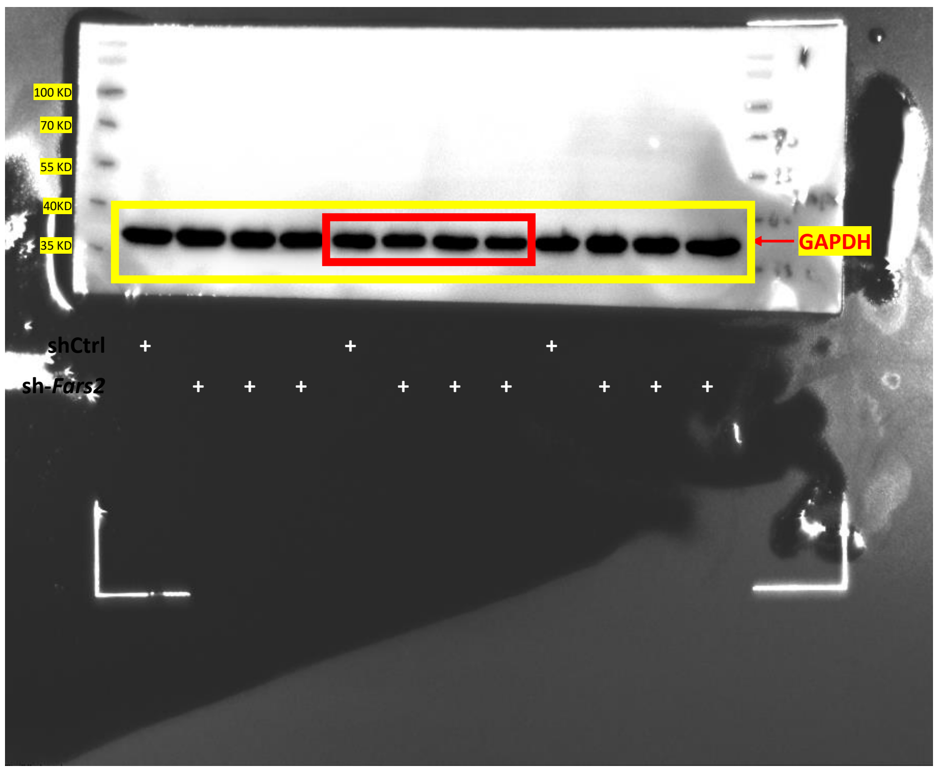

Full unedited gel for Figure S15C

LC3

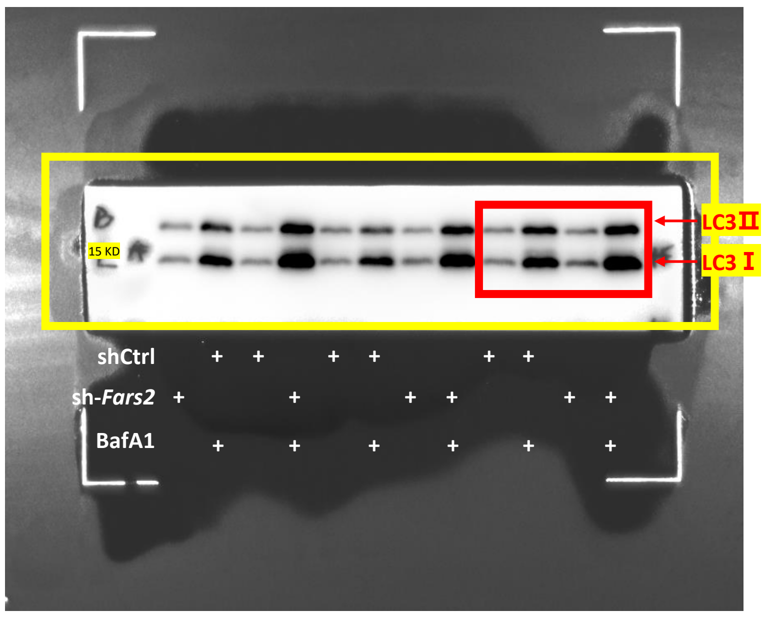

P62

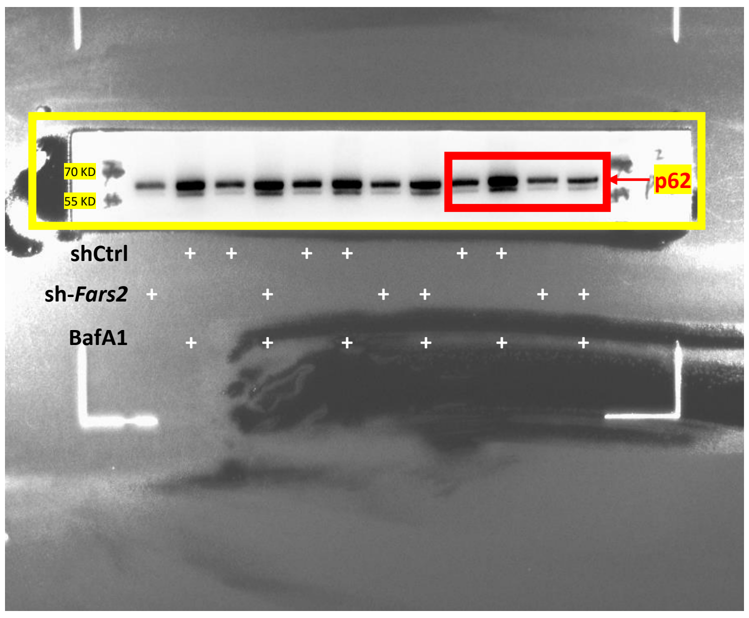

GAPDH (LC3)

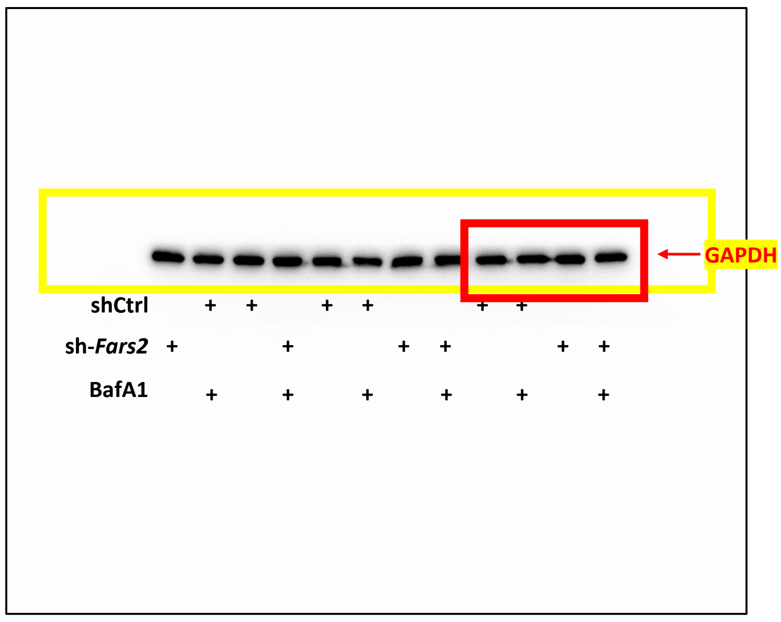

GAPDH (P62)

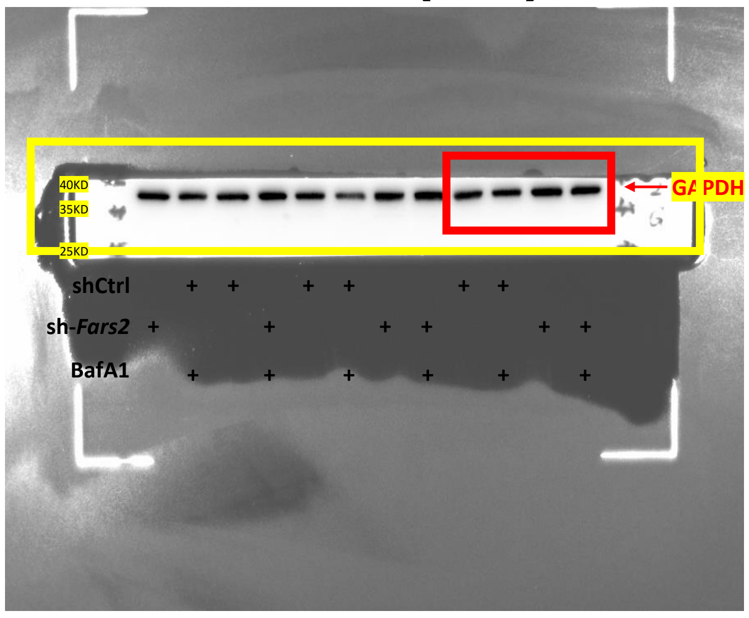

Full unedited gel for Figure S16B

LC3

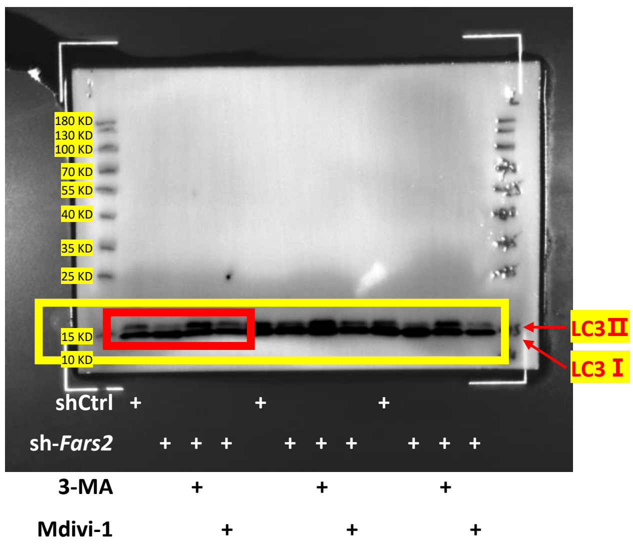

GAPDH (LC3)

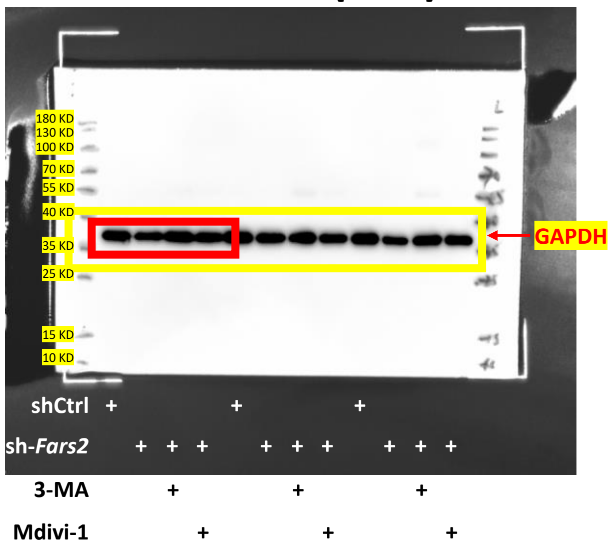

P62

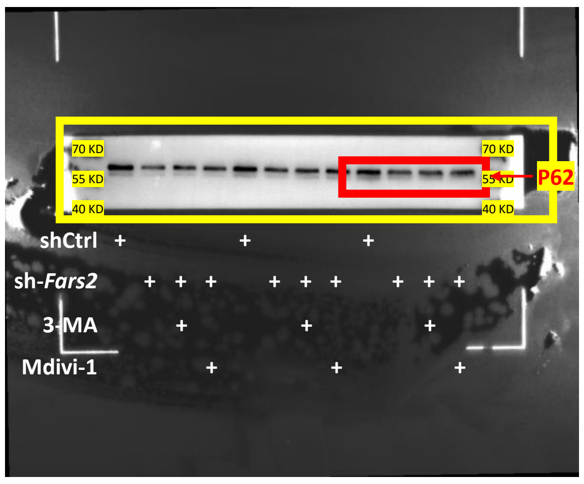

GAPDH (P62)

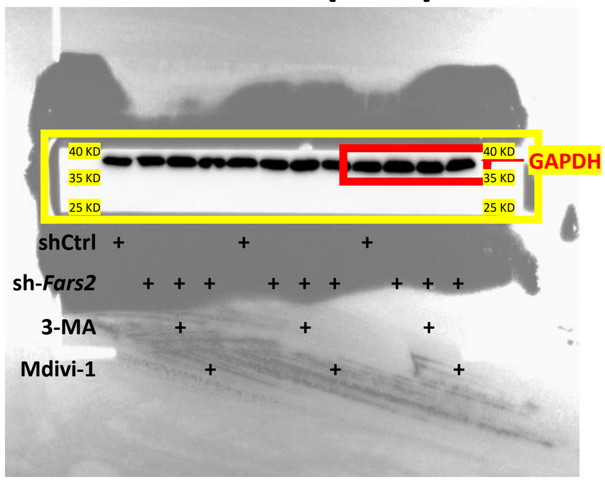

Full unedited gel for Figure S16D

DRP1 S637

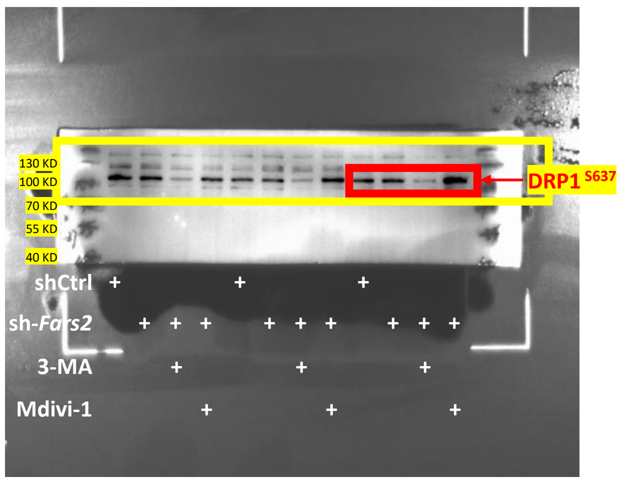

DRP1 S616

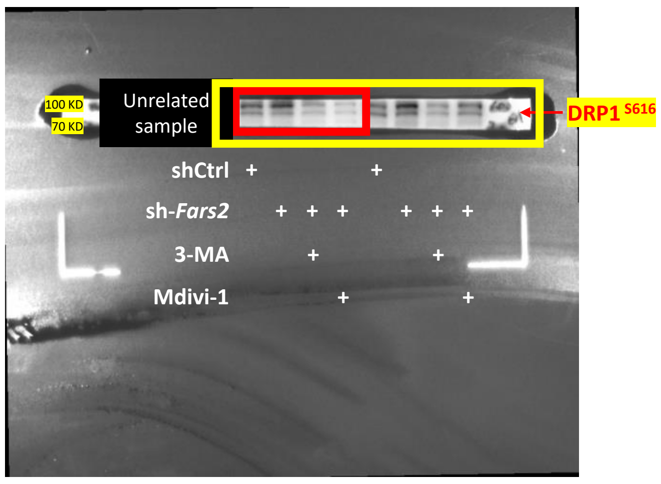

DRP1 S616

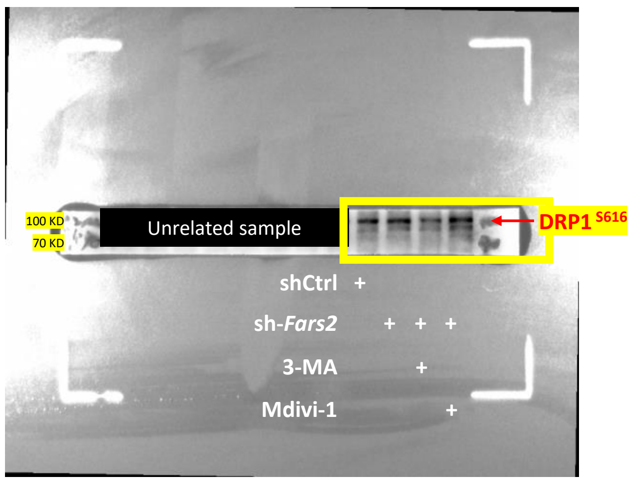

DRP1

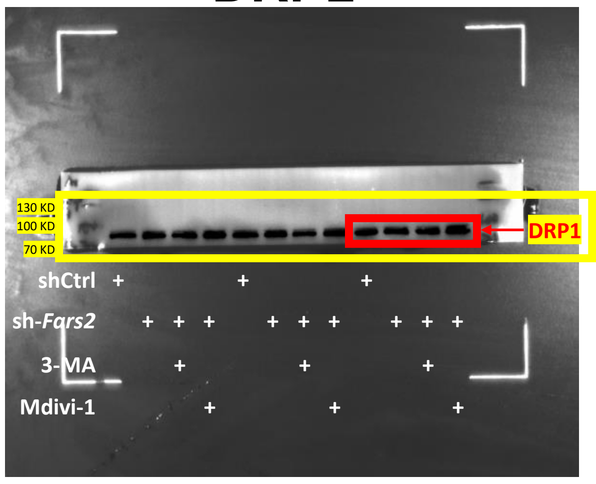

GAPDH (DRP1 S616)

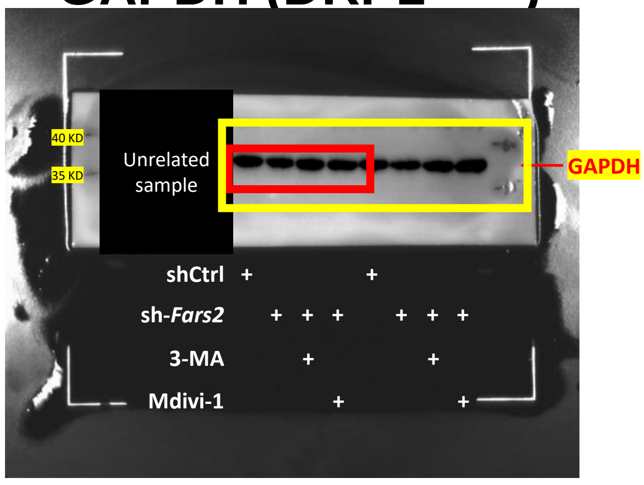

GAPDH (DRP1 S616)

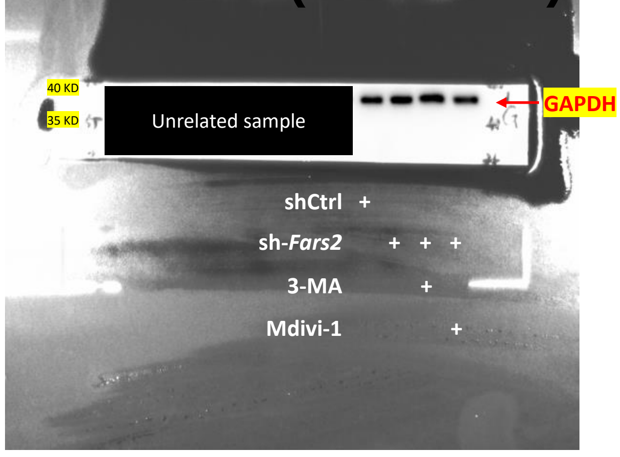

OPA1

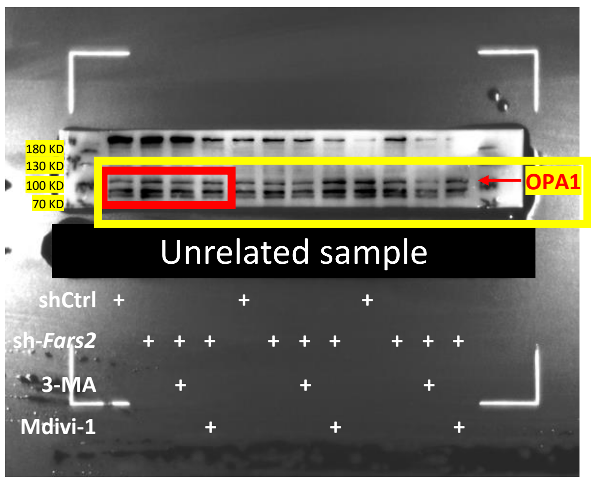

MFN1

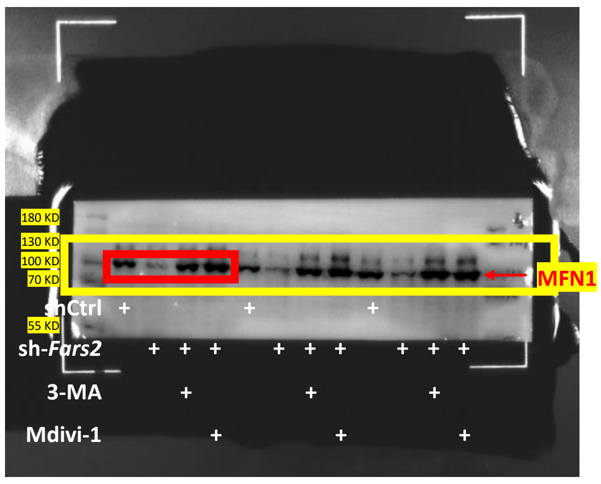

MFN2

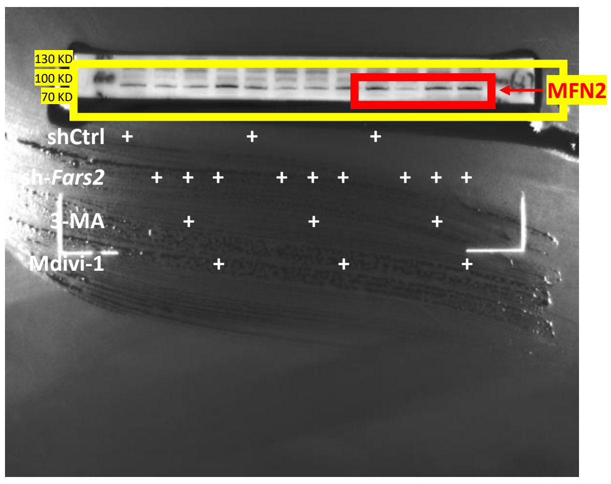

GAPDH

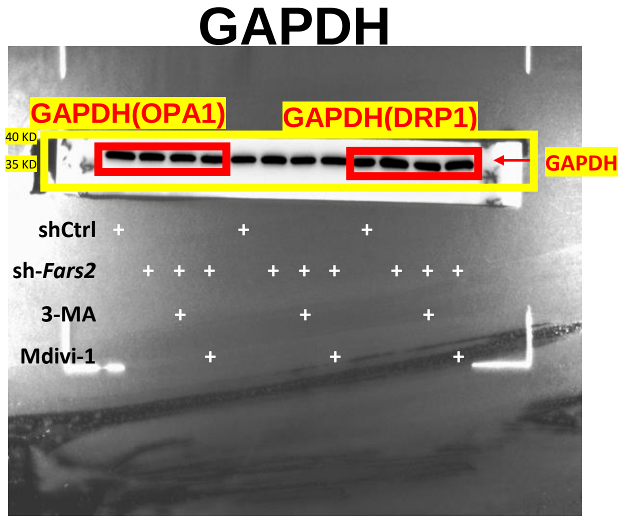

GAPDH (MFN1)

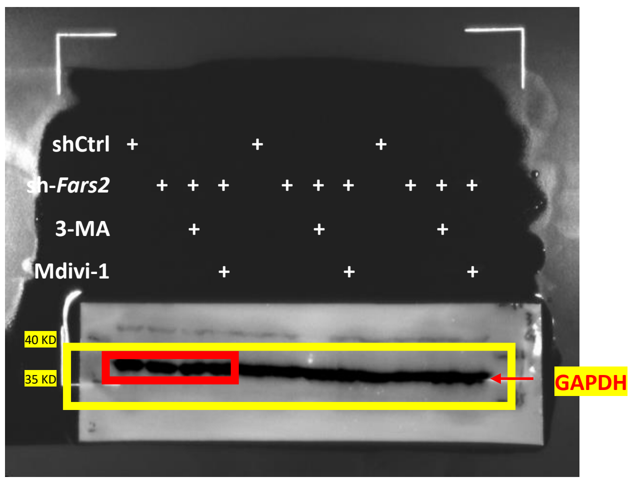

GAPDH (MFN2)

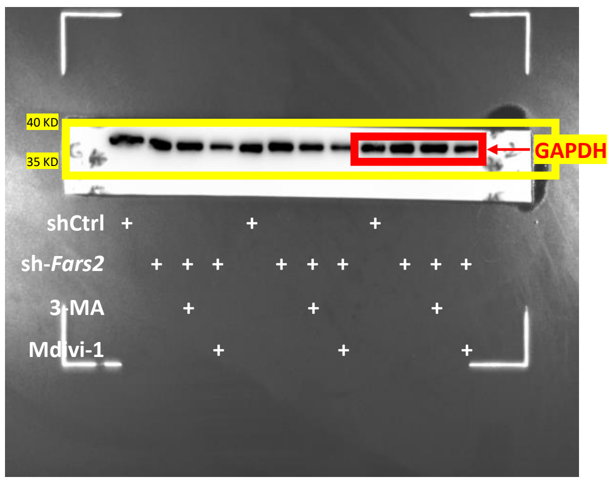

Full unedited gel for Figure S17A

FARS2

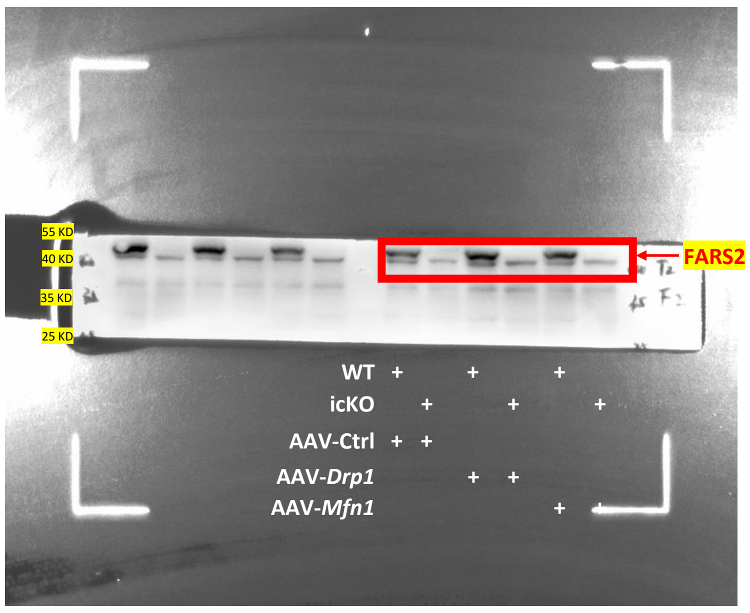

DRP1

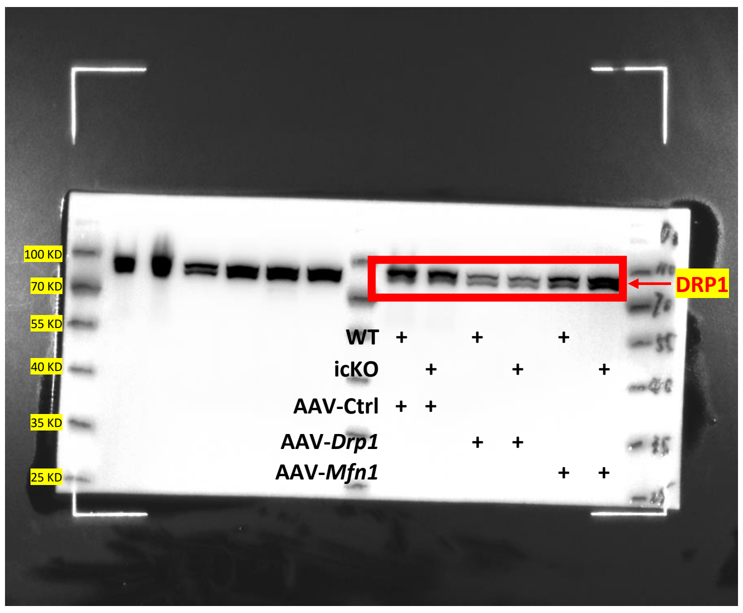

MFN1

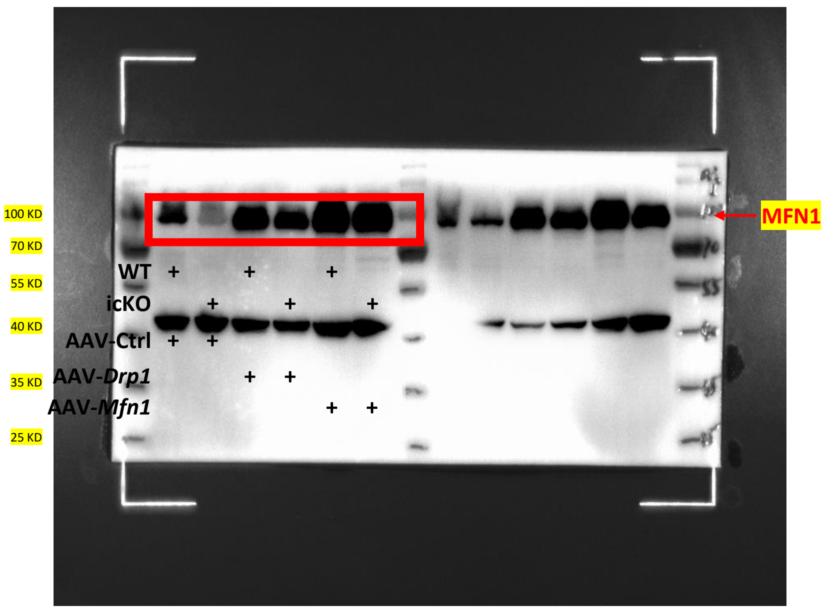

GAPDH (DRP1)

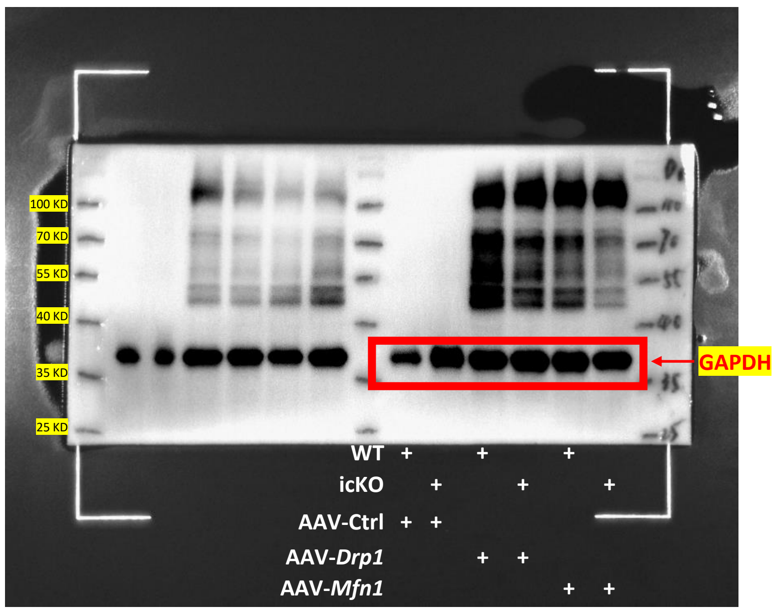

GAPDH (MFN1)

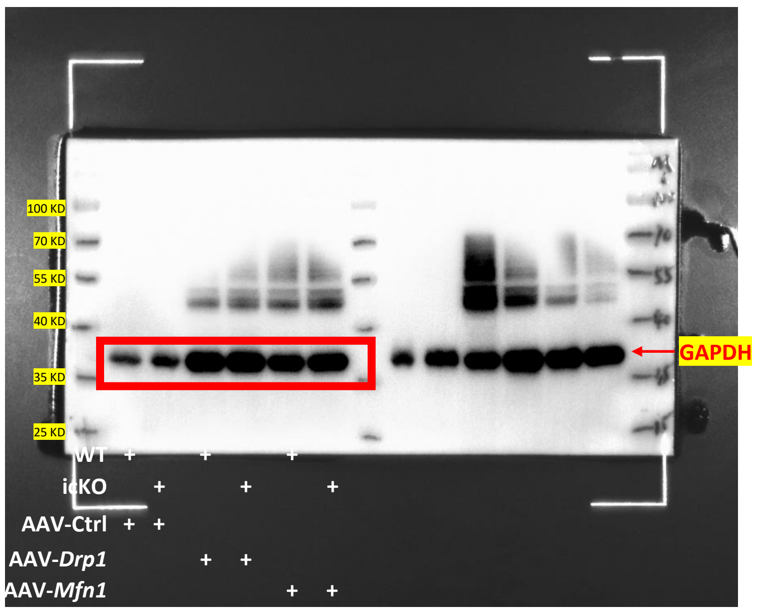

Supplement: Supplementary file 2 [file cir-149-1268-s002.pdf]
